# Design and synthesis of new trimethoxylphenyl-linked combretastatin analogues loaded on diamond nanoparticles as a panel for ameliorated solubility and antiproliferative activity

**DOI:** 10.1080/14756366.2022.2116016

**Published:** 2022-09-26

**Authors:** Islam Zaki, Amal M. Y. Moustafa, Botros Y. Beshay, Reham E. Masoud, Mohammed A. I. Elbastawesy, Mohammed A. S. Abourehab, Mohamed Y. Zakaria

**Affiliations:** a Pharmaceutical Organic Chemistry Department, Faculty of Pharmacy, Port Said University, Port Said, Egypt; b Chemistry Department, Faculty of Science, Port Said University, Port Said, Egypt; c Pharmaceutical Sciences (Pharmaceutical Chemistry) Department, College of Pharmacy, Arab Academy for Science, Technology and Maritime Transport, Alexandria, Egypt; d Clinical Pharmacology Department, Faculty of Medicine, Port Said University, Port Said, Egypt; e Department of Pharmaceutical Organic Chemistry, Faculty of Pharmacy, Al-Azhar University, Assiut, Egypt; f Department of Pharmaceutics, College of Pharmacy, Umm Al-Qura University, Makkah, Saudi Arabia; g Department of Pharmaceutics, College of Pharmacy, Minia University, Minia, Egypt; h Department of Pharmaceutics and Industrial Pharmacy, Faculty of Pharmacy, Port Said University, Port Said, Egypt

**Keywords:** CA-4, cytotoxicity, docking, tubulin, nanodiamond PEG

## Abstract

A new series of vinyl amide-, imidazolone-, and triazinone-linked combretastatin A-4 analogues have been designed and synthesised. These compounds have been evaluated for their cytotoxic activity against MDA-MB-231 breast cancer cells. The triazinone-linked combretastatin analogues (6 and 12) exhibited the most potent cytotoxic activity, in sub-micromolar concentration compared with combretastatin A-4 as a reference standard. The results of β-tubulin polymerisation inhibition assay appear to correlate well with the ability to inhibit β-tubulin polymerisation. Additionally, these compounds were subjected to biological assays relating to cell cycle aspects and apoptosis induction. In addition, the most potent compound **6** was loaded on PEG-PCL modified diamond nanoparticles (PEG-PCL-NDs) and F4 was picked as the optimum formula. F4 exhibited enhanced solubility and release over the drug suspension. In the comparative cytotoxic activity, PEG-PCL modified F4 was capable of diminishing the IC50 by around 2.89 times for nude F4, while by 3.48 times relative to non-formulated compound **6**.

## 1. Introduction

Microtubules are contemplated like a validated goal for the elaboration of new chemotherapeutic entities^1^^,^^2^. Tubulin is the building block of microtubules which are important in cellular functions, such as separation of chromosomes, motility regulation, cell signalling as well as the maintenance of cell shape^3–5^. Combretastatin-A4 (CA-4) and its analogues bind to the colchicine binding site of tubulin to inhibit microtubule assembly, leading to rapid vascular shutdown and apoptosis in solid tumour^6–9^. Unfortunately, the inferior pharmacokinetics of CA-4 due to its poor solubility confined its clinical development and emboldened the researchers to develop a more water-soluble prodrug form (Combretastatin A-4 phosphate, CA-4P)^10^. Nevertheless, significant adverse effects, such as neurotoxicity and cardiovascular toxicity impinged the clinical progress of CA-4P^11^. For this purpose, it is crucial to discover other CA-candidates with enhanced pharmacokinetic and pharmacological characteristics.

Knowing that heterocycles play a crucial role in cancer chemotherapy^12–16^ numerous rationale modifications were made on CA-4, for example, replacing the unstable olefinic bond of CA-4 with heterocyclic scaffold as linkers including pyridine^17^, triazoles^18^, thiazole^19^, 1,2,4-triazolo-4,3-b-pyridazines^20^, furan^21^, imidazole^22^, isoxazole^23^ and other heterocycles^24–28^. The ethylenic bridge is considered to be crucial in conferring the two phenyl rings of CA-4 at the right dihedral angle to optimise the binding with the target. As such, pertaining the both cytotoxicity and antitubulin activity of the proposed CA-4 analogues could be accomplished by replacement of the olefinic bond by heterocycles that promote a cis-locked confirmation^23–28^. On the other hand, several structural modifications were made on the phenyl ring (B) that included variant compositions of hydroxyl, methoxy, and other substituents^28^^,^^29^. Inarguably, these pursued strategies have resulted in the development of perhaps more potent CA-4 analogues.

However, the conventional anticancer therapy limitations as harsh adverse effects and poor aqueous solubility or bio permeation that hinder its efficacy arise the urgency for the evolution of novel anticancer therapy based on the evolvement of nanotechnology as a drug carrier Nanodiamonds (NDs) as a potential carbon based nano platform are one of the crucial strategies to resolve the obstacles that face most of the anticancer drugs owing to NDs various functionalization due to their dominant physical and chemical properties, huge surface area, superior biological safety and compatibility along with their great adsorption capabilities^30–32^. Unfortunately, NDs possess the tendency to aggregate or adhere to biological proteins in the body on its surface which impedes the cellular uptake and discharge escape of the drug-loaded on NDs^33^. The employment of NDs surface coating with either amphiphilic polymer or surfactant could efficiently resolve the disadvantages of NDs^30^. PEG-PCL diblock copolymer capable to achieve enhanced drug loading capacity, promoting drug solubility, anticancer activity, and pharmacokinetic characteristics^34^^,^^35^. Hence, administration of PEG-PCL modified NDs possesses dual advantages which are improving the limitation of NDs and enhancing the solubility, cytotoxic activity, and oral bioavailability of the anticancer drugs.

**Figure 1.**
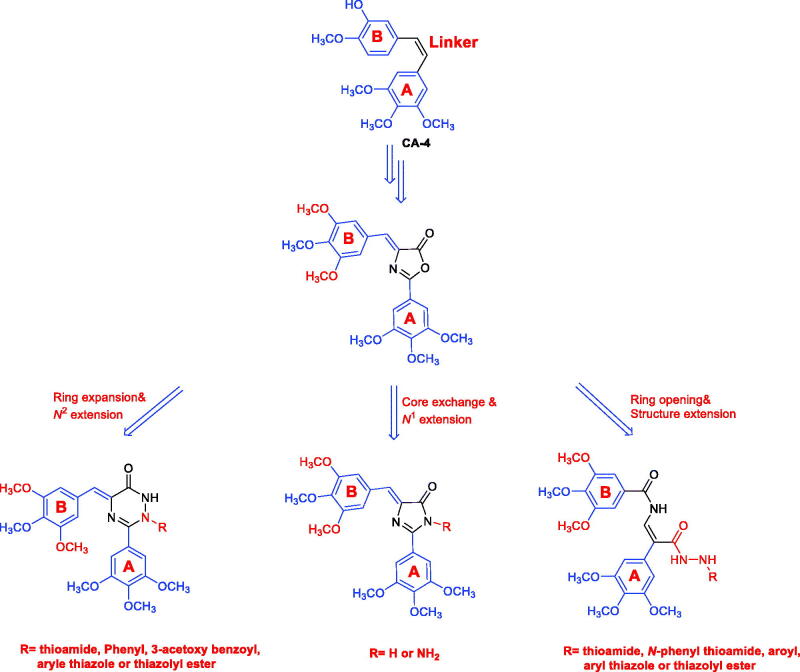
Chemical structures of CA-4 and the designed vinylamide-, imidazolone-, and triazinone-linked CA-4 analogues.

In this context, we aim to investigate the impact of introducing trimethoxy groups on the phenyl ring (B) of CA-4, besides, incorporation of heterocycles and vinyl amides as linker between the two phenyl rings. More importantly, we are engaged in the link-bridge whose rationale modifications may be water-soluble, to enhance the pharmacokinetic characteristics of CA-4 analogues and boosted their potential cytotoxicity and antitubulin activity. In depth, diverse of structural modifications were made on CA-4 to afford CA-4 analogues with trimethoxy phenyl rings linked by vinyl amide moiety, imidazolone, or triazinone rings (Figure 1). Furthermore, the most potent lead compound was loaded on the optimised PEG-PCL modified nanodiamonds (PEG-PCL-NDs) to enhance its aqueous solubility, thus promoting its bioavailability and cytotoxic activity.

## 2. Results and discussion

### 2.1. Chemistry

**Scheme 1.**
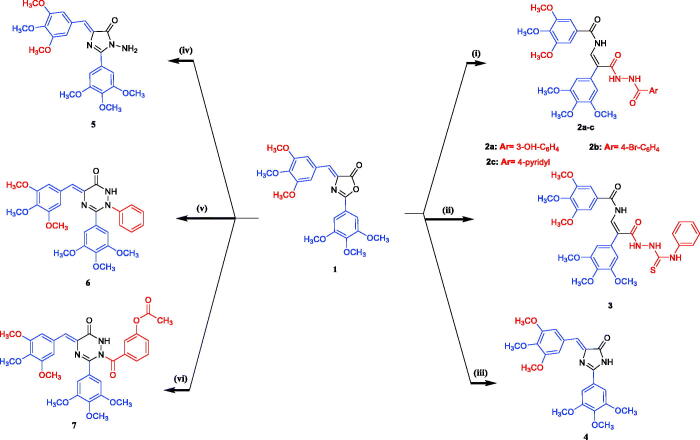
General synthetic route for compounds **2–7**. Reagents and reaction condition: (i) appropriate aryl carboxylic acid hydrazide, EtOH, reflux 7–8 h; (ii) 4-phenylthiosemicarbazide, EtOH, reflux 8 h; (iii) NH_4_OH, reflux 24 h, (iv) NH_2_NH_2_.H_2_O, EtOH, AcOH, reflux 6 h; (v) phenyl hydrazine, EtOH, reflux 6 h; (vi) 3-hydroxybenzoic acid hydrazide, acetic anhydride, reflux 8 h.

**Scheme 2.**
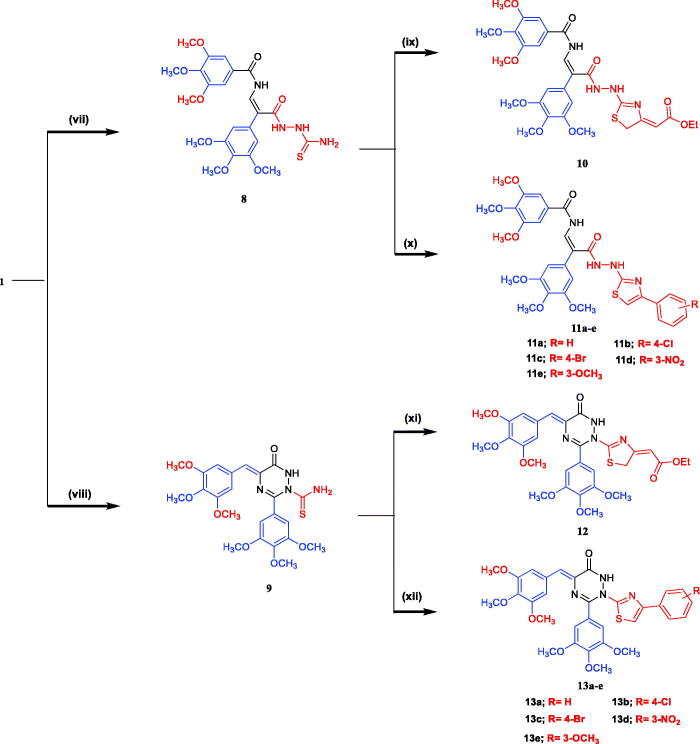
General synthetic route for compounds **8–13e**. Reagents and reaction condition: (vii) thiosemicarbazide, EtOH, AcOH, reflux 8 h; (viii) thiosemicarbazide, NaOAc, AcOH, reflux 8 h; (ix) ethyl 4-chloroacetoacetate, NaOAc, EtOH, reflux 6 h; (x) appropriate phenacyl bromide, NaOAc, EtOH, reflux 2–3 h. (xi) ethyl 4-chloroacetoacetate, NaOAc, EtOH, reflux 8 h; (xii) appropriate phenacyl bromide, NaOAc, EtOH, reflux 3–4 h.

The synthesis of the prepared compounds **2–13e** is shown in Schemes 1 and 2. The starting material 4-(3,4,5-trimethoxybenzylidene)-2-(3,4,5-trimethoxyphenyl)oxazol-5(4*H*)-one **(1)** was prepared according to the reported procedure^36^ *via* treatment of 2-(3,4,5-trimethoxybenzamido)acetic acid with 3,4,5-trimethoxybenzaldehyde in the presence of acetic anhydride containing anhydrous pyridine. The IR spectrum of **1** showed the presence of a characteristic absorption band attributed to the carbonyl group (C = O) of lactone ring at 1820 cm^−1^. The interaction of oxazolone **1** with aryl carboxylic acid hydrazide in boiling ethanol afforded the corresponding aroyl hydrazide derivatives **2a–c**. The *N*-phenyl thioamide **3** was obtained by reacting oxazolone **1** with 4-phenylthiosemicarbazide in refluxing ethanol for 8 h. In addition, aminolysis of **1** with ammonia led to the formation of imidazolone **4.** The *N*-amino imidazolone derivative **5** was obtained by treatment of **1** with hydrazine hydrate *via* ring-opening reaction. The ring opening of **1** was substantiated by spectroscopic data, the IR spectrum of compound **5** showed bands at cm^−1^ attributed to NH and NH_2_ groups. Additionally, treatment of **1** with phenylhydrazine in ethanol at reflux temperature afforded a good yield of the corresponding *N*-phenyl-1,2,4-triazinone derivative **6**. The structure of compound **6** was confirmed based on IR, ^1^H-NMR, and ^13^C-NMR spectra. The ^1^H-NMR spectrum of **6** showed the signal of the NH group at *δ* 9.12 ppm in addition to the extra aromatic protons signals in the range of *δ* 6.72–7.90 ppm. The ^13^C-NMR spectrum of **6** showed the signal carbonyl group at *δ* 169.71 ppm and the aromatic carbon signals at *δ* 106.38–159.51 ppm. The condensation of oxazolone **1** with 3-hydroxybenzoic acid hydrazide in refluxing acetic anhydride afforded *N*-(3-acetoxybenzoyl)-1,2,4-triazinone derivative **7**. Assignment of structure **7** is based on different spectral data, such as IR, ^1^H-NMR, and ^13^C-NMR. The ^1^H-NMR spectrum of compound **7** showed signals at *δ* 11.36 ppm of NH group, *δ* 7.04–7.88 of aryl rings, and *δ* 1.90 ppm of CH_3_ group of acetoxy group. ^13^C-NMR spectrum of compound **7** showed signals at *δ* 165.53–172.60 ppm attributed to three carbonyl groups (2C = O of CONH and C = O of ester) and at *δ* 21.70 ppm of the methyl group of acetoxy function. Furthermore, when compound **1** was allowed to react with thiosemicarbazide in refluxing absolute ethanol the corresponding hydrazide carbothioamide **8** was obtained. Similarly, upon applying the same previous procedure using glacial acetic acid in the presence of anhydrous sodium acetate instead of absolute ethanol the product was identified as *N*-thioamide-1,2,4-triazinone derivative **9**. Finally, the thioamide group in compounds **8** and **9** was subjected to cyclisation reaction by refluxing with ethyl 4-chloroacetoacetate or phenacyl bromide derivatives in absolute ethanol in the presence of anhydrous sodium acetate to produce the corresponding thiazolyl molecules **10–13e**, respectively. The structures of these compounds were confirmed by the analytical and spectral data.

### 2.2. *In vitro* biological activity studies

#### 2.2.1. Cytotoxic activity against breast MDA-MB-231 cell line

All the synthesised compounds were screened for their cytotoxic activity against breast MDA-MB-231 cancer cell line and compounds **6** and **12** against breast MCF-10A cell line taking CA-4 as a positive control for the analysis. The *in vitro* cytotoxicity results of the prepared compounds were systematised in Table 1. As shown in Table 1 most of the synthesised molecules demonstrated moderate to highly potent cytotoxic activities over MDA-MB-231 breast cancer cell line. Triazinone-linked CA-4 analogues **6** and **12** displayed potent cytotoxic activity with IC_50_ values of 1.36 and 1.71 µM, respectively comparative to CA-4 IC_50_ (IC_50_ = 0.55 µM). Structurally, the prepared molecules contain three structural variations; including rotatable hydrazide derivatives, the core exchange with imidazolone ring, and the ring expansion with triazinone motif. Regarding the non-cyclized hydrazide derivatives, it can be observed that compound **10** displayed the most potent cytotoxic activity with an IC_50_ value of 4.80 µM. In addition, the imidazole derivatives were the least potent cytotoxic molecules against breast MDA-MB-231 cancer cells. In the case of triazinone ring, the *N*2-phenyl triazinone derivative **6** showed promising greater activity against breast MDA-MB-231 cancer cells rather than the *N*2-(3-acetoxybenzoyl) **7** or *N*2-thioamide derivative **9**. Finally, regarding thiazolyl derivatives **10–13e**; thiazolyl ester molecule **12** revealed a higher antiproliferative activity (IC_50_ = 1.71 µM) compared with other thiazolyl derivatives. It is noticed that directly attached benzene ring to triazinone scaffold (compound **6**) or bioisosteric group (thiazolyl ester) as in compound **12**, may contribute to the astonishing cytotoxicity against breast MDA-MB-231 cancer cells. Furthermore, cleavage of triazinone core and/or replacement of the compound **12** ester group with an extra aromatic ring markedly decrease the cytotoxic activity (derivatives **10**, **11a–e**, **13a–e**), with the exception of compound **13d** which pertain moderate cytotoxic activity (IC_50_ = 3.16 µM)

**Table 1.** Cytotoxic activity of synthesised compounds against breast MDA-MB-231 and MCF-10A cell lines.

Comp No	IC_50_ value (µM)	
	MDA-MB-231	MCF-10A
**2a**	21.70 ± 1.17	NT
**2b**	39.72 ± 1.80	NT
**2c**	13.22 ± 0.71	NT
**3**	14.93 ± 0.80	NT
**4**	36.84 ± 2.81	NT
**5**	57.09 ± 3.23	NT
**6**	1.36 ± 0.10	19.52 ± 2.15
**7**	13.66 ± 1.04	NT
**8**	31.46 ± 1.82	NT
**9**	4.14 ± 0.22	NT
**10**	4.80 ± 0.21	NT
**11a**	6.212 ± 0.47	NT
**11b**	8.59 ± 2.38	NT
**11c**	25.7 ± 1.90	NT
**11d**	12.15 ± 0.65	NT
**11e**	>100	NT
**12**	1.71 ± 0.11	14.64 ± 1.96
**13a**	19.64 ± 1.42	NT
**13b**	20.72 ± 1.58	NT
**13c**	10.14 ± 0.54	NT
**13d**	3.16 ± 0.17	NT
**13e**	33.48 ± 2.47	NT
**CA-4**	0.55 ± 0.09	8.91 ± 0.71

NT: not tested.

#### 2.2.2. *In vitro* tubulin polymerisation assay

Trimethoxy phenyl fragment-containing natural stilbenoid-derived molecules, such as Colchicine and CA-4 bind to β-tubulin subunit at the colchicine binding site resulting in inhibition of microtubule assembly and suppression of its formation^37^. To confirm whether the most potent compounds **6** and **12** similarly target the tubulin system, compounds **6** and **12** were evaluated for their *in vitro* inhibition of β-tubulin polymerisation at their IC_50_ concentrations for each compound. The inhibitory activity of β-tubulin polymerisation is presented in Table 2. From the obtained results, compounds **6** and **12** were found to decrease the level of tubulin compared with CA-4 with percentage inhibition of 77.61 and 68.28%, respectively. From the obtained results it can be concluded that the antiproliferative activity of the synthesised triazinone-linked CA-4 derivatives may be derived from the inhibition of microtubule assembly.

**Table 2.** Percentage inhibition of β-tubulin polymerisation on MDA-MB-231 cell line for compounds **6, 12** and CA-4 at their IC_50_ (µM).

Comp No	% Inhibition of β-tubulin polymerisation	MDA-MB-231 IC_50_ (µM)
**6**	77.61 ± 0.10	1.36 ± 0.10
**12**	68.28 ± 0.16	1.71 ± 0.11
**CA-4**	87.05 ± 0.08	0.55 ± 0.09

#### 2.2.3. Cell cycle analysis

**Figure 2.**
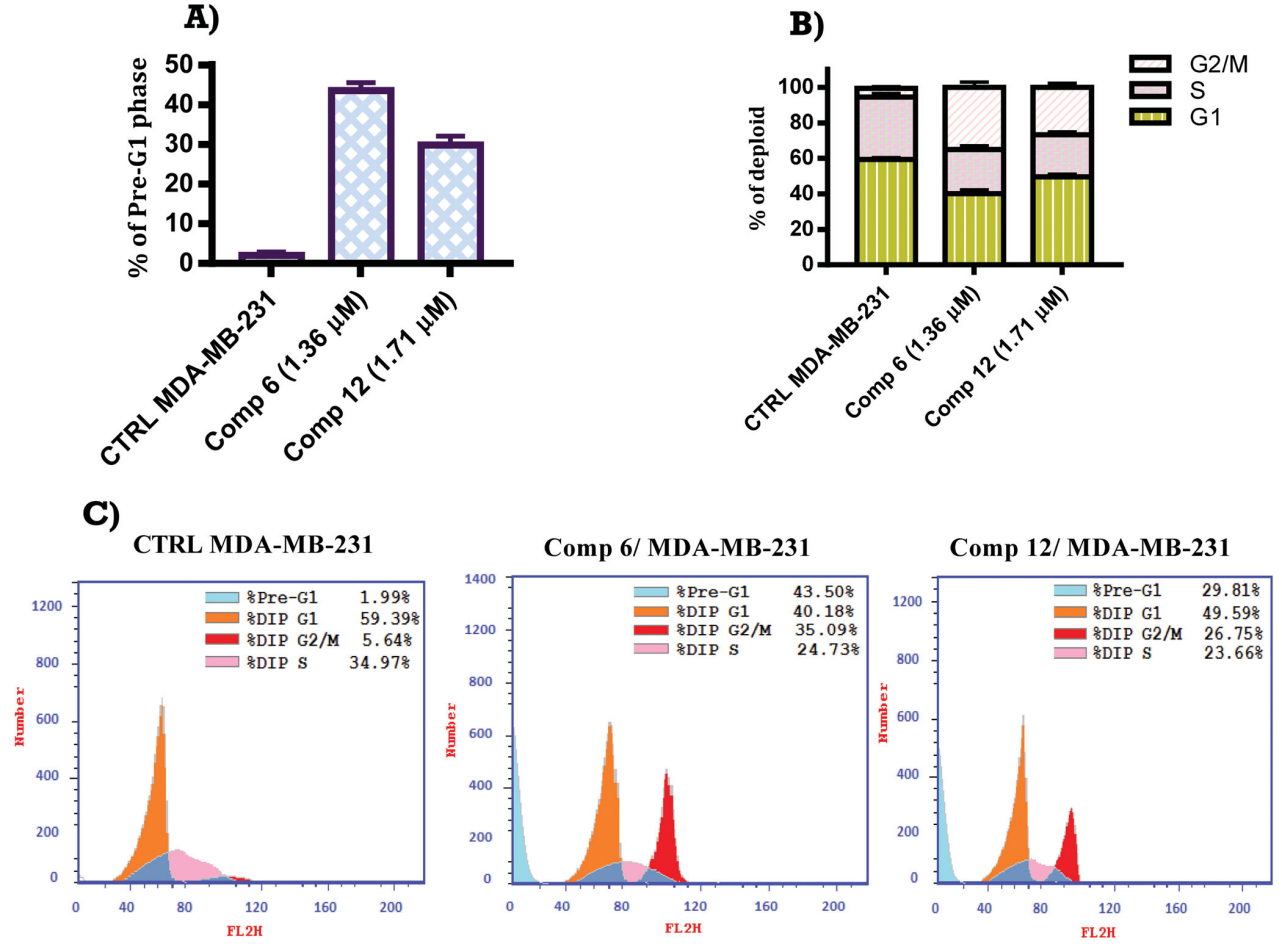
(A) FACS analysis of cell cycle distribution percentage of MDA-MB-231 cells after treatment with compounds **6** and **12** at their IC_50_ (µM) for 24 h relative to untreated control. (B) Graphical representation of FACS analysis of cell cycle distribution percentage of MDA-MB-231 cells after treatment with compounds **6** and **12** at their IC_50_ (µM) for 24 h relative to untreated control.

Targeting the cell cycle division at distinct checkpoints of cancer cells has been identified as a promising strategy for cancer chemotherapy^38^. To investigate the effect of our designed compounds on cell cycle progression and induction of apoptosis compounds **6** and **12** showed the highest cytotoxic affect against MDA-MB-231 breast cancer cell lines were subjected to cell cycle analysis using DNA flow cytometry assay. MDA-MB-231 cells were treated with compounds **6** and **12** at a concentration equal to their IC_50_ values for 24 h. The results shown in Figure 2 revealed that compounds **6** and **12** led to pre-G1 apoptosis and arrested cell cycle at G2/M phase in MDA-MB-231 cells. Meanwhile, compounds **6** and **12** treated cells showed 35.09 and 26.75% in G2/M phase, respectively relative to the untreated control group (5.64%). Also, it was found that compounds **6** and **12**-treated cells increased from 1.99 to 43.50 and 29.81%, respectively at pre-G1 apoptosis indicating that, compounds **6** and **12** caused apoptosis in MDA-MB-231 cells.

#### 2.2.4. Apoptosis studies

##### 2.2.4.1. *In vitro* apoptosis staining assay

**Figure 3.**
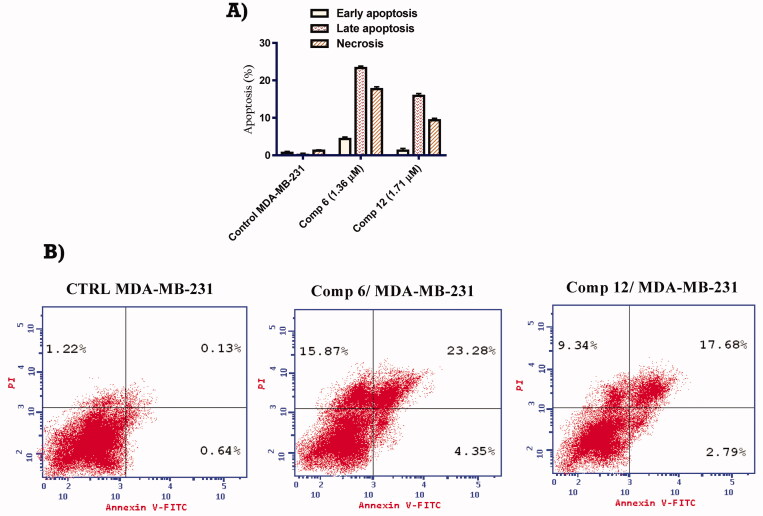
(A) Graphical representation of the effect of compounds **6** and **12** on induction of apoptosis in MDA-MB-231 cells after 24 h. (B) Effect of compounds **6** and **12** on induction of apoptosis in MDA-MB-231 cells after 24 h.

The effect of compounds **6** and **12** on apoptosis percentage rate was carried out using annexin V-FITC/Propidium iodide (PI) biparametric cytofluorimetric double staining analysis in MDA-MB-231 cells. In this test, MDA-MB-231 cells were incubated with compounds **6** and **12** at a concentration equal to their IC_50_ values for 24 h. The results depicted in Figure 3 showed that the percentage of total apoptotic cells in MDA-MB-231 cells was increased after treatment with compounds **6** and **12** by 21.86- and 14.98-fold, respectively compared with no treatment control. Additionally, the percentage of early apoptotic cells was increased after treatment with compounds **6** and **12** by 6.80- and 4.36-fold, respectively relative to no treatment control. Moreover, the percentage of late apoptotic cells in MDA-MB-231 cells was increased after treatment with compounds **6** and **12** to 23.28 and 17.68%, respectively. compared to 0.13% for untreated control.

##### 2.2.4.2. *In vitro* ELISA measurement of level of apoptosis-related proteins

**Figure 4.**
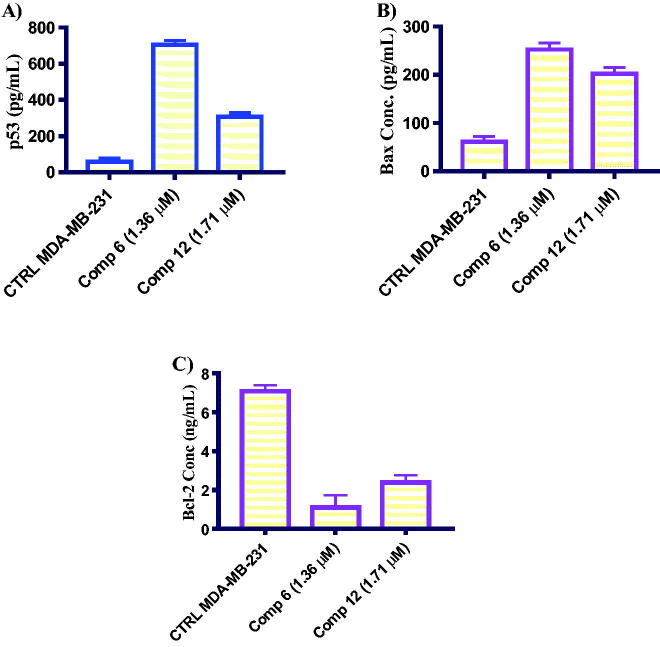
Graphical representation of the effect of compounds **6** and **12** on apoptosis-related proteins after 24 h. (A) p53 level, (B) Bax level, and (C) Bcl-2 level.

p53, Bax, and Bcl-2 are key regulators of the apoptosis process to keep the balance between viable and dead normal cells^39^. Deregulation of these proteins is a common feature responsible for uncontrolled cancer cell growth^38^. In this test, the protein expression of apoptotic regulators was examined using ELISA analysis. The results were shown in Figure 4. Treatment of MDA-MB-231 cells with compounds **6** and **12** at their IC_50_ concentration for 24 h was found to increase the level of apoptotic proteins (p53 and Bax) compared with no treatment control with fold change values 11.96-, 5.20-, 4.13- and 3.31-, respectively. Similarly, the level of Bcl-2 was decreased by 6.49- and 2.96-fold, respectively compared with no treatment control. Based on these results, compounds **6** and **12** enhanced the intrinsic apoptotic pathway in MDA-MB-231 cells *via* increasing the level of p53 and Bax as well as decreasing the level of Bcl-2.

##### 2.2.4.3. *In vitro* caspase 3/7 analysis

**Figure 5.**
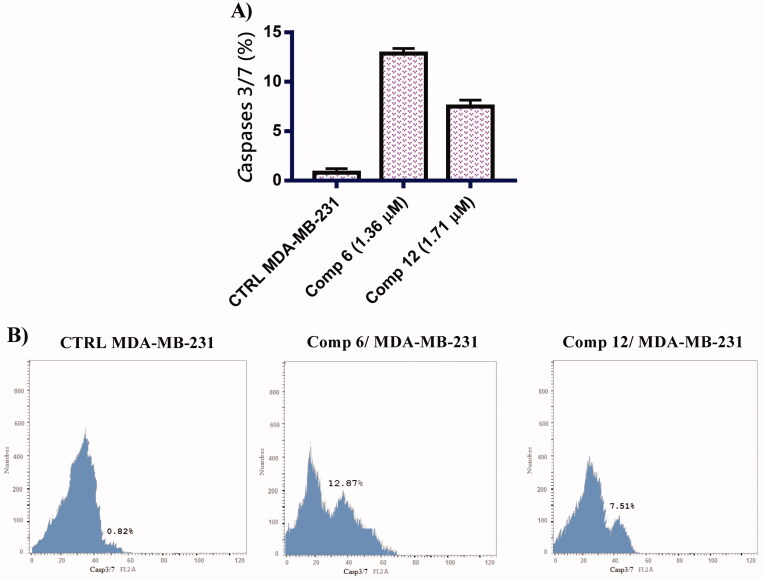
(A) Graphical representation of *c*aspase 3/7 analysis related to apoptosis in MDA-MB-231 cells treated with compounds **6** and **12** at their IC_50_ concentration (B) caspase 3/7 analysis related to apoptosis in MDA-MB-231 cells treated with compounds **6** and **12** at their IC_50_ concentration.

Further, it is important to check effector caspase 3/7. The cleavage of caspases is specifically responsible for programmed cell death and apoptosis^40^. MDA-MB-231 cells were treated with compounds **6** and **12** at their IC_50_ concentration for 24 h and the percentage of activated caspase 3/7 was observed using green FACS analysis. The results were shown in Figure 5 and the expression level of caspase 3/7 was increased when compared with no control cells. In addition, compounds **6** and **12** produced about 15.70- and 9.16-fold increase in caspase 3/7 activation, respectively relative to the untreated control group. This finding confirms that compounds **6** and **12** enhanced the level of caspase 3/7 revealing the occurrence of apoptosis in MDA-MB-231 breast cancer cells.

### 2.3. Molecular modelling study

**Figure 6.**
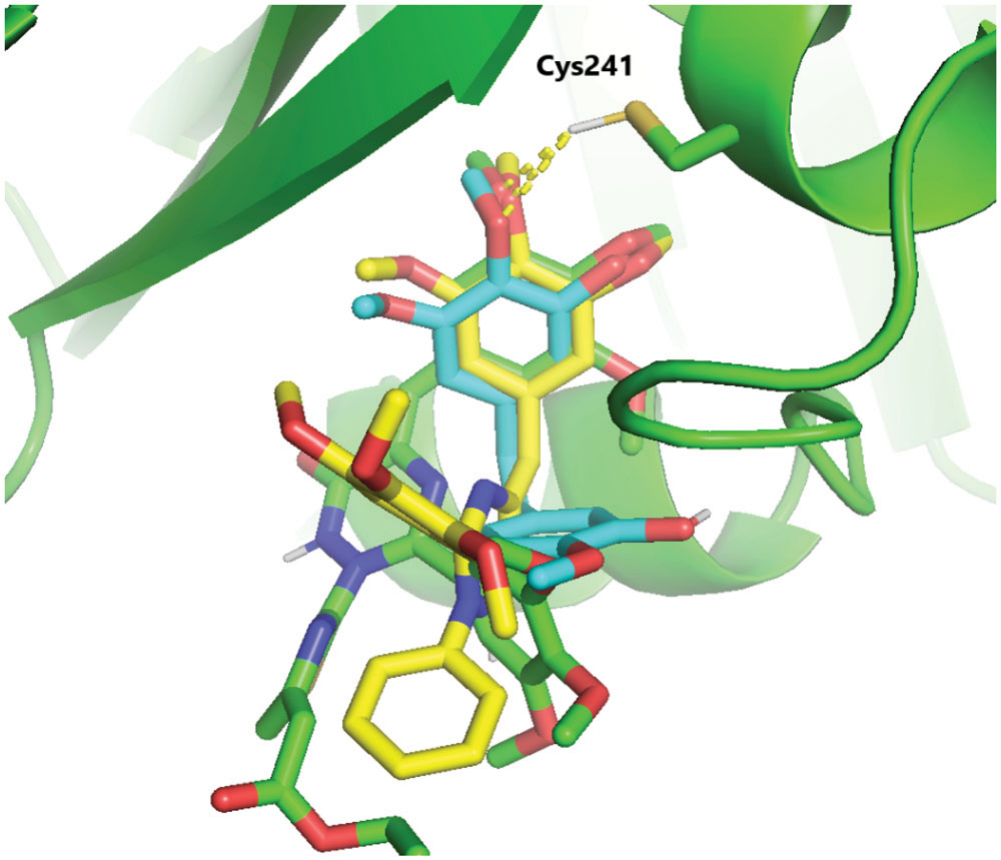
Superposition of **CA-4** (Cyan), **6** (yellow), and **12** (green) in the colchicine-binding pocket of tubulin in cartoon representation. Hydrogen bond with the key amino acid residue Cys241 is shown to be conserved in **CA-4** and its analogues **6** and **12**.

**Figure 7.**
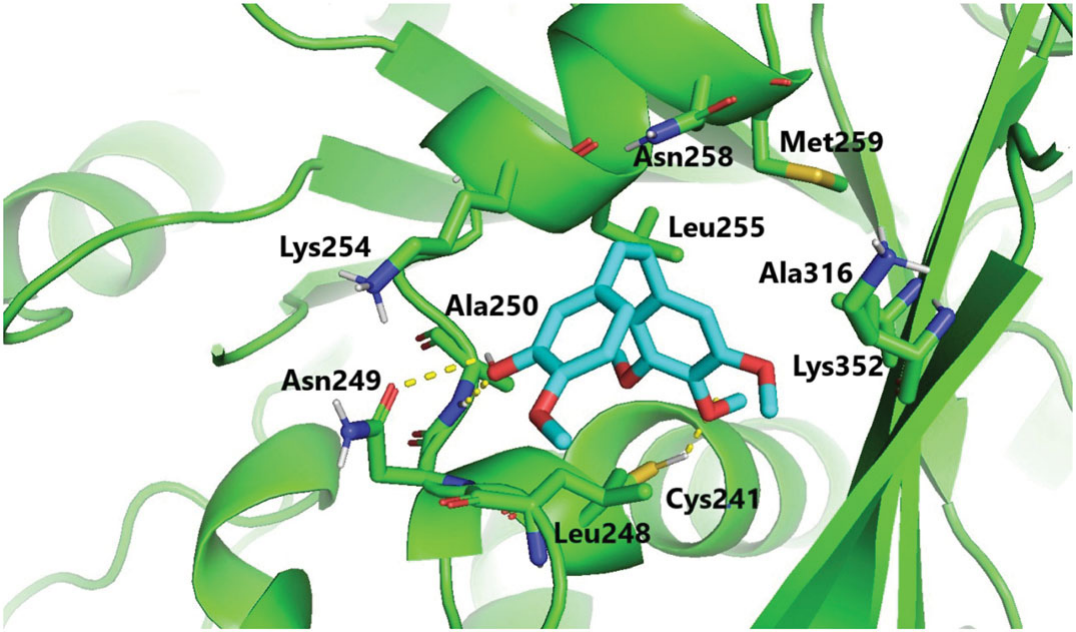
Binding mode of **CA-4** in the colchicine-binding pocket of tubulin. Hydrogen bonds are shown by yellow dashed lines.

**Figure 8.**
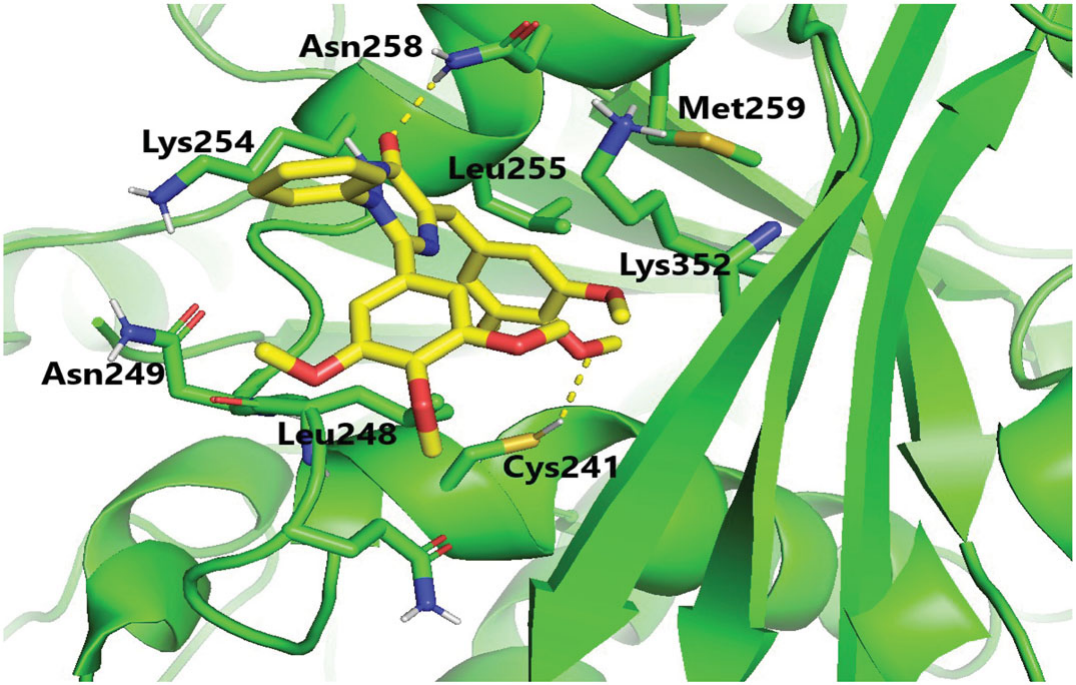
Binding mode of compound **6** in the colchicine-binding pocket of tubulin. Hydrogen bonds are shown by yellow dashed lines.

**Figure 9.**
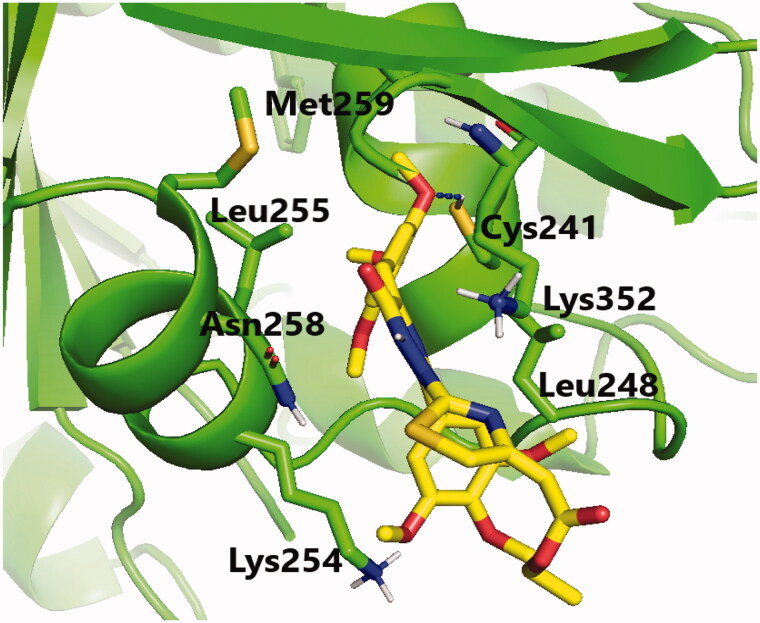
Binding mode of compound **12** in the colchicine-binding pocket of tubulin. Hydrogen bonds are shown by blue dashed lines.

Molecular docking studies were conducted to explain the outstanding cytotoxicity and tubulin inhibitory activity of triazinone-linked combretastatin analogues (**6** and **12**) relative to **CA-4**. Interestingly, **CA-4**, **6**, and **12** are superimposed and perfectly positioned into colchicine binding site of tubulin (Figure 6) with binding free energies of −6.2, 6, and −5.8 k cal/mol, respectively. In depth, **CA-4**, **6**, and **12** (Figures 7–9) formed H-bond with the crucial amino acid residue Cys241. Additionally, strong hydrophobic interactions with Gln247, Leu248, Ala250, Lys254, Leu255, Asn258, Lys352, and Ala316 were mapped in the tubulin pocket. It is worth mentioning that H-bond with Cys241 and the hydrophobic interactions with previously mentioned amino acid residues side chains, as well as other polar interactions with Leu248, Met259, and Lys254 played a very important role during the protein-ligand binding process, possibly illustrating the outstanding tubulin inhibitory activity of compounds CA-4 and traizinone-linked CA-4 analogues (**6** and **12**)^17^^,^^41^^,^^42^.

However, the extra H-bonds formed between the hydroxy functionality of **CA-4** and Asn249, Ala250 (Figure 7) could explain the slightly superior inhibitory activity of **CA-4** relative to compounds **6** and **12**. More interestingly, compound **6** exhibited the highest tubulin inhibitory activity amongst the designed compounds that could be attributed to the aforementioned hydrophobic and hydrophilic network, besides the new H-bond formed with NH_2_ functionality of amino acid residue Asn258 (Figure 8). Additionally, the increased hydrophobic contacts between the *N2*-phenyl ring and the sidechain of amino acid residues Lys352 and Lys254 could stabilise compound **6** in the binding pocket. Regarding compound **12** (Figure 9), the polar interactions formed between thiazole-traizinone scaffold and the hydrophilic pocket flanked by amino acid residues Lys352, Asn258, Lys254, Asn249 seems to stabilise the ligand in the binding site and compensated the loss of H-bond with Asn256, leading to slightly decrease in the inhibitory activity of compound **12** (68.24%) compared to compound **6** (77.54%).

These docking results suggest that cytotoxicity of the traizinone-linked **CA-4** analogues (**6** and **12**) against the MDA-MB-231 breast cancer cell line could be attributed to their antitubulin polymerisation activities.

### 2.4. Elaboration of PEG-PCL modified diamond nanoparticle (PEG-PCL-NDs)

#### 2.4.1. In silico *predictive ADME study for targeted compound (6)*

**Figure 10.**
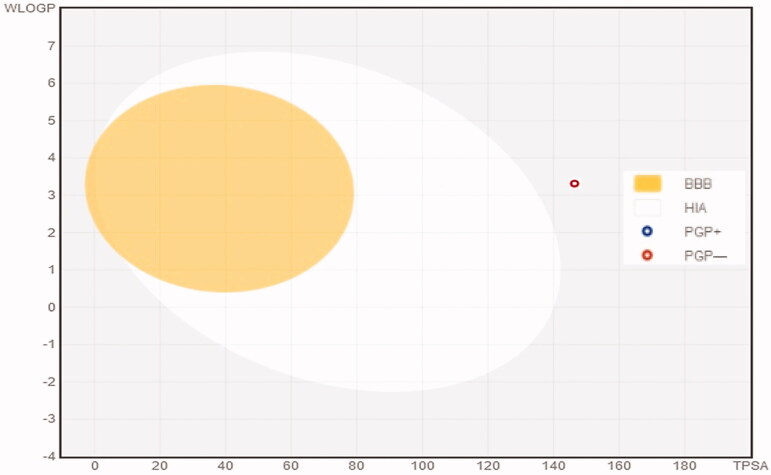
Human intestinal absorption (HIA) and Blood–brain barrier (BBB) plot for **6**.

The analysis of the lead chemical 6's Lipinski drug-likeness and structure was manoeuvred using the free web-based program Swiss ADME (http://www.swissadme.ch/), which correlated to the in-silico pharmacokinetic features of the drug. Absorption level, aqueous solubility level (LogS-SILICOS-IT), lipophilicity [Log P (LogP 98)], blood–brain barrier level (BBB LEV), 2D polar surface area (2D TPSA), and probability of cytochrome P450 2D6 (CYP2D6) and cytochrome P450 2D6 (CYP PROB) inhibition were among the characteristics evaluated. Figure 10 showed the acquired outcomes as an ADMET plot with calculated W log P 98 and TPSA-2D characteristics.

The HIA and BBB plots for human intestinal absorption (HIA) were assessed for compound **6**. Figure 10 exploited the BBB plot, **6** falling outside the 99 and 95% ellipses, as indicated by the dots on the graph, as the capability of the drug molecules to permeate passively through the BBB hinting that the drug molecules disability to pass through the blood-brain barrier. Furthermore, the red circle in Figure 10 indicated that the drug molecule was not a P-glycoprotein substrate. As a result, compound **6** could be inclined to diminish the undesired consequences on CNS. In terms of the HIA plot, Figure 10 revealed that compound **6** is thought to enable modest intestinal absorption owing to the capacity of massive drug amount to passively traverse the intestine compared to BBB. The compound's anticipated decreased water-solubility (LogS-SILICOS-IT = −8.53; 1.53 × 10^–6^ mg/mL; 2.97 × 10^–9^ mol/l) was governed as a major concern that could obstruct its full intrinsic oral bioavailability and biological cytotoxic activity. The anticipated oral bioavailability (PSA = 146.1) verified this. Generally, compounds with PSA > 140 have a low bioavailability, therefore compound **6** has one, as its anticipated oral bioavailability (PSA = 146.1). The inhibitory and non-inhibitory capability of compound **6** on the Cytochrome P450 2D6 enzyme was confirmed by the CYP2D6 value. Even more, anticipated to inhibit CYP2D6, thus, it was a high possibility to predispose to adverse effects, such as liver dysfunction along with drug-drug interactions upon administration.

Based on the previously mentioned ADME study outcomes, NDs were proposed as a potential drug carrier for promoting the aqueous solubility, hence the oral bioavailability of water-insoluble molecules could be also enhanced as NDs possess vast alluring characteristics, such as their huge surface area which can be also easily modified, the capability of enhancing the drug solubility along with obtaining sustained drug release pattern, low cost of production and processing^43^. However, NDs suffer from stability problems owing to their aggregation tendency, thus well dispersed disaggregated NDs in aqueous can be sparsely attained which in turn limits its cellular uptake and its biological application^44^. The addition of an amphiphilic copolymer or surfactant could be one of the proposed solutions to this problem^45^. Surface modification of NDs with PEG-PCL might enhance drug intestinal absorption, and attain sustained drug release besides its ability to extend retention time^46^. PEG-PCL as an amphiphilic polymer can surround the NDs with a brushy layer that opposes their tendency to adhere and aggregate, moreover, it possesses good biocompatibility, biodegradability, and broadly utilised in micellization for solubilisation^46^^,^^47^. Thus, tailoring of compound **6,** which displayed the highest cytotoxic activity and tubulin inhibitory activity, as PEG-PCL modified NDs was supposed to enhance its cellular uptake and being dispersed at the post of the tumour, thus augment its antitumor activity.

#### 2.4.2. Design of experiment, tailoring, and statistical assessment of 6-PEG-PCL modified nanodiamonds (6-PEG-PCL-NDs)

The impact of the fabrication variables on the assumed outcomes was investigated adopting 2^3^ full factorial designs. This experimental design predisposes to the evolution of 8 experimental runs and their related outcomes: DL%, PS, PDI, and ZP were tabulated in Table 3. The convenient model precision value of the model with a ratio exceeding four is acquired which was noticed for all the dependent variables as demonstrated in Table 4. The proximity of adjusted and predicted R2 by a value not exceeding 0.20 of each other denotes the accuracy and precision of the design and that their values of all dependent outcomes were in good harmony with each other (Table 3). Drug assessment at different concentrations was implemented using HPLC at *λ*_max_ 254 nm and conditions previously reported^48^.

**Table 3.** Experimental runs, independent variables, and estimated responses of 6-loaded PEG-PCL-NDs *via* 2^3^ full factorial experimental.

Formula	A (ND Conc. mg/ml)	B (PEG-PCL amount	C (PC amount)	Y1 (DL%)	Y2 (PS)	Y3 (PDI)	Y4 (ZP)
F1	1	20	25	58.4 ± 3.3	212.8 ± 19.5	0.43 ± 0.04	−21.5 ± 4.2
F2	4	20	25	73.6 ± 2.8	156.3 ± 21.6	0.32 ± 0.05	−28.7 ± 6.7
F3	1	60	25	67.8 ± 3.4	276.4 ± 26.4	0.35 ± 0.06	−35.8 ± 8.1
F4	4	60	25	87.3 ± 2.4	201.2 ± 18.7	0.23 ± 0.05	−39.1 ± 3.9
F5	1	20	50	66.2 ± 4.1	253.8 ± 23.2	0.51 ± 0.08	−27.2 ± 5.1
F6	4	20	50	79.8 ± 4.3	222.7 ± 28.9	0.37 ± 0.04	−32.9 ± 4.6
F7	1	60	50	75.1 ± 5.1	304.5 ± 20.1	0.39 ± 0.05	−43.2 ± 7.2
F 8	4	60	50	94.3 ± 4.8	275.1 ± 16.8	0.27 ± 0.064	−47.6 ± 7.3

**Table 4.** 2^3^ Factorial investigation outcome of **6**-loaded PEG-PCL-NDs and the predicted, observed responses and deviation percent of the Optimum formula (F4).

Responses	DL (%)	PS (nm)	PDI	ZP (mV)
*R* ^2^	0.986	0.956	0.99	0.984
Adjusted *R*^2^	0.976	0.923	0.983	0.971
Predicted *R*^2^	0.946	0.83	0.96	0.934
Adequate precision	27.98	16.1	32.86	24.34
Significant factors	A, B, C	A, B, C	A, B, C	A, B, C
Observed value of the optimal formula (F4)	87.3	201.2	0.23	−39.1
Predicted value of the optimal formula (F4)	86.2	215.3	0.22	−40.9
Absolute deviation %	1.26	7	4.3	4.6

##### 2.4.2.1. Impact of the tailoring variables on DL%

**Figure 11.**
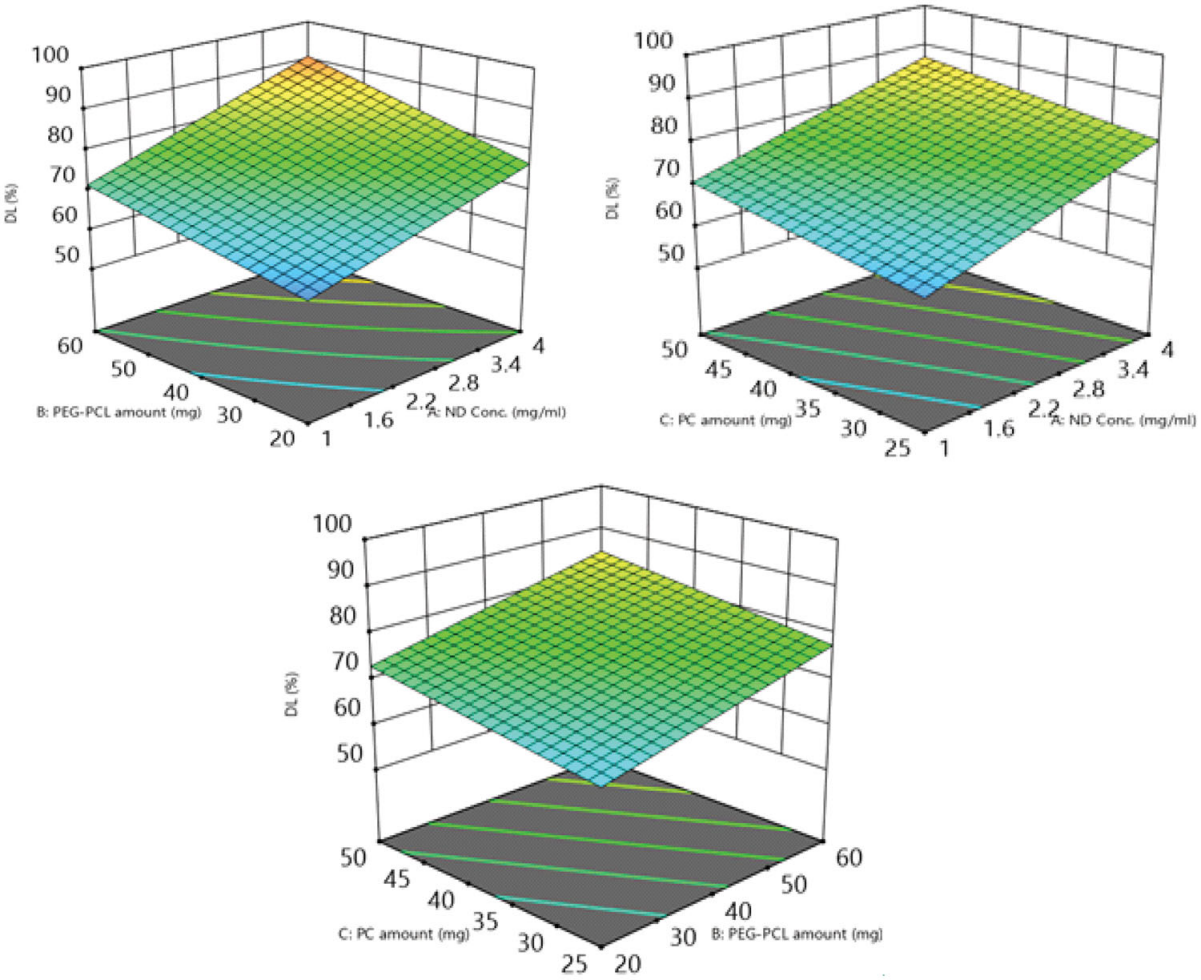
The impact of (A) ND concentration, (B) PEG-PCL amount, (C) PC amount on EE percent of 6-loaded PEG-PCL-NDs is displayed *via* 3D surface response plots.

The capability of the fabricated PEG-PCL modified NDs to enclose the drug was assessed *via* estimation of DL% which in turn gives a hint on the ability of the system as a successful carrier for compound **6**. The percentage of **6** disclosed in the nano carriers ranged from 58.4 ± 3.3 to 94.3 ± 4.8% as prevailed in Table 2. The influence of the fabrication variables NDs concentration (A), PEG-PCL amount (B), PC amount (C) on DL% is graphically displayed in 3-D surface plots (Figure 11).

Concerning NDs concentration (A), formulae possessing 4 mg/mL nanodiamond exploited a significant (*p* = 0.0002) higher drug loading relative to formulae consisting of 1 mg/mL. The higher concentration of NDs allows a greater surface for the drug molecules to be absorbed *via* physical adsorption and to be stacked in the interspace of the nanodiamond clustering^43^. Furthermore, increasing the NDs permits a higher possibility for hydrogen bonding or Vander Waals forces to be occurred between the drug and NDs surface, hence a higher opportunity for drug loading^32^^,^^43^. Meanwhile, at lower NDs concentration not enough ND were available for drug loading, thus the DL% was decreased.

Concerning PEG-PCL amount (B), increasing the polymer amount from 20 to 60 mg predisposed to a significant increase (*p* = 0.0008) in DL%. These results can be justified by PEG-PCL possibly to form a coat surrounding the drug-NDs complex, thereby, this coating prohibited the drug leakage from the nano-complex^49^.

Considering the PC (Factor C), increasing its amount from 25 to 50 mg arise to a superiority (*p* = 0.0051) in DL%. This could be attributed to the higher PC content could potentiate the alignment of the polymer-modified nano-complex core surrounded by PC multilayers, thus granting a larger space for the drug to be ligated into these bilayers; Moreover, PCs enhance the rigidity of nano-complex thus prohibiting drug leakage^50^.

##### 2.4.2.2. Impact of the tailoring variables on NDs size (PS) and polydispersity index (PDI)

**Figure 12.**
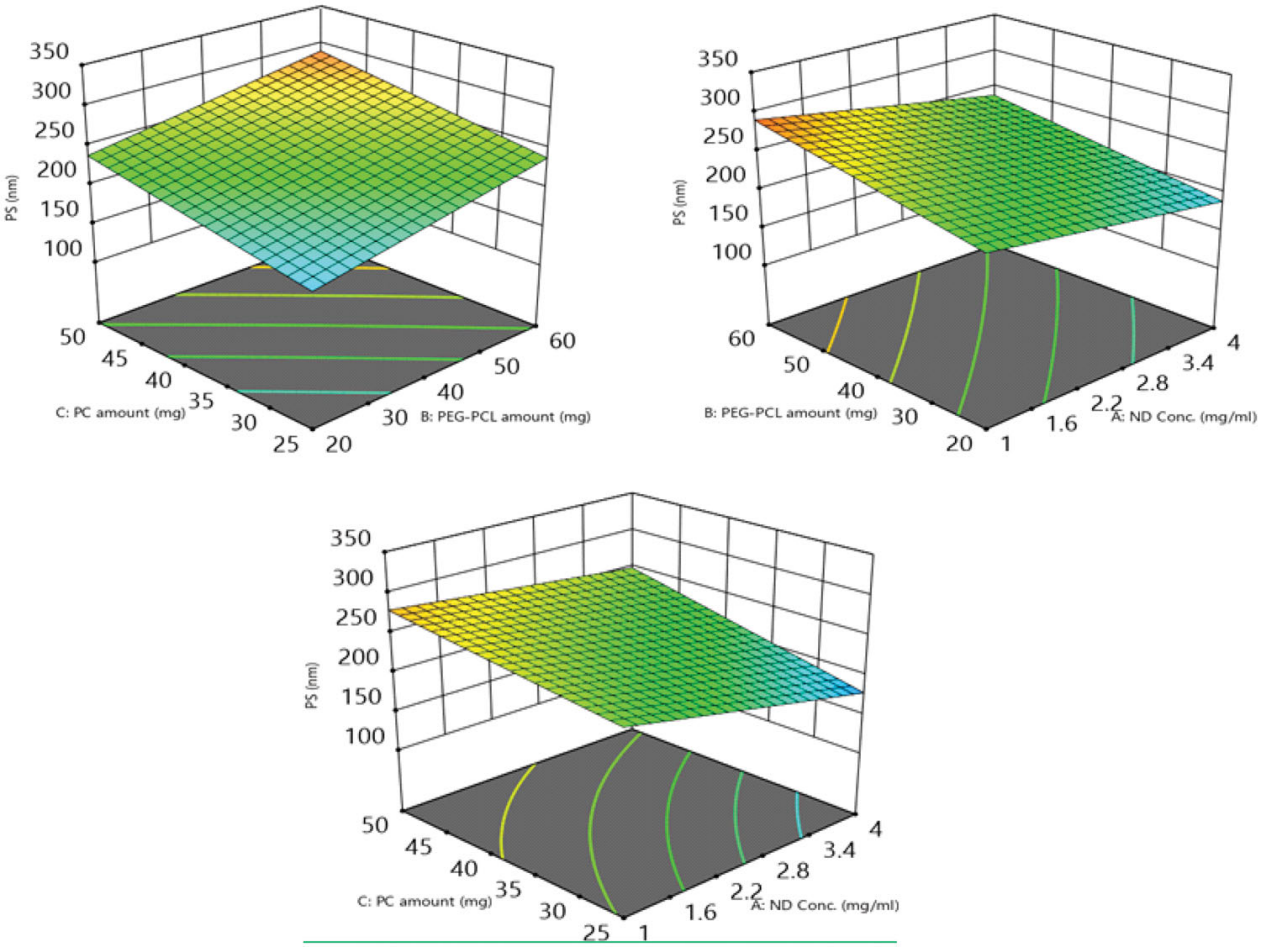
The impact of (A) ND concentration, (B) PEG-PCL amount, (C) PC amount on PS percent of 6-loaded PEG-PCL-NDs is displayed *via* 3D surface response plots.

**Figure 13.**
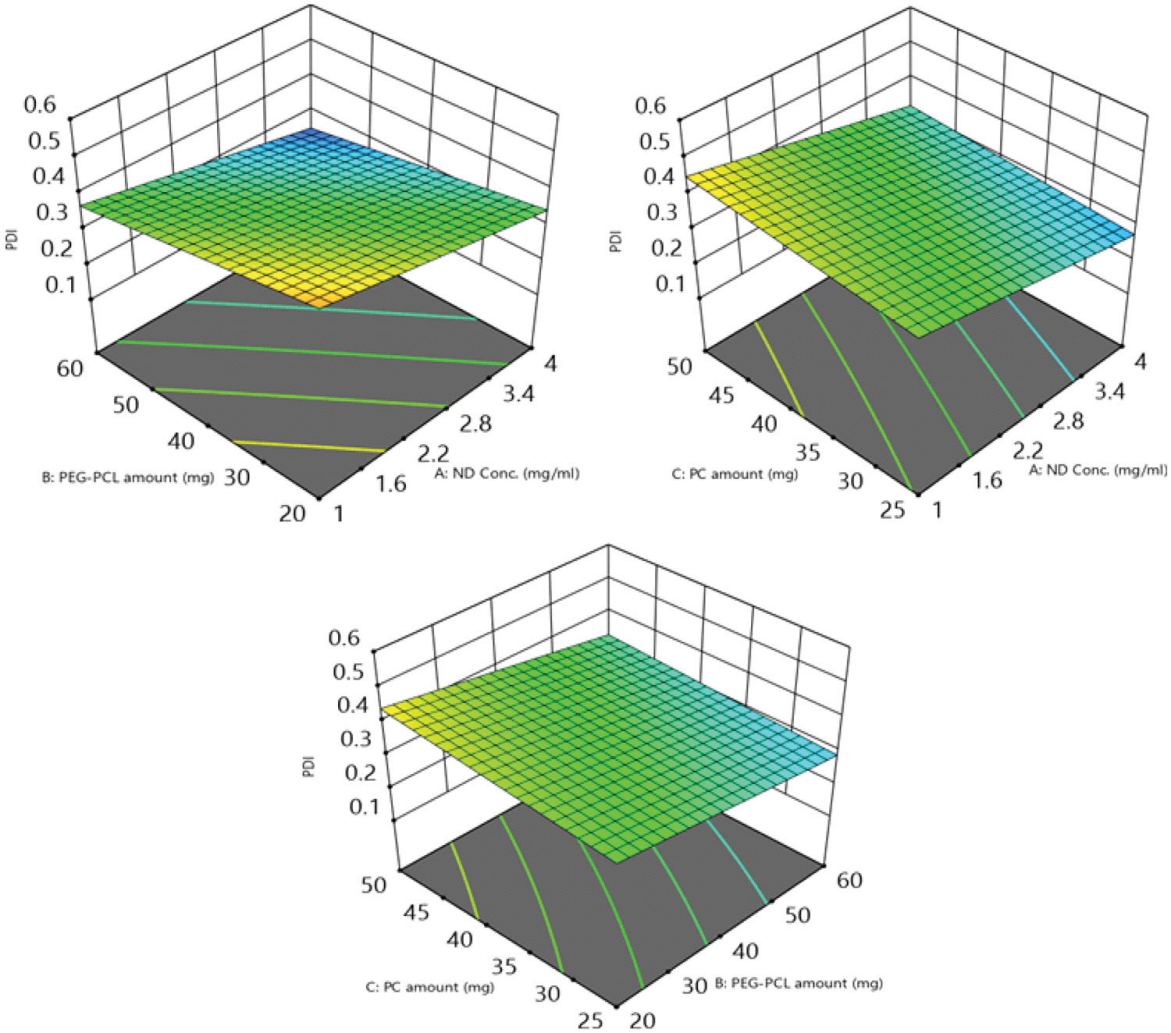
3D surface response plots exploit the impact of (A) ND concentration, (B) PEG-PCL amount, and (C) PC amount on PDI of 6-loaded PEG-PCL-NDs.

The extent of homogeneity of the diameter of the particles can be anticipated by PDI values, where proximate PDI values to zero imply to monodispersity, meanwhile, values proximate to 1 imply to polydispersity (PS). The PDI values of the formulated NDs shown in Table 2 were in the range of 0.23 ± 0.05 to 0.51 ± 0.08. Thus, the PDI values align towards homogeneity with a convenient range^51^. Meanwhile, The PS of the fabricated NDs ranged from 156.3 ± 21.6 to 304.5 ± 20.1. The influence of the fabrication variables: NDs concentration (A), PEG-PCL amount (B), PC amount (C) on PDI and PS graphically represented in 3-D surface plots (Figures 12 and 13), respectively.

Concerning, ND concentration (A), increasing the concentration of NDs from 1 to 4 mg/mL results in a significant decrease in PDI and PS (*p* = 0.0001 and 0.0073), respectively, and thus higher extent of homogeneity at lower NDs concentration the individual ND suffers from diminished colloidal stability owing to its tendency for aggregation and precipitation^44^.

On another hand increasing the amount of PEG-PCL (B) from 20 to 60 mg predisposes to a significant decline in PDI and PS values (*p* = 0.0003 and 0.0051), respectively. The surface coating of NDs with surfactant or polymer can be considered as one of the efficient strategies adopted to overcome the instability of ND dispersion due to aggregation, where the coat creates a repellent layer that prevents the nano-complex to get closer and adhere to each other. Thus, at a lower concentration of PEG-PCL incomplete coat that fully covered the NDs surface leading to a massive increase in PDI and PS values. Meanwhile, increasing the PEG-PCL amount results in the formation of nano-complex of lower PDI and PS owing to the PEG-PCL amount was sufficient to complete the NDs surface^49^.

Regarding Factor C, increasing the number of PC results in t a significant elevation in PDI and PS (*p* = 0.0032 and 0.0053), respectively. This outcome was in agreement with the outcomes of Aldawarsi et al.^52^, who reported the subsequent increase in PS of the formulated raloxifene nanocarrier on increasing PC concentration. This may be attributed to the assembly of multiple bilayers which subsequently can accommodate a higher amount of the drug, hence, increasing both PS and the DL%. Also, the location of a PC at the borders of the coated nano-complex can also permit the NDs to attain a larger amount of the target drug without being leaked out, predisposing them to larger NDs sizes^50^^,^^53^.

##### 2.4.2.3. The impact of the tailoring variables on ZP

**Figure 14.**
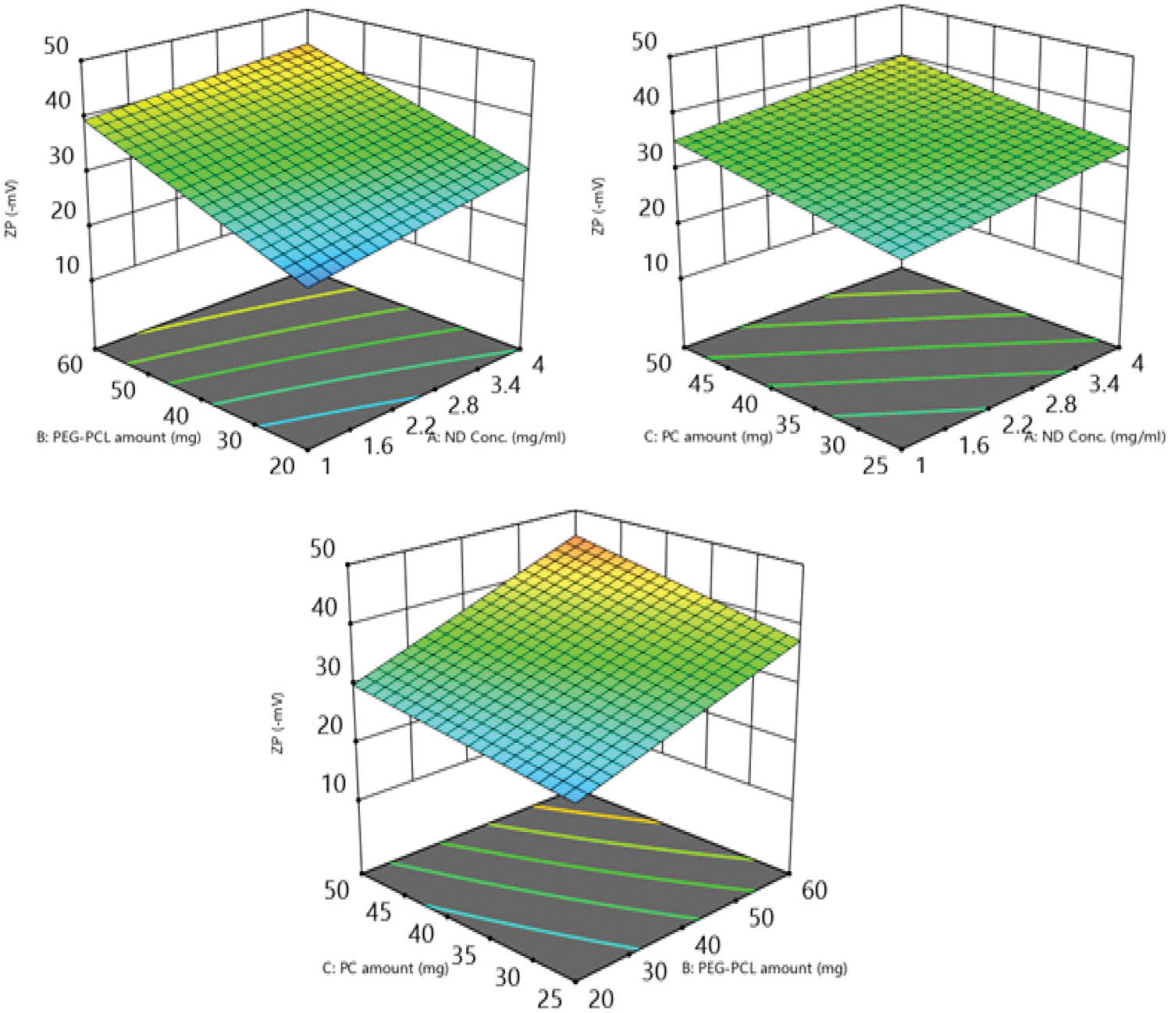
The impact of (A) ND concentration, (B) PEG-PCL amount, and (C) PC amount on ZP percent of 6-loaded PEG-PCL-NDs is displayed *via* 3D surface response plots.

Basically, a high absolute value of ZP, could achieve adequate electrostatic repulsive forces among the dispersed nanocarriers and predisposing to a sustained stability^54^. In the implemented experiment, the estimated ZP values form the fabricated 7-PEG-PCL-NDs ranged from −21.5 ± 4.2 to −47.6 ± 7.3 mV as demonstrated in Table 2. The impact of the tailoring variables on the ZP of six loaded formulae is graphically demonstrated in 3-D surface plots (Figure 14).

Regarding the impact of factor, A, increasing the concentration of NDs results in a subsequent significant (*p* = 0.0079) increase in the absolute value of ZP. This may be attributed to the consequent increase in the surface area of NDs that bear negatively charged functional groups which are responsible for the highly negative charge of NDs^49^. These results were in agreement with Cheng et al., who also found that a higher concentration of NDSs increases the values of ZP^49^.

Moreover, increasing the amount of PEG-PCL (B) from 20 to 60 mg leads to significantly higher negative ZP values for six loaded formulae (*p* = 0.0002). This can be justified by the fact that the anionic polymer PEG-PCL due to its functional group was capable to create a negatively charged layer around the nano-complexes^35^. Thus, the higher amount of PEG-PCL results in a thicker coat surrounding the nanoparticles which in turn will densify the electronegative charges on the NDs surface. According to Muthu et al., assumed that the negatively charged polymeric coating predispose to a significant negative charge on the nanoparticle surfaces relative to the nude one^55^.

The ANOVA outcomes disclose that increasing the amount of PC (Factor C) from 25 to 50 mg will significantly (*p* = 0.0006) leads to higher negative charges allocated on the surface of NDs. As previously reported in El-Zaafarany et al. that the assembly of the PC bearing negative charges on the outer layer leads to a higher negative charge of the nano-carriers^56^. Accordingly, increasing the amounts of PC will elevate the overall values of the ZPs^53^.

#### 2.4.3. The optimisation and statistical validity of the optimum 6-loaded PEG-PCL-NDs

**Figure 15.**
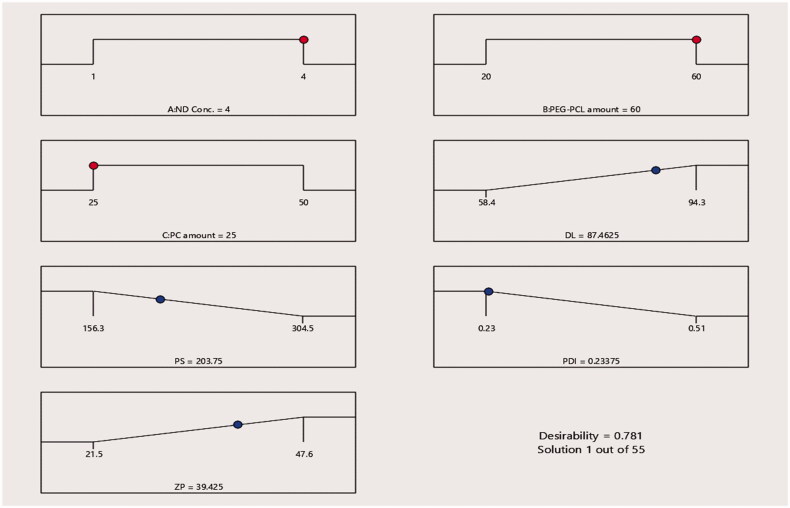
Optimisation ramps for the studied independent variables exploit the optimum criteria of the formulation variables for 6-loaded PEG-PCL-NDs formulae with the predicted value of each measured formulation parameter.

According to the statistical analysis outcomes conducted by Design expert software, the optimum formula possessed 4 mg/mL ND, 60 mg PEG-PCL, and 25 mg PC with a desirability value of 0.767 was attained. Furthermore, based on the percent of discrepancy among the predicted and the observed values of %DL, PS, PDI, and ZP which are capable of assessing the validity of the design, it can be concluded that the consistency of the statistical design to results assessment owing to the small percent of discrepancy as an absolute value (<10%) illustrated in Table 4. Figure 15 arise the optimum criteria for **6**-loaded PEG-PCL modified NDs.

#### 2.4.4. *In vitro* assessment of the optimized 6-Loaded PEG-PCL modified NDs

##### 2.4.4.1. Differential scanning calorimetry (DSC)

**Figure 16.**
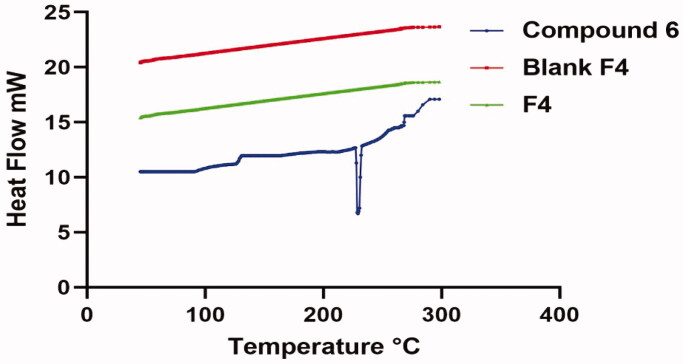
DSC thermograms: Blue: Thermogram of pure compound **6**, Red: Thermogram of blank formula (F4), and Green: Thermogram of the optimum **6**-loaded PEG-PCL modified NDs (F4).

Figure 16 revealed the pure form of both compound **6** thermal attitude, blank lyophilised formula and the optimised lyophilised **6**-loaded PEG-PCL modified NDs (F4). **6** thermal attitudes exploit a sharp characteristic endotherm at ∼230.2 °C which is correlated to its melting point, meanwhile, this characteristic peak was vanished in DSC thermogram of lyophilised blank and **6**-loaded PEG-PCL modified NDs (F4) anticipating the gross configuration of the formulation components along with compound **6** from a crystalline to amorphous phase referring to the optimum enclosing of the compounds within the nano-complexes.

##### 2.4.4.2. X-ray diffraction (XRD)

**Figure 17.**
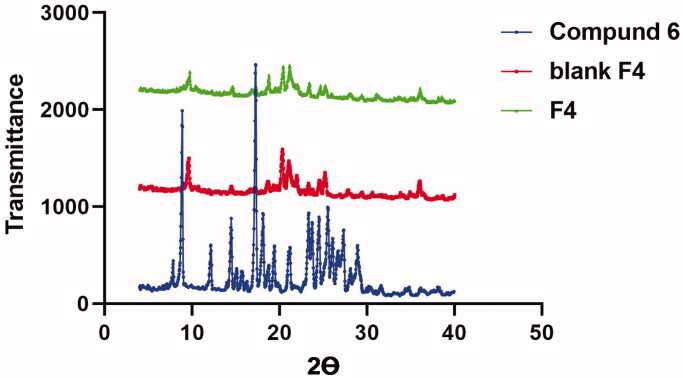
XRD pattern: Blue: Thermogram of pure compound **6**, Red: Thermogram of blank formula (F4), and Green: Thermogram of the optimum **6**-loaded PEG-PCL modified NDs (F4).

Figure 17 revealed the diffractograms of pure compound **6**, lyophilised blank optimal formula and PEG-PCL modified NDs (F4). The diffractogram configuration of compound **6** exploited well-defined peaks at 2*θ* = 7.8 and 16.8°. On another hand, the XRD of lyophilised blank optimised formula (F4), **6**-loaded PEG-PCL modified NDs (F4) revealed the vanish of the distinct peaks of compound **6** and other components involved in the formulation owing to their conformation to amorphous phase within the nano-complexes^57^.

##### 2.4.4.3. Transmission electron microscope TEM

**Figure 18.**
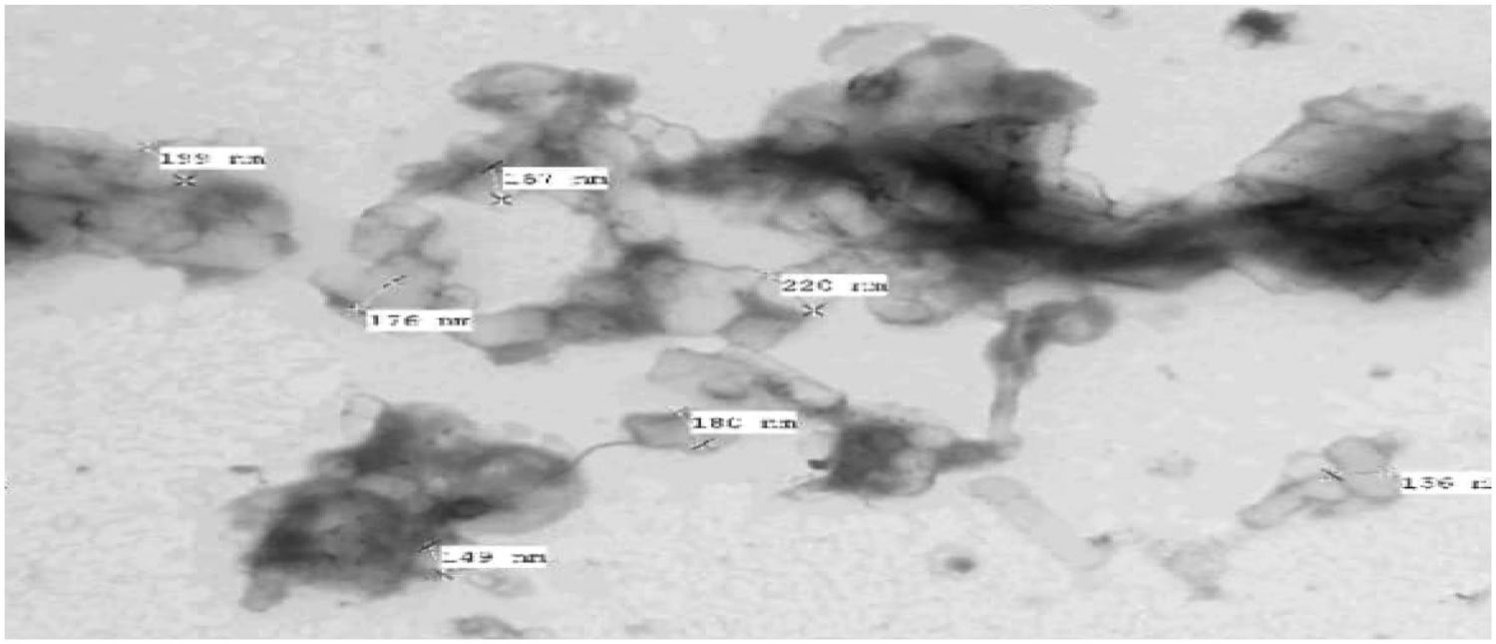
TEM of the elected optimised PEG-PCL modified NDs.

The TEM displayed in Figure 18, The NDs aggregates were slightly uneven in shape with a broad size distribution ranging from 136 to 220 nm with obvious coating darker in colour^49^. Furthermore, the TEM image didn’t prevail any drug crystal confirming the complete configuration of the drug into amorphous form in agreement with the outcomes of the XRD and DSC^58^.

##### 2.4.4.4. Comparative in-vitro drug release study of optimal formula (F4) relative to compound 6 suspension

**Figure 19.**
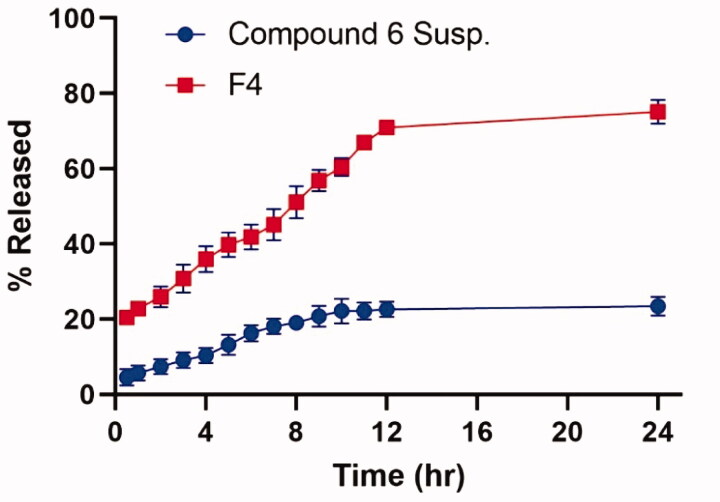
% of **6** released ± *SD* from the optimised PEG-PCL modified NDs (F4) compared to that of **6** suspensions.

*In-vitro* drug release experiment was conducted to simulate the *in-vivo* condition in which the drug will be evolved and dissolved. Figure 19 revealed the progressive release of the drug from the optimised formula (F4) over 24 h, which is characterised by a dual release pattern of around 20% in the first hour followed by extended drug release till 24 h^57^. The cumulative released amount of drug from F4 was 74. 6 ± 3.9% compared to 22. 3 ± 2.7% for **6** suspensions (*p* < 0.05). This can be justified based on the nanoscale size offered by NDs allow a larger surface area to be exposed to the dissolution media, hence increasing the drug wettability. Furthermore, PEG-PCL modification owing to its solubilisation potentiality will aid in enhancing the solubility of the drug, thus promoting the rate and extent of drug release besides offering a reservoir role to extend its release along a wide period of time^49^.

##### 2.4.4.5. Comparative cytotoxic study of optimised PEG-PCL modified NDs (F4) versus nude F4

**Figure 20.**
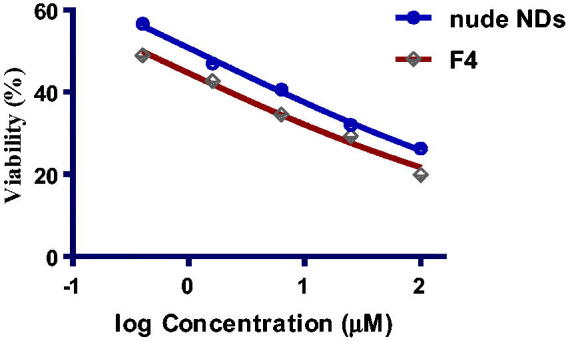
The graphical pattern shows the results of % cell viability of nude F4 versus PEG-PCL-NDs (F4) at varying concentrations (µM) on MDA-MB-231 cancer cells.

To elaborate on the significance of the modification of the surface of NDs with PEG-PCL polymer, the cytotoxicity study was conducted for optimised PEG-PCL modified NDs (F4) relative nude F4. Figure 20 revealed the % viability of MDA-MB-231 cancer cells post-exposure to both formulae. The IC_50_ values of the results were displayed in PEG-PCL modified NDs (F4) and nude F4 were (0.39 ± 0.08 and 1.13 ± 0.21 µM), respectively. The magnitude of cytotoxicity was found to increase on the following trend: Compound **6 **>** **nude F4 > PEG-PCL-NDs F4. PEG-PCL-NDs F4 exhibited the chief cytotoxicity and this may be attributed to the large surface area of NDs, enhanced drug solubility and release along with the promotion of the drug cellular uptake, all of these leading to a boost in the anticancer activity.

## 3. Conclusion

A new series of amide-, imidazolone-, and triazinone-linked combretastatin analogues were designed, synthesised, and evaluated for their cytotoxicity against breast MDA-MB-231 cell line. The tested compounds revealed good activity towards the tested cancer cells especially the traizinone-linked **CA-4** analogues (**6** and **12**) with IC_50_ values of 1.36 and 1.71 µM, respectively. Additionally, the most active compounds (**6** and **12**) were evaluated for their β-tubulin polymerisation inhibitory activity. Tubulin polymerisation inhibition assay findings appear to correlate well with the cytotoxicity data results. Moreover, the conducted docking results suggest that the cytotoxicity of the traizinone-linked **CA-4** analogues (**6** and **12**) against the MDA-MB-231 breast cancer cell line could be attributed to their antitubulin polymerisation activities. More interestingly, pertaining both cytotoxicity and antitubulin activity of the target **CA-4** analogues, achieved by replacement of the olefinic bond by triazinone ring that promotes a cis-locked confirmation and enhances the tubulin binding affinity of compounds **6** and **12**. In addition, compounds **6** and **12** blocks the G2/M phase at the cell cycle and induce cell apoptosis in MDA-MB-231. Moreover, these compounds were found to induce apoptosis *via* activation of p53, Bax, and caspase 3/7 as well as downregulation of Bcl-2. Stunningly, enhancement of the pharmacokinetics, particularly water solubility, of the most active compounds, was obtained by loading compound **6** on PEG-PCL modified diamond nanoparticles (PEG-PCL-NDs) as a nano panel for oral delivery. Eight formulae were successfully fabricated *via* 2^3^ full factorial designs. F4 exhibited the highest E.E%, Smaller PS, PDI, and absolute value of ZP. The superiority of F4 relative to six suspensions was confirmed *via* an *in-vitro* drug release study. Interestingly, F4 exhibited outstanding solubility and boosted cytotoxic activity towards MDA-MB-231. Accordingly, PEG-PCL modified NDs can be deployed as a potential panel for the lead compound **6** with boosted solubility and anticancer activity.

## 4. Materials and methods

### 4.1. Chemistry

#### 4.1.1. General

The melting points of the prepared compounds were recorded with a digital melting point apparatus and are uncorrected. The IR spectra were recorded on Shimadzu IR spectrophotometer, Faculty of Pharmacy, Cairo University, Egypt. ^1^H-NMR and ^13^C-NMR spectra in DMSO-d_6_ were respectively recorded at 400 and 100 MHz with Bruker NMR spectrometer (Bruker comp., MA, USA) at Applied Nucleic Acid Research Centre (ANARC), Faculty of Science, Zagazig University, Egypt. The elemental analyses were performed on VARIO EL-III elemental analyser at Mycology and Biotechnology Centre, Al-Azhar University, Egypt. All chemicals and reagents were purchased from *Aldrich* chemical company and were used without further purification.

#### 4.1.2. Synthesis of 4-(3,4,5-trimethoxybenzylidene)-2-(3,4,5-trimethoxyphenyl)oxazol-5(4H)-one (1)

The 4-(3,4,5-trimethoxybenzylidene)-2-(3,4,5-trimethoxyphenyl)oxazol-5(4*H*)-one was prepared according to the literature procedure^36^.

#### 4.1.3. General method for synthesis of N-(3-(2-(aroyl)hydrazinyl)-3-oxo-1-(3,4,5-trimethoxyphenyl)prop-1-en-2-yl)-3,4,5-trimethoxybenzamides 2a–c

To a stirred suspension of oxazolone **1** (2.15 g, 5 mmol) in absolute ethanol (25 ml), the appropriate aryl carboxylic acid hydrazide (5 mmol) was added and the reaction mixture was heated under reflux for 7–8 h. After cooling to room temperature, the precipitated solid was filtered, dried and crystallised from absolute ethanol to afford pure compound **2a–c**.

##### 4.1.3.1. N-(3-(2-(3-hydroxybenzoyl)hydrazinyl)-3-oxo-1-(3,4,5-trimethoxyphenyl)prop-1-en-2-yl)-3,4,5-trimethoxybenzamide (2a)

Pale yellow crystals (2.15 g, 73.81%), m.p. 241–243 °C; IR (KBr, *ν*_max_ cm^−1^): 3278, 3209 (NH), 3071 (arom.CH), 2941, 2843 (aliph.CH), 1667, 1638 (2C = O), 1598, 1489 (C = C), 1232, 1116 (C–O). ^1^H-NMR (400 MHz, DMSO‑d_6_, *δ* ppm): 3.64 (s, 6H, 2OCH_3_), 3.66 (s, 3H, OCH_3_), 3.73 (s, 3H, OCH_3_), 3.83 (s, 6H, 2OCH_3_), 6.95 (dd, *J* = 7.9, 1.5 Hz, 1H, arom. CH), 7.00 (s, 2H, arom. CH), 7.25–7.31 (m, 2H, arom. CH), 7.34 (d, *J* = 7.7 Hz, 1H, arom. CH), 7.37 (s, 1H, olefinic CH), 7.42 (s, 2H, arom. CH), 9.70 (s, 1H, OH), 9.93 (s, 1H, NH), 10.17 (s, 1H, NH), 10.34 (s, 1H, NH) ppm. ^13^C-NMR (100 MHz, DMSO‑d_6_, *δ* ppm): 56.0 (2OCH_3_), 56.6 (2OCH_3_), 60.5 (OCH_3_), 60.6 (OCH_3_), 106.1 (C2,6 trimethoxyphenyl), 107.7 (C2,6 trimethoxybenzamide), 115.0 (C2 hydroxyphenyl), 118.4 (C4 hydroxyphenyl), 119.1 (C6 hydroxyphenyl), 128.5 (C olefinic), 129.2 (C1 trimethoxyphenyl), 129.7 (C olefinic), 129.9 (C1 trimethoxybenzamide), 131.3 (C5 hydroxyphenyl), 134.6 (C4 trimethoxyphenyl), 138.6 (C1 hydroxyphenyl), 140.9 (C4 trimethoxybenzamide), 153.0 (C3,5 trimethoxyphenyl), 153.1 (C3,5 trimethoxybenzamide), 157.8 (C3 hydroxyphenyl), 165.0 (C = O hydrazide), 165.8 (C = O), 166.1 (C = O trimethoxybenzamide). Anal. Calcd. for C_29_H_31_N_3_O_10_ (581.57): C, 59.89; H, 5.37; N, 7.23. Found: C, 59.77; H, 5.17; N, 7.32.

##### 4.1.3.2. N-(3-(2-(4-bromobenzoyl)hydrazinyl)-3-oxo-1-(3,4,5-trimethoxyphenyl)prop-1-en-2-yl)-3,4,5-trimethoxybenzamide (2b)

White crystals (2.33 g, 72.19%), m.p. 236–238 °C; IR (KBr, *ν*_max_ cm^−1^): 3232, 3205 (NH), 3075, 3001 (arom.CH), 2936, 2837 (aliph.CH), 1655, 1639 (2C = O), 1579, 1503 (C = C), 1259, 1123 (C–O). ^1^H-NMR (400 MHz, DMSO‑d_6_, *δ* ppm): 3.65 (s, 6H, 2OCH_3_), 3.67 (s, 3H, OCH_3_), 3.73 (s, 3H, OCH_3_), 3.84 (s, 6H, 2OCH_3_), 7.01 (s, 2H, arom. CH), 7.37 (s, 1H, olefinic CH), 7.43 (s, 2H, arom. CH), 7.74 (d, *J* = 8.6 Hz, 2H, arom. CH), 7.87 (d, *J* = 8.6 Hz, 2H, arom. CH), 9.96 (s, 1H, NH), 10.24 (s, 1H, NH), 10.57 (s, 1H, NH). ^13^C-NMR (100 MHz, DMSO‑d_6_, *δ* ppm): 56.1 (2OCH_3_), 56.6 (2OCH_3_), 60.5 (OCH_3_), 60.7 (OCH_3_), 106.1 (C2,6 trimethoxyphenyl), 107.7 (C2,6 trimethoxybenzamide), 126.0 (C1 trimethoxyphenyl), 128.4 (C olefinic), 129.2 (C olefinic), 129.7 (C1 trimethoxybenzamide), 130.1 (C2,6 bromophenyl), 131.3 (C4 bromophenyl), 132.0 (C3,5 bromophenyl), 132.3 (C1 bromophenyl), 138.7 (C4 trimethoxyphenyl), 140.9 (C4 trimethoxybenzamide), 153.0 (C3,5 trimethoxyphenyl), 153.1 (C3,5 trimethoxybenzamide), 165.0 (C = O hydrazide), 165.1 (C = O), 165.9 (C = O trimethoxybenzamide). Anal. Calcd. for C_29_H_30_BrN_3_O_9_ (644.47): C, 54.05; H, 4.69; N, 6.52. Found: C, 53.88; H, 4.81; N, 6.69.

##### 4.1.3.3. N-(3-(2-isonicotinoylhydrazinyl)-3-oxo-1-(3,4,5-trimethoxyphenyl)prop-1-en-2-yl)-3,4,5-trimethoxybenzamide (2c)

White powder (1.93 g, 68.24%), m.p. 227–229 °C; IR (KBr, *ν*_max_ cm^−1^): 3237, 3193 (NH), 3055 (arom.CH), 2939 (aliph.CH), 1689, 1637 (2C = O), 1605, 1587 (C = C), 1211, 1139 (C–O). ^1^H-NMR (400 MHz, DMSO‑d_6_, *δ* ppm): 3.64 (s, 6H, 2OCH_3_), 3.66 (s, 3H, OCH_3_), 3.73 (s, 3H, OCH_3_), 3.83 (s, 6H, 2OCH_3_), 7.01 (s, 2H, arom. CH), 7.37 (s, 1H, olefinic CH), 7.42 (s, 2H, arom. CH), 7.75–7.90 (m, 2H, arom. CH), 8.77 (d, *J* = 6.0 Hz, 2H, arom. CH), 9.97 (s, 1H, NH), 10.32 (s, 1H, NH), 10.79 (s, 1H, NH). ^13^C-NMR (100 MHz, DMSO‑d_6_, *δ* ppm): 56.1 (2OCH_3_), 56.6 (2OCH_3_), 60.5 (OCH_3_), 60.7 (OCH_3_), 106.0 (C2,6 trimethoxyphenyl), 107.7 (C2,6 trimethoxybenzamide), 121.8 (C2,6 pyridine), 128.4 (C olefinic), 129.2 (C1 trimethoxyphenyl), 129.6 (C olefinic), 131.1 (C1 trimethoxybenzamide), 131.4 (C4 trimethoxyphenyl), 138.7 (C1 pyridine), 140.9 (C4 trimethoxybenzamide), 150.9 (C3,5 pyridine), 153.0 (C3,5 trimethoxyphenyl), 153.1 (C3,5 trimethoxybenzamide), 163.4 (C = O hydrazide), 164.5 (C = O), 165.9 (C = O trimethoxybenzamide). Anal. Calcd. for C_28_H_30_N_4_O_9_ (566.56): C, 59.36; H, 5.34; N, 9.89. Found: C, 59.53; H, 5.12; N, 9.67.

#### 4.1.4. General method for synthesis of 3,4,5-trimethoxy-N-(3-oxo-3-(2-(phenylcarbamothioyl)hydrazinyl)-1-(3,4,5-trimethoxyphenyl)prop-1-en-2-yl)benzamide (3)

A mixture of oxazolone (2.15 g, 5 mmol) and 4-phenylthiosemicarbazide (0.84 g, 5 mmol) in absolute ethanol (25 ml) was heated under reflux for 8 h. The reaction mixture was cooled and the solvent was evaporated under reduced pressure; the obtained solid residue was triturated with water, filtered, and dried. The solid obtained was dried and crystallised from ethanol/H_2_O (3:1) to afford pure compound **3**.

White powder (1.83 g, 61.38%), m.p. 212–214 °C; IR (KBr, *ν*_max_ cm^−1^): 3319, 3246, 3214 (NH), 3065 (arom.CH), 2989, 2969 (aliph.CH), 1671, 1639 (2C = O), 1584, 1544 (C = C), 1329 (C = S), 1123 (C–O). ^1^H-NMR (400 MHz, DMSO‑d_6_, *δ* ppm): 3.69 (s, 3H, OCH_3_), 3.70 (s, 6H, 2OCH_3_), 3.74 (s, 3H, OCH_3_), 3.78 (s, 6H, 2OCH_3_), 7.04 (s, 2H, arom. CH), 7.09–7.21 (m, 2H, arom. CH and olefinic CH), 7.37 (t, *J* = 7.8 Hz, 2H, arom. CH), 7.45 (s, 2H, arom. CH), 7.83 (d, *J* = 7.9 Hz, 2H, arom. CH), 9.31 (s, 1H, NH), 9.91 (s, 1H, NH), 10.57 (s, 2H, 2NH). ^13^C-NMR (100 MHz, DMSO‑d_6_, *δ* ppm): 56.2 (2OCH_3_), 56.5 (2OCH_3_), 60.6 (OCH_3_), 60.7 (OCH_3_), 106.1 (C2,6 trimethoxyphenyl), 108.1 (C2,6 trimethoxybenzamide), 124.0 (C olefinic), 125.1 (C2 phenyl), 126.4 (C1 trimethoxyphenyl), 128.1 (C6 phenyl), 128.5 (C olefinic), 128.6 (C3,5 phenyl), 129.3 (C1 trimethoxybenzamide), 130.4 (C4 phenyl), 138.9 (C4 trimethoxyphenyl), 139.6 (C1 phenyl), 141.4 (C4 trimethoxybenzamide), 153.1 (C3,5 trimethoxyphenyl), 153.1 (C3,5 trimethoxybenzamide), 165.0 (C = O), 167.4 (C = O trimethoxybenzamide), 180.3 (C = S). Anal. Calcd. for C_29_H_32_N_4_O_8_S (596.65): C, 58.38; H, 5.41; N, 9.39. Found: C, 58.27; H, 5.27; N, 9.44.

#### 4.1.5. General method for synthesis of 4-(3,4,5-trimethoxybenzylidene)-2-(3,4,5-trimethoxyphenyl)-1H-imidazol-5(4H)-one (4)

To a stirred suspension of oxazolone **1** (1.29 g, 3 mmol) in 25 ml absolute ethanol, ammonium hydroxide (15 ml) was added. The reaction mixture was heated under reflux and the reaction was monitored by TLC. After completion of the reaction in 24 h, the solvent was removed under reduced pressure, cooled and the obtained solid residue was filtered off, dried, and crystallised from absolute ethanol to afford pure compound **4**.

Pale yellow crystals (0.81 g, 63.28%), m.p. 212–214 °C; IR (KBr, *ν*_max_ cm^−1^): 3398 (NH), 3076 (arom.CH), 2959 (aliph.CH), 1709 (C = O), 1605, 1588 (C = C), 1232, 1193 (C–O). ^1^H-NMR (400 MHz, DMSO‑d_6_, *δ* ppm): 3.63 (s, 9H, 3OCH_3_), 3.69 (s, 3H, OCH_3_), 3.79 (s, 6H, 2OCH_3_), 7.03 (s, 2H, arom. CH), 7.33 (s, 2H, arom. CH), 7.41 (s, 1H, olefinic CH), 9.85 (s, 1H, NH). ^13^C-NMR (100 MHz, DMSO‑d_6_, *δ* ppm): 56.1 (2OCH_3_), 56.5 (2OCH_3_), 60.6 (OCH_3_), 60.6 (OCH_3_), 105.6 (C2,6 trimethoxybenzylidene), 108.0 (C2,6 trimethoxyphenyl), 127.0 (C olefinic), 128.9 (C1 trimethoxyphenyl), 129.5 (C1 trimethoxybenzylidene), 133.7 (C4 imidazolone), 139.0 (C4 trimethoxybenzylidene), 140.9 (C4 trimethoxyphenyl), 153.1 (C3,5 trimethoxybenzylidene), 153.1 (C3,5 trimethoxyphenyl), 165.6 (C2 imidazolone), 166.9 (C = O imidazolone). Anal. Calcd. for C_22_H_24_N_2_O_7_ (428.44): C, 61.67; H, 5.65; N, 6.54. Found: C, 61.94; H, 5.58; N, 6.67.

#### 4.1.6. General method for synthesis of 1-amino-4-(3,4,5-trimethoxybenzylidene)-2-(3,4,5-trimethoxyphenyl)-1H-imidazol-5(4H)-one (5)

To a stirred suspension of oxazolone **1** (1.29 g, 3 mmol) in 25 ml absolute ethanol containing a few drops of glacial acetic acid (8–10 drops), hydrazine hydrate (1 ml) was added. The reaction mixture refluxed and the reaction progress was monitored by TLC. After completion of the reaction in 6 h, the excess solvent was removed under reduced pressure; the obtained solid residue was filtered off, dried, and crystallised from absolute ethanol to afford pure compound **5**.

Pale yellow crystals (1.31 g, 59.22%), m.p. 205–207 °C; IR (KBr, *ν*_max_ cm^−1^): 3449, 3373, 2294 (NH, NH_2_), 3012 (arom.CH), 2946 (aliph.CH), 1710 (C = O), 1593, 1577 (C = C), 1209, 1183 (C–O). ^1^H-NMR (400 MHz, DMSO‑d_6_, *δ* ppm): 3.75 (s, 3H, OCH_3_), 3.78 (s, 3H, OCH_3_), 3.85 (s, 6H, 2OCH_3_), 3.87 (s, 6H, 2OCH_3_), 5.46 (s, 2H, NH_2_, D_2_O exchange), 7.12 (s, 1H, olefinic CH), 7.84 (s, 2H, arom. CH), 7.91 (s, 2H, arom. CH). ^13^C-NMR (100 MHz, DMSO‑d_6_, *δ* ppm): 56.1 (2OCH_3_), 56.3 (2OCH_3_), 60.7 (OCH_3_), 60.7 (OCH_3_), 107.4 (C2,6 trimethoxybenzylidene), 110.3 (C2,6 trimethoxyphenyl), 123.9 (C olefinic), 126.9 (C1 trimethoxyphenyl), 130.3 (C1 trimethoxybenzylidene), 136.7 (C4 imidazolone), 140.0 (C4 trimethoxybenzylidene), 141.3 (C4 trimethoxyphenyl), 152.9 (C3,5 trimethoxybenzylidene), 153.2 (C3,5 trimethoxyphenyl), 159.7 (C2 imidazolone), 170.6 (C = O imidazolone). Anal. Calcd. for C_22_H_25_N_3_O_7_ (443.45): C, 59.59; H, 5.68; N, 9.48. Found: C, 59.41; H, 5.79; N, 9.23.

#### 4.1.7. General method for synthesis of 2-phenyl-5-(3,4,5-trimethoxybenzylidene)-3-(3,4,5-trimethoxyphenyl)-1,2-dihydro-1,2,4-triazin-6(5H)-one (6)

A mixture of oxazolone **1** (2.03 g, 5 mmol) and phenyl hydrazine (0.50 ml, 5 mmol) in 25 ml absolute ethanol was refluxed for 6 h. The reaction mixture was concentrated under reduced pressure and the separated solid was filtered off, dried, and crystallised from absolute ethanol to afford pure compound **6**.

Yellow crystals (4.07 g, 78.29%), m.p. 225–227 °C; IR (KBr, *ν*_max_ cm^−1^): 3325 (NH), 3068 (arom.CH), 2951, 2842 (aliph.CH), 1702 (C = O), 1573, 1492 (C = C), 1125, 1062 (C–O). ^1^H-NMR (400 MHz, DMSO‑d_6_, *δ* ppm): 3.66 (s, 6H, 2OCH_3_), 3.73 (s, 3H, OCH_3_), 3.77 (s, 3H, OCH_3_), 3.89 (s, 6H, 2OCH_3_), 6.72 (d, *J* = 8.4 Hz, 2H, arom. CH), 6.84 (t, *J* = 7.2 Hz, 1H, arom. CH), 7.18–7.30 (m, 3H, arom. Ch and olefinic CH), 7.58 (s, 2H, arom. CH), 7.90 (s, 2H, arom. CH), 9.12 (s, 1H, NH). ^13^C-NMR (100 MHz, DMSO‑d_6_, *δ* ppm): 56.0 (2OCH_3_), 56.2 (2OCH_3_), 60.7 (OCH_3_), 60.7 (OCH_3_), 106.4 (C2,6 trimethoxyphenyl), 110.6 (C2,6 trimethoxybenzylidene), 112.5 (C2,6 phenyl), 120.5 (C olefinic), 122.9 (C4 phenyl), 128.2 (C1 trimethoxyphenyl), 129.9 (C3,5 phenyl), 130.0 (C1 trimethoxybenzylidene), 135.7 (C4 triazinone), 140.3 (C1 phenyl), 141.5 (C4 trimethoxybenzylidene), 146.8 (C4 trimethoxyphenyl), 153.1 (C3,5 trimethoxybenzylidene), 153.2 (C3,5 trimethoxyphenyl), 159.5 (C3 triazinone), 169.7 (C = O triazinone). Anal. Calcd. for C_28_H_29_N_3_O_7_ (519.55): C, 64.73; H, 5.63; N, 8.09. Found: C, 65.03; H, 5.70; N, 7.93.

#### 4.1.8. General method for synthesis of 3-(6-oxo-5–(3,4,5-trimethoxybenzylidene)-3-(3,4,5-trimethoxyphenyl)-1,2,5,6-tetrahydro-1,2,4-triazine-2-carbonyl)phenyl acetate (7)

A mixture of oxazolone **1** (2.01 g, 5 mmol) and 3-hydroxybenzoic acid hydrazide (0.76 g, 5 mmol) in 25 ml acetic anhydride was refluxed for 8 h. Upon pouring on crushed ice, the obtained solid residue was filtered, washed with water, and crystallised from ethanol/H_2_O (1:1) to get pure compound **7**.

Yellow crystals (4.02 g, 66.34%), m.p. 199–201 °C; IR (KBr, *ν*_max_ cm^−1^): 3457 (NH), 3003 (arom.CH), 2968, 2840 (aliph.CH), 1736 (C = O ester), 1668, 1619 (C = O), 1574, 1432 (C = C), 1246, 1127 (C–O). ^1^H-NMR (400 MHz, DMSO‑d_6_, *δ* ppm): 1.90 (s, 3H, CH_3_), 3.73 (s, 3H, OCH_3_), 3.77 (s, 3H, OCH_3_), 3.78 (s, 6H, 2OCH_3_), 3.88 (s, 6H, 2OCH_3_), 7.04 (dt, *J* = 7.6, 1.8 Hz, 1H, arom. CH), 7.25 (s, 1H, olefinic CH), 7.32–7.35 (m, 2H, arom. CH), 7.39 (d, *J* = 4.3 Hz, 1H, arom. CH), 7.44 (s, 2H, arom. CH), 7.88 (s, 2H, arom. CH), 11.36 (s, 1H, NH). ^13^C-NMR (100 MHz, DMSO‑d_6_, *δ* ppm): 21.7 (CH_3_), 56.2 (2OCH_3_), 56.4 (2OCH_3_), 60.7 (2OCH_3_), 105.9 (C2,6 trimethoxyphenyl), 110.6 (C2,6 trimethoxybenzamide), 114.8 (C olefinic), 118.3 (C2 acetoxyphenyl), 120.3 (C6 acetoxyphenyl), 122.8 (C1 trimethoxyphenyl), 128.7 (C4 acetoxyphenyl), 129.8 (C1 trimethoxybenzamide), 130.5 (C5 acetoxyphenyl), 132.5 (C1 acetoxyphenyl), 135.6 (C5 triazine), 140.4 (C4 trimethoxybenzamide), 141.5 (C4 trimethoxyphenyl), 153.2 (C3,5 trimethoxybenzamide), 153.3 (C3,5 trimethoxyphenyl), 158.2 (C3 acetoxyphenyl), 159.5 (C3 triazine), 165.5 (C = O triazinone), 169.0 (C = O acetyl), 172.6 (C = O). Anal. Calcd. for C_31_H_31_N_3_O_10_ (605.59): C, 61.48; H, 5.16; N, 6.94. Found: C, 61.61; H, 5.01; N, 6.79.

#### 4.1.9. General method for synthesis of N-(3-(2-carbamothioylhydrazinyl)-3-oxo-1-(3,4,5-trimethoxyphenyl)prop-1-en-2-yl)-3,4,5-trimethoxybenzamide (8)

A mixture of oxazolone (2.15 g, 5 mmol) and thiosemicarbazide (0.55 g, 6 mmol) was heated under reflux in 30 ml absolute ethanol containing 10 drops of glacial acetic acid for 8 h. The excess solvent was removed under reduced pressure and the obtained solid residue was washed with water, filtered, and crystallised from ethanol/H_2_O (1:1) to afford compound **8**.

White crystals (2.11 g, 81.16%), m.p. 228–230 °C; IR (KBr, *ν*_max_ cm^−1^): 3380, 3278, 3196 (NH), 3074 (arom.CH), 2997, 2833 (aliph.CH), 1667, 1639 (C = O), 1579, 1504 (C = C), 1330 (C = S), 1223, 1091 (C–O). ^1^H-NMR (400 MHz, DMSO‑d_6_, *δ* ppm): 3.65 (s, 6H, 2OCH_3_), 3.67 (s, 3H, OCH_3_), 3.74 (s, 3H, OCH_3_), 3.84 (s, 6H, 2OCH_3_), 6.99 (s, 2H, arom. CH), 7.08 (s, 1H, NH), 7.15 (s, 1H, olefinic CH), 7.40 (s, 2H, arom. CH), 8.06 (s, 1H, NH), 9.49 (s, 1H, NH), 10.25, 10.35 (s, 2H, NH_2_). ^13^C-NMR (100 MHz, DMSO‑d_6_, *δ* ppm): 56.1 (2OCH_3_), 56.6 (2OCH_3_), 60.6 (OCH_3_), 60.7 (OCH_3_), 106.1 (C2,6 trimethoxyphenyl), 107.9 (C2,6 trimethoxybenzamide), 128.0 (C olefinic), 128.8 (C olefinic), 129.4 (C1 trimethoxyphenyl and C1 trimethoxybenzamide), 138.8 (C4 trimethoxyphenyl), 141.2 (C4 trimethoxybenzamide), 153.0 (C3,5 trimethoxyphenyl), 153.1 (C3,5 trimethoxybenzamide), 164.7 (C = O), 167.06 (C = O trimethoxybenzamide), 182.5 (C = S). Anal. Calcd. for C_23_H_28_N_4_O_8_S (520.56): C, 53.07; H, 5.42; N, 10.76. Found: C, 52.73; H, 5.58; N, 10.50.

#### 4.1.10. General method for synthesis of 6-oxo-5-(3,4,5-trimethoxybenzylidene)-3-(3,4,5-trimethoxyphenyl)-5,6-dihydro-1,2,4-triazine-2(1H)-carbothioamide (9)

A mixture of oxazolone (2.15 g, 5 mmol) and thiosemicarbazide (0.55 g, 6 mmol) in 30 ml glacial acetic acid containing (0.49 g, 6 mmol) anhydrous sodium acetate was refluxed for 8 h. Upon pouring on crushed ice, the obtained solid residue was filtered, washed with water, and crystallised from ethanol/H_2_O (3:1) to get pure compound **9**.

Yellow crystals (1.72 g, 68.49%), m.p. 239–241 °C; IR (KBr, *ν*_max_ cm^−1^): 3386, 3303, 3203 (NH, NH_2_), 3068 (arom.CH), 2939, 2837 (aliph.CH), 1699 (C = O), 1616, 1573 (C = C), 1327 (C = S), 1116 (C–O). ^1^H-NMR (400 MHz, DMSO‑d_6_, *δ* ppm): 3.76 (s, 3H, OCH_3_), 3.78 (s, 3H, OCH_3_), 3.87 (s, 12H, 4OCH_3_), 7.18 (s, 1H, olefinic CH), 7.39 (s, 2H, arom. CH), 7.85 (s, 2H, arom. CH), 8.41 (d, *J* = 69.0 Hz, 2H, NH_2_, D_2_O exchange), 10.23 (s, 1H, NH, D_2_O exchange). ^13^C-NMR (100 MHz, DMSO‑d_6_, *δ* ppm): 56.1 (OCH_3_), 56.30 (2OCH_3_), 60.7 (2OCH_3_), 60.8 (OCH_3_), 105.7 (C2,6 trimethoxyphenyl), 110.4 (C2,6 trimethoxybenzylidene), 122.8 (C olefinic), 127.9 (C1 trimethoxyphenyl), 130.0 (C1 trimethoxybenzylidene), 135.8 (C5 triazinone), 140.2 (C4 trimethoxybenzylidene), 141.4 (C4 trimethoxyphenyl), 153.2 (C3,5 trimethoxybenzylidene), 153.3 (C3,5 trimethoxyphenyl), 157.5 (C3 triazinone), 168.7 (C = O triazinone), 182.4 (C = S). Anal. Calcd. for C_23_H_26_N_4_O_7_S (502.54): C, 54.97; H, 5.21; N, 11.15. Found: C, 54.90; H, 5.53; N, 10.93.

#### 4.1.11. General method for synthesis of ethyl 2-(2-(2-(2-(3,4,5-trimethoxybenzamido)-3-(3,4,5-trimethoxyphenyl)acryloyl)hydrazinyl)thiazol-4-yl)acetate (10)

A mixture of hydrazine carbothioamide **8** (0.52 g, 1 mmol), anhydrous sodium acetate (0.12 g, 1.5 mmol), and ethyl 4-chloroacetoacetate (0.14 ml, 1 mmol) in absolute ethanol (20 ml) was heated under reflux for 6 h. The reaction mixture was concentrated under reduced pressure, cooled to room temperature and the solid residue that obtained was filtered and washed with cold ethanol (5 ml) to give pure compound **10**.

White crystals (0.31 g, 49.07%), m.p. 210–212 °C; IR (KBr, *ν*_max_ cm^−1^): 3427, 3309, 3290 (N3H), 3084 (arom.CH), 2941, 2871 (aliph.CH), 1720 (C = O), 1670, 1637 (2 C = O), 1591, 1479 (C = C), 1291, 1223, 1118 (C–O). ^1^H-NMR (400 MHz, DMSO‑d_6_, *δ* ppm): 1.20 (t, *J* = 7.1 Hz, 3H, CH_3_), 3.54 (s, 2H, CH_2_), 3.65 (s, 6H, 2OCH_3_), 3.67 (s, 3H, OCH_3_), 3.73 (s, 3H, OCH_3_), 3.84 (s, 6H, 2OCH_3_), 4.09 (q, *J* = 7.1 Hz, 2H, OCH_2_), 6.57 (s, 1H, olefinic CH), 7.01 (s, 2H, arom. CH), 7.28 (s, 1H, olefinic CH), 7.42 (s, 2H, arom. CH), 9.41 (s, 1H, NH), 9.96 (s, 1H, NH), 10.50 (s, 1H, NH). ^13^C-NMR (100 MHz, DMSO‑d_6_, *δ* ppm): 14.6 (CH_3_), 37.53 (CH_2_), 56.1 (2OCH_3_), 56.6 (2OCH_3_), 60.5 (OCH_3_), 60.7 (OCH_3_), 60.7 (OCH_2_), 105.5 (C olefinic), 106.1 (C2,6 trimethoxyphenyl), 107.8 (C2,6 trimethoxybenzamide), 128.5 (C olefinic), 129.1 (C1 trimethoxyphenyl), 129.6 (C olefinic), 131.0 (C1 trimethoxybenzamide), 138.7 (C4 trimethoxyphenyl), 140.9 (C4 trimethoxybenzamide), 145.6 (C2 thiazole), 153.0 (C3,5 trimethoxyphenyl), 153.1 (C3,5 trimethoxybenzamide), 165.7 (C4 thiazole), 165.8 (C = O hydrazide), 170.5 (C = O trimethoxybenzamide), 173.1 (C = O ester). Anal. Calcd. for C_29_H_34_N_4_O_10_S (630.67): C, 55.23; H, 5.43; N, 8.88. Found: C, 55.38; H, 5.62; N, 8.63.

#### 4.1.12. General method for synthesis of 3,4,5-trimethoxy-N-(3-oxo-3-(2-(4-arylthiazol-2-yl)hydrazinyl)-1-(3,4,5-trimethoxyphenyl)prop-1-en-2-yl)benzamides 11a–e

A suspension of compound **8** (0.52 g, 1 mmol) and appropriate phenacyl bromide (1 mmol) in 20 ml absolute ethanol containing anhydrous sodium acetate (0.16 g, 2 mmol) was refluxed for 2–3 h. After reaction completion, the mixture was allowed to reach ambient room temperature and the resulting solid was collected, dried, and crystallised from ethanol/H_2_O (3:1) to get pure compound **11a-e**.

##### 4.1.12.1. 3,4,5-Trimethoxy-N-(3-oxo-3-(2-(4-arylthiazol-2-yl)hydrazinyl)-1-(3,4,5-trimethoxyphenyl)prop-1-en-2-yl)benzamide (11a)

White crystals (0.44 g, 70.27%), m.p. 212–214 °C; IR (KBr, *ν*_max_ cm^−1^): 3451, 3307, 3216 (NH), 3088 (arom.CH), 2995, 2933 (aliph.CH), 1668, 1638 (2 C = O), 1580, 1470 (C = C), 1292, 1231, 1125 (C–O). ^1^H-NMR (400 MHz, DMSO‑d_6_, *δ* ppm): 3.64 (s, 6H, 2OCH_3_), 3.66 (d, 3H, OCH_3_), 3.73 (s, 3H, OCH_3_), 3.84 (s, 6H, 2OCH_3_), 7.01 (s, 2H, arom. CH), 7.24 (s, 1H, olefinic CH), 7.27–7.34 (m, 2H, arom. CH and thiazole CH), 7.39 (t, *J* = 7.6 Hz, 2H, arom. CH), 7.43 (s, 2H, arom. CH), 7.84 (d, *J* = 7.3 Hz, 2H, arom. CH), 9.57 (s, 1H, NH), 9.97 (s, 1H, NH), 10.57 (s, 1H, NH). ^13^C-NMR (100 MHz, DMSO‑d_6_, *δ* ppm): 56.1 (2OCH_3_), 56.57 (2OCH_3_), 60.6 (OCH_3_), 60.7 (OCH_3_), 103.6 (C5 thiazole), 106.1 (C2,6 trimethoxyphenyl), 107.8 (C2,6 trimethoxybenzamide), 126.0 (C2,6 phenyl), 127.9 (C olefinic), 128.5 (C1 trimethoxyphenyl), 129.0 (C3,5 phenyl), 129.1 (C olefinic), 129.6 (C1 trimethoxybenzamide), 131.1 (C4 phenyl), 135.2 (C1 phenyl), 138.7 (C4 trimethoxyphenyl), 140.9 (C4 trimethoxybenzamide), 151.0 (C4 thiazole), 153.0 (C3,5 trimethoxyphenyl), 153.1 (C3,5 trimethoxybenzamide), 165.8 (C = O hydrazide), 165.8 (C = O trimethoxybenzamide), 173.1 (C2 thiazole). Anal. Calcd. for C_31_H_32_N_4_O_8_S (620.67): C, 59.99; H, 5.20; N, 9.03. Found: C, 60.07; H, 4.97; N, 8.91.

##### 4.1.12.2. N-(3-(2-(4-(4-chlorophenyl)thiazol-2-yl)hydrazinyl)-3-oxo-1-(3,4,5-trimethoxyphenyl)prop-1-en-2-yl)-3,4,5-trimethoxybenzamide (11b)

White crystals (0.45 g, 69.37%), m.p. 212–214 °C; IR (KBr, *ν*_max_ cm^−1^): 3417, 3309, 3301 (NH), 3078, 3032 (arom.CH), 2973, 2920 (aliph.CH), 1669, 1635 (2C = O), 1587, 1472 (C = C), 1288, 1230, 1128 (C–O). ^1^H-NMR (400 MHz, DMSO-d_6_, *δ* ppm): 3.65 (s, 6H, 2OCH_3_), 3.67 (s, 3H, OCH_3_), 3.74 (s, 3H, OCH_3_), 3.84 (s, 6H, 2OCH_3_), 7.02 (s, 2H, arom. CH), 7.32 (d, *J* = 5.0 Hz, 2H, arom. CH), 7.43 (s, 2H, arom. CH), 7.44 (s, 1H, olefinic CH), 7.46 (s, 1H, thiazole CH), 7.86 (d, *J* = 8.6 Hz, 2H, arom. CH), 9.62 (s, 1H, NH), 9.98 (s, 1H, NH), 10.59 (s, 1H, NH). ^13^C-NMR (100 MHz, DMSO‑d_6_, *δ* ppm): 56.1 (2OCH_3_), 56.6 (2OCH_3_), 60.6 (OCH_3_), 60.7 (OCH_3_), 104.4 (C5 thiazole), 106.1 (C2,6 trimethoxyphenyl), 107.8 (C2,6 trimethoxybenzamide), 127.7 (C2,6 chlorophenyl), 128.5 (C olefinic), 129.0 (C3,5 chlorophenyl), 129.1 (C1 trimethoxyphenyl), 129.6 (C olefinic), 131.1 (C1 trimethoxybenzamide), 132.3 (C1 chlorophenyl), 134.1 (C4 chlorophenyl), 138.8 (C4 trimethoxyphenyl), 140.9 (C4 trimethoxybenzamide), 149.7 (C4 thiazole), 153.0 (C3,5 trimethoxyphenyl), 153.1 (C3,5 trimethoxybenzamide), 165.8 (C = O hydrazide), 170.7 (C = O trimethoxybenzamide), 173.3 (C2 thiazole). Anal. Calcd. for C_31_H_31_ClN_4_O_8_S (655.12): C, 56.83; H, 4.77; N, 8.55. Found: C, 56.41; H, 5.10; N, 8.67.

##### 4.1.12.3. N-(3-(2-(4-(4-bromophenyl)thiazol-2-yl)hydrazinyl)-3-oxo-1-(3,4,5-trimethoxyphenyl)prop-1-en-2-yl)-3,4,5-trimethoxybenzamide (11c)

White crystals (0.45 g, 63.84%), m.p. 212–214 °C**;** IR (KBr, *ν*_max_ cm^−1^): 3428, 3309, 3232 (NH), 3071 (arom.CH), 2936, 2831 (aliph.CH), 1672, 1634 (2C = O), 1581, 1483 (C = C), 1230, 1118, 1004 (C–O). ^1^H-NMR (400 MHz, DMSO-d_6_, *δ* ppm): 3.65 (s, 6H, 2OCH_3_), 3.66 (s, 3H, OCH_3_), 3.73 (s, 3H, OCH_3_), 3.84 (s, 6H, 2OCH_3_), 7.01 (s, 2H, arom. CH), 7.30 (s, 1H, olefinic CH), 7.31 (s, 1H, thiazole CH), 7.43 (s, 2H, arom. CH), 7.58 (d, *J* = 8.5 Hz, 2H, arom. CH), 7.79 (d, *J* = 8.5 Hz, 2H, arom. CH), 9.60 (s, 1H, NH), 9.97 (s, 1H, NH), 10.57 (s, 1H, NH) ppm. ^13^C-NMR (100 MHz, DMSO‑d_6_, *δ* ppm): 55.6 (2OCH_3_), 56.1 (2OCH_3_), 60.1 (OCH_3_), 60.2 (OCH_3_), 103.9 (C5 thiazole), 105.6 (C2,6 trimethoxyphenyl), 107.3 (C2,6 trimethoxybenzamide), 120.4 (C olefinic), 127.6 (C2,6 bromophenyl), 128.1 (C4 bromophenyl), 128.7 (C1 trimethoxyphenyl), 129.2 (C olefinic), 130.5 (C1 trimethoxybenzamide), 131.5 (C3,5 bromophenyl), 134.0 (C1 bromophenyl), 138.2 (C4 trimethoxyphenyl), 140.5 (C4 trimethoxybenzamide), 149.3 (C4 thiazole), 152.5 (C3,5 trimethoxyphenyl), 152.6 (C3,5 trimethoxybenzamide), 165.2 (C = O trimethoxybenzamide), 165.4 (C = O hydrazide), 172.7 (C2 thiazole). Anal. Calcd. for C_31_H_31_BrN_4_O_8_S (699.57): C, 53.22; H, 4.47; N, 8.01. Found: C, 53.09; H, 4.63; N, 8.18.

##### 4.1.12.4. 3,4,5-Trimethoxy-N-(3-(2-(4-(3-nitrophenyl)thiazol-2-yl)hydrazinyl)-3-oxo-1–(3,4,5-trimethoxyphenyl)prop-1-en-2-yl)benzamide (11d)

White crystals (0.37 g, 55.08%), m.p. 212–214 °C**;** IR (KBr, *ν*_max_ cm^−1^): 3394, 3303, 3219 (NH), 3009 (arom.CH), 2927, 2872 (aliph.CH), 1674, 1633 (2 C = O), 1599, 1473 (C = C), 1581, 1362 (NO_2_), 1239, 1216, 1019 (C–O). ^1^H-NMR (400 MHz, DMSO-d_6_, *δ* ppm): 3.66 (s, 6H, 2OCH_3_), 3.68 (s, 3H, OCH_3_), 3.74 (s, 3H, OCH_3_), 3.85 (s, 6H, 2OCH_3_), 7.03 (s, 2H, arom. CH), 7.31 (s, 1H, olefinic CH), 7.44 (s, 2H, arom. CH), 7.59 (s, 1H, thiazole CH), 7.71 (t, *J* = 8.0 Hz, 1H, arom. CH), 8.15 (d, *J* = 8.2 Hz, 1H, arom. CH), 8.30 (d, *J* = 7.8 Hz, 1H, arom. CH), 8.67 (s, 1H, arom. CH), 9.74 (s, 1H, NH), 10.00 (s, 1H, NH), 10.64 (s, 1H, NH). ^13^C-NMR (100 MHz, DMSO‑d_6_, *δ* ppm): 56.1- (2OCH_3_), 56.6 (2OCH_3_), 60.6 (OCH_3_), 60.7 (OCH_3_), 106.1 (C2,6 trimethoxyphenyl), 106.4 (C5 thiazole), 107.8 (C2,6 trimethoxybenzamide), 120.4 (C5 nitrophenyl), 122.4 (C4 nitrophenyl), 128.5 (C olefinic), 129.1 (C1 trimethoxyphenyl), 129.6 (C olefinic), 130.7 (C1 trimethoxybenzamide), 131.0 (C2 nitrophenyl), 132.1 (C6 nitrophenyl), 136.7 (C1 nitrophenyl), 138.8 (C4 trimethoxyphenyl), 141.0 (C4 trimethoxybenzamide), 148.5 (C3 nitrophenyl), 148.8 (C4 thiazole), 153.0 (C3,5 trimethoxyphenyl), 153.1 (C3,5 trimethoxybenzamide), 165.8 (C = O trimethoxybenzamide), 165.9 (C = O hydrazide), 173.5 (C2 thiazole). Anal. Calcd. for C_31_H_31_N_5_O_10_S (665.67): C, 55.93; H, 4.69; N, 10.52. Found: C, 55.96; H, 4.74; N, 10.48.

##### 4.1.12.5. 3,4,5-Trimethoxy-N-(3-(2-(4-(3-methoxyphenyl)thiazol-2-yl)hydrazinyl)-3-oxo-1–(3,4,5-trimethoxyphenyl)prop-1-en-2-yl)benzamide (11e)

White crystals (0.39 g, 59.85%), m.p. 212–214 °C; IR (KBr, *ν*_max_ cm^−1^): 3422, 3305, 3232 (NH), 3059 (arom.CH), 2934 (aliph.CH), 1671, 1636 (2C = O), 1593, 1481 (C = C), 1234, 1212, 1099 (C–O). ^1^H-NMR (400 MHz, DMSO-d_6_, *δ* ppm): 3.65 (s, 6H, 2OCH_3_), 3.67 (s, 3H, OCH_3_), 3.74 (s, 3H, OCH_3_), 3.80 (s, 3H, OCH_3_), 3.84 (s, 6H, 2OCH_3_), 6.87 (dd, *J* = 8.2, 1.9 Hz, 1H, arom. CH), 7.02 (s, 2H, arom. CH), 7.28 (s, 1H, olefinic CH), 7.30–7.34 (m, 2H), 7.39–7.43 (m, 2H, arom. CH), 7.43 (s, 2H, arom. CH), 9.57 (s, 1H, NH), 9.97 (s, 1H, NH), 10.57 (s, 1H, NH). ^13^C-NMR (100 MHz, DMSO‑d_6_, *δ* ppm): 55.5 (OCH_3_), 56.1 (2OCH_3_), 56.6 (2OCH_3_), 60.6 (OCH_3_), 60.7 (OCH_3_), 104.0 (C5 thiazole), 106.1 (C2,6 trimethoxyphenyl), 107.8 (C2,6 trimethoxybenzamide), 111.4 (C2 methoxyphenyl), 113.6 (C4 methoxyphenyl), 117.9 (C olefinic), 118.4 (C6 methoxyphenyl), 121.9 (C olefinic), 128.0 (C1 trimethoxyphenyl), 129.1 (C1 trimethoxybenzamide), 130.1 (C5 methoxyphenyl), 131.1 (C1 methoxyphenyl), 136.6 (C4 trimethoxyphenyl), 141.0 (C4 trimethoxybenzamide), 150.8 (C4 thiazole), 153.0 (C3,5 trimethoxyphenyl), 153.1 (C3,5 trimethoxybenzamide), 160.0 (C3 methoxyphenyl), 161.6 (C = O trimethoxybenzamide), 165.8 (C = O hydrazide), 173.0 (C2 thiazole). Anal. Calcd. for C_32_H_34_N_4_O_9_S (650.70): C, 59.07; H, 5.27; N, 8.61. Found: C, 58.91; H, 5.07; N, 8.74.

#### 4.1.13. General method for synthesis of ethyl 2-(2-(6-oxo-5-(3,4,5-trimethoxybenzylidene)-3–(3,4,5-trimethoxyphenyl)-5,6-dihydro-1,2,4-triazin-2(1H)-yl)thiazol-4-yl)acetate (12)

A suspension of *N*-thioamide triazinone **9** (0.50 g, 1 mmol), anhydrous sodium acetate (0.12 g, 1.5 mmol), and ethyl 4-chloroacetoacetate (0.14 ml, 1 mmol) in absolute ethanol (20 ml) was heated under reflux for 8 h. The reaction mixture was concentrated, cooled to room temperature and the solid formed was filtered, dried, and crystallised from absolute ethanol to afford compound **12**.

Yellow crystals (0.32 g, 52.39%), m.p. 178–180 °C**;** IR (KBr, *ν*_max_ cm^−1^): 3231 (NH), 3034 (arom.CH), 2937, 2844 (aliph.CH), 1724 (C = O ester), 1679 (C = O), 1601, 1588 (C = C), 1181, 1030 (C-O). ^1^H-NMR (400 MHz, DMSO-d_6_, *δ* ppm): 1.13 (t, *J* = 7.1 Hz, 3H, CH_3_), 3.56 (s, 2H, CH_2_), 3.75 (s, 6H, 2OCH_3_), 3.76 (s, 3H, OCH_3_), 3.77 (s, 3H, OCH_3_), 3.88 (s, 6H, 2OCH_3_), 4.04 (q, *J* = 7.0 Hz, 2H, OCH_2_), 6.74 (s, 1H, olefinic CH), 7.24 (s, 1H, olefinic CH), 7.49 (s, 2H, arom. CH), 7.88 (s, 2H, arom. CH), 10.69 (s, 1H, NH). ^13^C-NMR (100 MHz, DMSO‑d_6_, *δ* ppm): 14.4 (CH_3_), 37.0 (CH_2_), 56.2 (4OCH_3_), 60.7 (2OCH_3_), 60.8 (OCH_2_), 106.1 (C2,6 trimethoxyphenyl), 107.6 (C olefinic), 110.7 (C2,6 trimethoxybenzylidene), 122.7 (C olefinic), 128.8 (C1 trimethoxybenzylidene), 129.8 (C1 trimethoxyphenyl), 135.2 (C5 triazine), 140.5 (C4 trimethoxybenzylidene), 141.5 (C4 trimethoxyphenyl), 153.2 (C3,5 trimethoxybenzylidene), 153.2 (C3,5 trimethoxyphenyl), 153.9 (C4 thiazole), 158.6 (C3 triazine), 168.6 (C2 thiazole), 170.2 (C = O triazine), 170.2 (C = O ester). Anal. Calcd. for C_29_H_32_N_4_O_9_S (612.65): C, 56.85; H, 5.26; N, 9.14. Found: C, 57.03; H, 5.33; N, 9.02.

#### 4.1.14. General method for synthesis of 2-(4-arylthiazol-2-yl)-5-(3,4,5-trimethoxybenzylidene)-3–(3,4,5-trimethoxyphenyl)-1,2-dihydro-1,2,4-triazin-6(5H)-ones 13a–e

A mixture of *N*-thioamide triazinone **9** (0.50 g, 1 mmol), anhydrous sodium acetate (0.16 g, 2 mmol), and appropriate phenacyl bromide (1 mmol) in absolute ethanol (20 ml) was heated under reflux for 3–4 h. After reaction completion, the mixture was cooled to room temperature and concentrated under reduced pressure. The formed solid residue was filtered, washed with cold ethanol (5 ml), and crystallised from ethanol/H_2_O (3:1) to afford pure compound **13a–e**.

##### 4.1.14.1. 2-(4-Phenylthiazol-2-yl)-5-(3,4,5-trimethoxybenzylidene)-3–(3,4,5-trimethoxyphenyl)-1,2-dihydro-1,2,4-triazin-6(5H)-one (13a)

Pale yellow crystals (0.50 g, 83.11%), m.p. 212–214 °C; IR (KBr, *ν*_max_ cm^−1^): 3221 (NH), 3011 (arom.CH), 2952, 2839 (aliph.CH), 1698 (C = O), 1613, 1566 (C = C), 1193, 1032 (C–O). ^1^H-NMR (400 MHz, DMSO‑d_6_, *δ* ppm): 3.73 (s, 3H, OCH_3_), 3.74 (s, 6H, 2OCH_3_), 3.76 (s, 3H, OCH_3_), 3.88 (s, 6H, 2OCH_3_), 7.24 (s, 1H, olefinic CH), 7.26–7.30 (m, 1H thiazole CH), 7.36 (t, *J* = 8.2 Hz, 3, arom. CH), 7.58 (s, 2H, arom. CH), 7.72–7.77 (m, 2H, arom. CH), 7.89 (s, 2H, arom. CH), 10.87 (s, 1H, NH). ^13^C-NMR (100 MHz, DMSO‑d_6_, *δ* ppm): 56.2 (2OCH_3_), 56.2 (2OCH_3_), 60.7 (OCH_3_), 60.7 (OCH_3_), 106.2 (C2,6 trimethoxyphenyl), 110.3 (C5 thiazole), 110.7 (C2,6 trimethoxybenzylidene), 122.8 (C olefinic), 126.1 (C2,6 phenyl), 129.1 (C1 trimethoxyphenyl), 129.8 (C3,5 phenyl), 130.4 (C1 trimethoxybenzylidene), 133.7 (C4 phenyl), 134.5 (C1 phenyl), 136.8 (C5 triazine), 140.5 (C4 trimethoxybenzylidene), 141.5 (C4 trimethoxyphenyl), 150.9 (C4 thiazole), 153.3 (C3,5 trimethoxyphenyl and C3,5 trimethoxybenzylidene), 165.5 (C3 triazine), 168.8 (C = O triazine), 177.1 (C2 thiazole). Anal. Calcd. for C_31_H_30_N_4_O_7_S (602.66): C, 61.78; H, 5.02; N, 9.30. Found: C, 61.83; H, 5.08; N, 9.25.

##### 4.1.14.2. 2-(4-(4-Chlorophenyl)thiazol-2-yl)-5-(3,4,5-trimethoxybenzylidene)-3-(3,4,5-trimethoxyphenyl)-1,2-dihydro-1,2,4-triazin-6(5H)-one (13b)

Yellow crystals (0.51 g, 80.81%), m.p. 212–214 °C**;** IR (KBr, *ν*_max_ cm^−1^): 3288 (NH), 3062 (arom.CH), 2955, 2831 (aliph.CH), 1692 (C = O), 1623, 1534 (C = C), 1208, 1092 (C–O). ^1^H-NMR (400 MHz, DMSO‑d_6_, *δ* ppm): 3.74 (s, 3H, OCH_3_), 3.74 (s, 6H, 2OCH_3_), 3.78 (s, 3H, OCH_3_), 3.89 (s, 6H, 2OCH_3_), 7.27 (s, 1H, olefinic CH), 7.39–7.49 (m, 3H, arom. CH and thiazole CH), 7.56 (s, 2H, arom. CH), 7.77 (d, *J* = 8.6 Hz, 2H, arom. CH), 7.90 (s, 2H, arom. CH), 10.94 (s, 1H, NH). ^13^C-NMR (100 MHz, DMSO‑d_6_, *δ* ppm): 56.2 (2OCH_3_), 56.2 (2OCH_3_), 60.7 (OCH_3_), 60.7 (OCH_3_), 102.5 (C5 thiazole), 106.1 (C2,6 trimethoxyphenyl), 110.7 (C2,6 trimethoxybenzylidene), 122.8 (C olefinic), 127.8 (C2,6 chlorophenyl), 128.9 (C1 trimethoxyphenyl), 129.2 (C3,5 chlorophenyl), 129.8 (C1 trimethoxybenzylidene), 132.7 (C1 chlorophenyl), 133.3 (C4 chlorophenyl), 135.2 (C5 triazine), 140.5 (C4 trimethoxybenzylidene), 141.5 (C4 trimethoxyphenyl), 149.6 (C4 thiazole), 153.3 (C3,5 trimethoxyphenyl and C3,5 trimethoxybenzylidene), 158.6 (C3 triazine), 168.5 (C = O triazine), 168.8 (C2 thiazole). Anal. Calcd. for C_31_H_29_ClN_4_O_7_S (637.10): C, 58.44; H, 4.59; N, 8.79. Found: C, 58.69; H, 4.47; N, 9.09.

##### 4.1.14.3. 2-(4-(4-Bromophenyl)thiazol-2-yl)-5-(3,4,5-trimethoxybenzylidene)-3-(3,4,5-trimethoxyphenyl)-1,2-dihydro-1,2,4-triazin-6(5H)-one (13c)

Yellow powder (0.49 g, 72.23%), m.p. 212–214 °C**;** IR (KBr, *ν*_max_ cm^−1^): 3266 (NH), 3071 (arom.CH), 2941, 2833 (aliph.CH), 1694 (C = O), 1608, 1530 (C = C), 1213, 1055 (C–O).^1^H-NMR (400 MHz, DMSO‑d_6_, *δ* ppm): 3.74 (s, 9H, 3OCH_3_), 3.78 (s, 3H, OCH_3_), 3.89 (s, 6H, 2OCH_3_), 7.27 (s, 1H, olefinic CH), 7.48 (s, 1H, thiazole CH), 7.54 (s, 2H), 7.57 (d, *J* = 8.5 Hz, 2H, arom. CH), 7.71 (d, *J* = 8.5 Hz, 2H, arom. CH), 7.90 (s, 2H, arom. CH), 10.85 (s, 1H, NH). ^13^C-NMR (100 MHz, DMSO‑d_6_, *δ* ppm): 56.2 (2OCH_3_), 56.2 (2OCH_3_), 60.7 (OCH_3_), 60.7 (OCH_3_), 106.1 (C2,6 trimethoxyphenyl), 110.7 (C2,6 trimethoxyphenyl), 113.4 (C5 thiazole), 122.7 (C olefinic), 128.1 (C2,6 bromophenyl), 129.0 (C1 trimethoxyphenyl), 130.0 (C4 bromophenyl), 131.1 (C1 trimethoxybenzylidene), 132.1 (C3,5 bromophenyl), 133.7 (C1 bromophenyl), 135.2 (C5 triazine), 140.5 (C4 trimethoxybenzylidene), 141.6 (C4 trimethoxyphenyl), 145.3 (C4 thiazole), 153.3 (C3,5 trimethoxyphenyl and C3,5 trimethoxybenzylidene), 160.0 (C3 triazine), 166.0 (C = O triazine), 171.7 (C2 thiazole). Anal. Calcd. for C_31_H_29_BrN_4_O_7_S (681.55): C, 54.63; H, 4.29; N, 8.22. Found: C, 54.73; H, 4.42; N, 8.09.

##### 4.1.14.4. 2-(4-(3-Nitrophenyl)thiazol-2-yl)-5-(3,4,5-trimethoxybenzylidene)-3-(3,4,5-trimethoxyphenyl)-1,2-dihydro-1,2,4-triazin-6(5H)-one (13d)

Pale yellow powder (0.54 g, 84.09%), m.p. 212–214 °C; IR (KBr, *ν*_max_ cm^−1^): 3261 (NH), 3097 (arom.CH), 2939, 2833 (aliph.CH), 1703 (C = O), 1625, 1529 (C = C), 1578, 1364 (NO_2_), 1233, 1041 (C–O). ^1^H-NMR (400 MHz, DMSO‑d_6_, *δ* ppm): 3.74 (s, 3H, OCH_3_), 3.75 (s, 6H, 2OCH_3_), 3.78 (s, 3H, OCH_3_), 3.89 (s, 6H, 2OCH_3_), 7.28 (s, 1H, olefinic CH), 7.56 (s, 2H, arom. CH), 7.68–7.73 (m, 2H, thiazole and arom. CH), 7.90 (s, 2H, arom. CH), 8.14 (dd, *J* = 8.2, 1.5 Hz, 1H, arom. CH), 8.21 (d, *J* = 7.8 Hz, 1H, arom. CH), 8.54–8.58 (m, 1H, arom. CH), 10.96 (s, 1H, NH). ^13^C-NMR (100 MHz, DMSO‑d_6_, *δ* ppm): 56.2 (2OCH_3_), 56.2 (2OCH_3_), 60.7 (2OCH_3_), 106.1 (C2,6 trimethoxyphenyl), 108.1 (C5 thiazole), 110.7 (C2,6 trimethoxybenzylidene), 120.4 (C5 nitrophenyl), 122.7 (C4 nitrophenyl), 122.8 (C olefinic), 129.1 (C1 trimethoxyphenyl), 129.8 (C1 trimethoxybenzylidene), 130.8 (C2 nitrophenyl), 132.2 (C6 nitrophenyl), 135.1 (C1 nitrophenyl), 136.0 (C5 triazine), 140.6 (C4 trimethoxybenzylidene), 141.6 (C4 trimethoxyphenyl), 148.5 (C3 nitrophenyl), 148.7 (C4 thiazole), 153.3 (C3,5 trimethoxybenzylidene), 153.3 (C3,5 trimethoxyphenyl), 158.5 (C3 triazine), 165.8 (C = O triazine), 168.8 (C2 thiazole). Anal. Calcd. for C_31_H_29_N_5_O_9_S (647.66): C, 57.49; H, 4.51; N, 10.81. Found: C, 57.72; H, 4.63; N, 11.12.

##### 4.1.14.5. 2-(4-(3-Methoxyphenyl)thiazol-2-yl)-5-(3,4,5-trimethoxybenzylidene)-3-(3,4,5-trimethoxyphenyl)-1,2-dihydro-1,2,4-triazin-6(5H)-one (13e)

Orange powder (0.44 g, 69.52%), m.p. 212–214 °C**;** IR (KBr, *ν*_max_ cm^−1^): 3234 (NH), 3006 (arom.CH), 2928, 2836 (aliph.CH), 1694 (C = O), 1627, 1573 (C = C), 1237, 1129 (C–O). ^1^H-NMR (400 MHz, DMSO‑d_6_, *δ* ppm): 3.74 (s, 3H, OCH_3_), 3.75 (s, 3H, OCH_3_), 3.75 (s, 6H, 2OCH_3_), 3.78 (s, 3H, OCH_3_), 3.89 (s, 6H, 2OCH_3_), 6.90–6.84 (m, 1H, arom. CH), 7.25–7.32 (m, 3H, arom. CH and olefinic CH), 7.34 (d, *J* = 7.7 Hz, 1H, arom. CH), 7.42 (s, 1H, thiazole CH), 7.56 (s, 2H, arom. CH), 7.90 (s, 2H, arom. CH), 10.86 (s, 1H, NH). ^13^C-NMR (100 MHz, DMSO‑d_6_, *δ* ppm): 55.5 (OCH_3_), 56.2 (2OCH_3_), 56.2 (2OCH_3_), 60.7 (2OCH_3_), 105.9 (C5 thiazole), 106.1 (C2,6 trimethoxyphenyl), 110.7 (C2,6 trimethoxybenzylidene), 111.7 (C2 methoxyphenyl), 113.8 (C4 methoxyphenyl), 118.5 (C6 methoxyphenyl), 122.8 (C olefinic), 128.9 (C1 trimethoxyphenyl), 129.8 (C1 trimethoxybenzylidene), 130.2 (C5 methoxyphenyl), 135.2 (C1 methoxyphenyl), 135.8 (C5 triazine), 140.5 (C4 trimethoxybenzylidene), 141.6 (C4 trimethoxyphenyl), 150.7 (C4 thiazole), 153.3 (C3,5 trimethoxyphenyl and C3,5 trimethoxybenzylidene), 158.7 (C3 methoxyphenyl), 159.9 (C3 triazine), 168.0 (C = O triazine), 168.9 (C2 thiazole). Anal. Calcd. for C_32_H_32_N_4_O_8_S (632.68): C, 60.75; H, 5.10; N, 8.86. Found: C, 60.88; H, 5.03; N, 9.07.

### 4.2. Molecular modeling job

Molecular docking was performed using AutoDock vina. The 3D structure of the tubulin complex was retrieved from the Protein Data Bank (PDB ID: 1SA0). The complex with colchicine forming by chain B of the tubulin heterodimer was selected for the modelling job. Ligands were sketched and energy minimised and the protein was prepared using the Discovery studio suite (V5.2).

### 4.3. Biological studies

#### 4.3.1. Cytotoxic activity evaluation

To measure the cytotoxic activity of the synthesised derivatives 2a–13e in breast cancer (MDA-MB-231) cell line. MTT assay method was applied to assess cell viability assay. All experimental steps were conducted as reported earlier^14^.

For more detailed experimental work, see Supplementary Data (part1)

#### 4.3.2. Tubulin inhibitions assay

Triazinone derivatives 6 and 12 were assessed for their tubulin inhibitory activity according to manufacturer’s instructions using # abcam Human Beta-tubulin simple step ELISA Kit ab245722.

For more detailed experimental work, see Supplementary Data (part1)

#### 4.3.3. Cell cycle analysis of compound 6 and 12

Cell cycle analysis in MDA-MB-231 cells was investigated using a fluorescent Annexin V-FITC/PI detection kit (BioVision EZCell™ Cell Cycle Analysis Kit Catalog #K920) by flow cytometry assay. All experimental steps were performed as reported earlier^14^.

For more detailed experimental work, see Supplementary Data (part1)

#### 4.3.4. Apoptosis assay for compounds 6 and 12

Apoptosis in MDA-MB-231 cells was investigated using a fluorescent Annexin V-FITC/PI detection kit (BioVision Annexin V-FITC Apoptosis Detection Kit, Catalog #: K101) by flow cytometry assay. All experimental steps were conducted as reported earlier^14^.

For more detailed experimental work, see Supplementary Data (part1)

#### 4.3.5. Effect on p53, Bax, and Bcl-2

p53, Bax, and Bcl2 enzyme activities in MDA-MB-231 cells were investigated in the presence of compounds 6 and 12 at their IC50 concentration (µM). The levels of the tumour suppressor gene p53, apoptotic markers Bax as well as the anti-apoptotic marker Bcl-2 were assessed using the p53 ELISA kit, Human Bax ELISA kit, and Bcl-2 Elisa kit. The procedure of the used kits was done according to the manufacturer's instructions and was conducted as reported earlier^14^.

For more detailed experimental work, see Supplementary Data (part1)

#### 4.3.6. Caspase 3/7 assay for compounds 6 and 12

Caspase 3/7 in MDA-MB-231 cells was detected using CellEvent^®^ Caspase 3/7 Green Detection Flow Cytometry Assay Kit. Caspase 3/7 activity in the MDA-MB-231 cell line was detected in the presence of compounds **6** and **12** at their IC50 concentration (µM) using CellEvent^®^ caspase 3/7 green detection flow cytometry assay kit according to the manufacturer’s directions. All experimental steps were conducted as reported earlier^14^.

For more detailed experimental work, see Supplementary Data (part1).

### 4.4. Tailoring of compound 6-loaded PEG-PCL modified NDs (6-PEG-PCL modified NDs)

**6-**PEG-PCLmodified NDs were fabricated as follows: first lodging compound **6** on NDs followed by the coating of the outer layer of the NDs by PEG-PCL. Precisely 1 ml of compound **6** solution (20 mg/mL) in methanol was added dropwise into 10 ml of ND dispersion in ethanol containing different concentrations of ND either (1 mg/mL or 4 mg/mL) and different amount of PC either (25 or 50 mg) followed by probe sonication by Branson sonifier (ScientificFisher, Waltham, MA, USA) for 5 min. Then the solvent was evaporated to complete dryness in a rotary evaporator (Heidolph Laborata 4000, Germany) and kept under reduced pressure. The dry film was dispersed with 10 ml PEG-PCL (20 or 60 mg) solution in a rotary evaporator (Heidolph Laborata 4000, Germany) followed by probe sonication for 5 min^49^. The acquired formulae were kept at 4 °C for further characterisation.

### 4.5. *In-vitro* assessment and optimisation of compound 6-loaded PEG-PCL modified NDs

#### 4.5.1. Drug loading efficiency percentage (DL%)

To investigate the loading efficiency of the drug (DL %), HPLC analysis of NDs was conducted twice: before and after cooling centrifugation at 15,000 rpm (Beckman, Fullerton, Canada) for 30 min. The estimation of the total amount of drug and the non-lodged drug was determined using HPLC at 252 nm before and after centrifugation^59^. % DL was computed utilising the following equation:

$$\%\text{DL}={\text{W}}_{\text{total}}-{\text{W}}_{\text{unloaded}}/{\text{W}}_{\text{total}}*100$$

#### 4.5.2. Investigation of zeta potential, vesicle size, and PDI

The mean vesicle size, PDI, and zeta potential of the tailored PEG-PCL modified NDs were adopting the Malvern sizer (Malvern Instruments, Malvern, UK). Before analysis, 0.1 ml of NDs dispersion was diluted with 10 ml distilled water in a glass tube and then convulsed manually for 5 min to acquire the adequate scattering intensity. The test was conducted in triplicate^60^.

#### 4.5.3. Employment of experimental design and selection of optimal 6-loaded PEG-PCL modified NDs

Design Expert^®^ software version 13 (Stat Ease, Inc., Minneapolis, MN, USA) was employed to analyse the influence of various factors in tailoring of PEG-PCL modified NDs which results in formulation of eight runs adopting 2^3^ factorial experiments. The predetermined three factors were: ND concentration (A), PEG-PCL amount (B), and PC amount (C) that were the independent variables, whereas EE% (Y1), PS (Y2), PDI (Y3), and ZP (Y4) were considered as dependent variables. Moreover, based on the EE%, ZP, and minimum PDI, globule size, the optimum (**6**)-loaded PEG-PCL modified formula was elected. In addition, ANOVA was manipulated to highlight the chief influence of the factors under assessment: the prominence of each variable was investigated and optimum formula of the dominant desirability value was selected to be involved in later examinations.

### 4.6. *In-vitro* investigation of the optimum (6)-loaded PEG-PCL modified NDs

#### 4.6.1. Solidification of the optimized formula

Lyophilisation of the optimum (6)-loaded PEG-PCL modified was adopted to solidify the formula (Alpha 2–4, CHRIST, Osterodeam Harz, Germany), where the incorporated of 1% mannitol as cryoprotectant to constrain the fusion and lysis of nano-complexes. Consequently, the formula suspension was freezed overnight at −80 °C and dried for a period of 24 h under vacuum^61^.

#### 4.6.2. Differential scanning calorimetry (DSC)

Differential scanning calorimeter (DSC-50, Shimadzu, Kyoto, Japan) was employed in the assessment of the thermal pattern of pure 6, blank optimum formula, 6-loaded PEG-PCL modified NDs formula. Purified indium (99.9%) was involved in the calibration of the equipment. A gradual rise in the temperature at a rate of 10 °C/min was conducted, surrounded by nitrogen in a temperature range of 20–400 °C^61^.

#### 4.6.3. X-ray diffraction (XRD)

The extent of crystallinity of pure compound **6**, lyophilised blank optimum formula, and drug-loaded PEG-PCL modified ND was examined *via* X-Ray Diffractometer (Burker, Germany) with a Cu Ka radiation detector. The investigation was manipulated in 2*θ* range of 3.0–40° by a scan rate of 1 min^60^.

#### 4.6.4. Transmission electron microscopy (TEM)

TEM (Joel JEM 1230, Tokyo, Japan) was manipulated to visualise the morphology of the formulated NDs. The sample was stained, then adhered to a carbon grid with a copper coat and kept to dry to acquire a thin film. The copper sheet was visualised under TEM^53^.

#### 4.6.5. *In-vitro* release study of the optimal formula

Precisely, 1 ml of the optimised formula, corresponding to 1 mg of the compound under analysis was kept in a 10 cm in length and 2.5 cm in diameter glass cylinder down closed with a pre-soaked cellulose membrane where the dispersion was allowed to spread over its surface. Then the glass cylinder was hung to the shaft of the dissolution tester (Copley, DIS 8000, Nottingham, UK) and placed in 900 ml dissolution media (Sorensen phosphate buffer, pH 7.4) at 37 ± 0.5 °C and a speed of 50 rpm^57^. At different time interventions, the withdrawal of equal volumes from the dissolution media was conducted and investigated by HPLC at 252 nm to estimate the percentage of drug released. This *In-vitro* experiment was employed in triplicates.

## Supplementary Material

Supplemental Material
