# Supplementary material for: Design and synthesis of new trimethoxylphenyl-linked combretastatin analogues loaded on diamond nanoparticles as a panel for ameliorated solubility and antiproliferative activity
Source: J Enzyme Inhib Med Chem. 2022 Sep 26;37(1):2679–701. doi: 10.1080/14756366.2022.2116016 (PMC9518609; doi:10.1080/14756366.2022.2116016)
Supplement: Supplemental Material [file IENZ_A_2116016_SM1670.pdf]

Supporting Information

Design and Synthesis of New Trimethoxylphenyl-Linked Combretastatin Analogues loaded on diamond nanoparticles as a panel for ameliorated solubility and antiproliferative activity.

Islam Zaki [1,*], Amal M. Y. Moustafa [2], Botros Y. Beshay [3], Reham E. Masoud [4], Mohammed A. I. Elbastawesy [5], Mohammad A.S. Abourehab [6,7], Mohamed Y. Zakaria [8]

Part I: Biological Studies **PP 2-4**

Part II: NMR spectra of synthesized compounds 2a-13e **PP 5-45**

Part III: IC_{50} values of compounds 2a-13e in **PP 46**

Part I: Biological Studies

4.3. Biological evaluation

4.3.1. Cytotoxic activity evaluation

The cytotoxic actvity of the synthesized derivatives **2a-13e** was measured in MDA-MB-231 breast cancer cell line and derivatives **6** and **12** in MCF-10A breast cancer cell line. Cell viability assay was assessed using MTT assay method. Cells at density of 1 x 10^4 were seeded in a 96-well plate at 37 ^oC for 24 h under 5% CO_2. After incubation, the cells were treated with different concentrations of the test schiff bases **2a-13e** and incubated for 24 h, then 20 µl of MTT solution at 5 mg/mL was applied and incubated for 4 h at 37 °C. Dimethyl sulphoxide (DMSO) in volume of 100 µl was added to each well to dissolve the purple formazan that had formed. The color intensity of the formazan product, which represents the growth condition of the cells, is quantified by using an ELISA plate reader (EXL 800, USA) at 570 nm absorbance. The experimental conditions were carried out with at least three replicates, and the experiments were repeated at least three times.

4.3.2. Tubulin inhibitions Assay

Compounds **6, 12** and CA-4 were evaluated for their tubulin inhibitory activity according to manufacturer's instructions using # abcam Human Beta-tubulin simplestep ELISA Kit ab245722. MDA-MB-231 cells were trypsinized, counted and seeded at the 2 × 10^5 into 96-well micro titer plates. Cells then were incubated in a humidified atmosphere at 37 ^oC for 20-24 h. The standards, the tested compounds, and the positive reference CA-4 were diluted to designated concentrations. On the 96-well micro titer plates standard, sample or reference compound was added to each well in 100 µL, and incubated at 37 ^oC for 2 h. The solution was aspirated and 100 µL of prepared Detection Reagent A was added to each well. Incubation was done at 37 ^oC for 2 h. After washing 100 µL of prepared Detection Reagent B was added and incubation was continued at 37 ^oC for 30 min. Five washings were done, then 90 µL of 3,3',5,5'-tetramethylbenzidine (TMB) substrate solution was added and incubated at 37 ^oC for 15-25 min. Stop solution was added in 50 µL. Cells were exposed to IC_{50} concentration of compounds **6**, **12** and SAHA for 72 h. Optical density (O.D.) was measured at 450±10 nm using microplate reader (Spectromax Plus 96 well plate spectrophotometer).

Information Classification: General

4.3.3. Cell cycle analysis of compound 6 and 12

Cell cycle analysis in MDA-MB-231 cells was investigated using fluorescent Annexin V-FITC/ PI detection kit (*BioVision* EZCell™ Cell Cycle Analysis Kit Catalog #K920) by flow cytometry assay. MDA-MB-231 cells at a density of 2×10^5 per well were harvested and washed twice in PBS. After that, the cells were incubated at 37 °C and 5% CO_2. The medium was incubated with the tested compounds **6** and **12** at their IC_{50} (μM) for 24 h, washed twice in PBS, fixed with 70% ethanol, rinsed again with PBS. Afterward, medium was stained with DNA fluorochrome PI for 15 min at 37 °C. The samples were immediately analyzed using Facs Calibur flow cytometer (Becton and Dickinson, Heidelberg, Germany).

4.3.4. Apoptosis studies

4.3.4.1. Apoptosis assay for compounds 6 and 12

Apoptosis in MDA-MB-231 cells was investigated using fluorescent Annexin V-FITC/ PI detection kit (*BioVision* Annexin V-FITC Apoptosis Detection Kit, Catalog #: K101) by flow cytometry assay. MDA-MB-231 cells at a density of 2×10^5 per well were treated with compounds **6** and **12** at their IC_{50} (μM) for 24 h, then the cells were harvested and stained with Annexin V-FITC/ PI dye for 15 min in the dark at 37 °C. The samples were immediately analyzed using *FACS Calibur* flow cytometer (Becton and Dickinson, Heidelberg, Germany).

4.3.4.2. Effect on p53, Bax and Bcl-2

p53, Bax and Bcl2 enzyme activities in MDA-MB-231 cells were detected in the presence of compounds **6** and **12** at their IC_{50} concentration (μM). The levels of the tumor suppressor gene p53, apoptotic markers Bax as well as the anti-apoptotic marker Bcl-2 were assessed using p53 ELISA kit, Human Bax ELISA kit and Bcl-2 Elisa kit. The procedure of the used kits was done according to the manufacturer's instructions. Briefly, Cell lysates were prepared from control and MDA-MB-231 cells (2.5×10^5/mL) treated with IC_{50} concentration of compounds **6** and **12**. Then equal amounts of cell lysates were loaded then probed with specific antibodies. The samples were measured at 450 nm in ROBONEK P2000 ELISA reader. Analysis was confirmed with three different sets of extracts. All experiments were done in triplicates.

4.3.4.3. *C*aspase 3/7 assay for compounds 6 and 12

*C*aspase 3/7 in MDA-MB-231 cells was investigated using CellEvent® *C*aspase 3/7 Green Detection Flow Cytometry Assay Kit**.** *C*aspase 3/7 activity in MDA-MB-231 cell line was detected in the presence of compounds **6** and **12** at their IC_{50} concentration (μM) using CellEvent® *c*aspase 3/7 green detection flow cytometry assay kit according to manufacturer's directions. HepG2 cells at a density of 2.5×10^5 per well were harvested and washed with ice cold PBS, and cell lysates were prepared and combined with reaction buffer and incubated with specific colorimetric substrates (*C*aspase 3/7 Detection Reagent) at 37 ^{o}C for 6 h. Detection reagent is composed of DNA binding dye conjugated to *c*aspase 3 and *c*aspase 7 recognition sequence (DEVD) by a short peptide linker. The released DNA binding dye binds DNA producing a bright and stable signal. Finally, the samples were analyzed at 488 nm in a BD *FACS Calibur* flow cytometer. All experiments were performed in triplicates.

Part II: NMR spectra of synthesized compounds 2a-13e

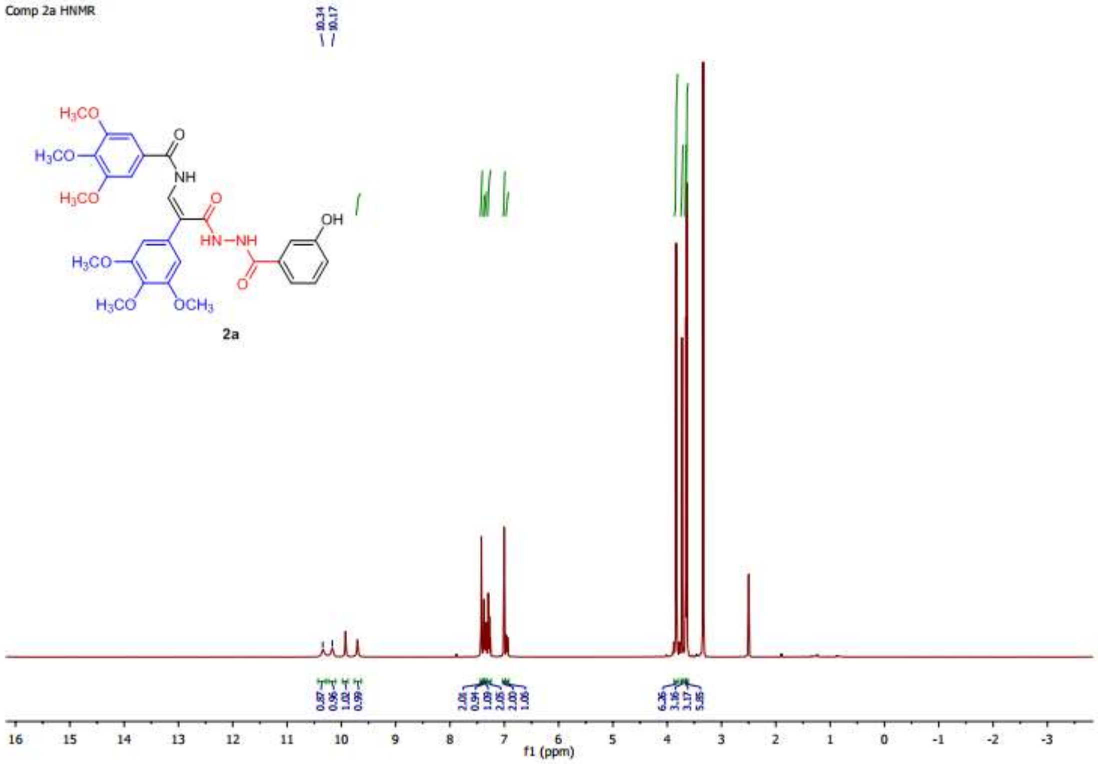

Figure S1: ^{1}H-NMR spectrum of compound **2a**

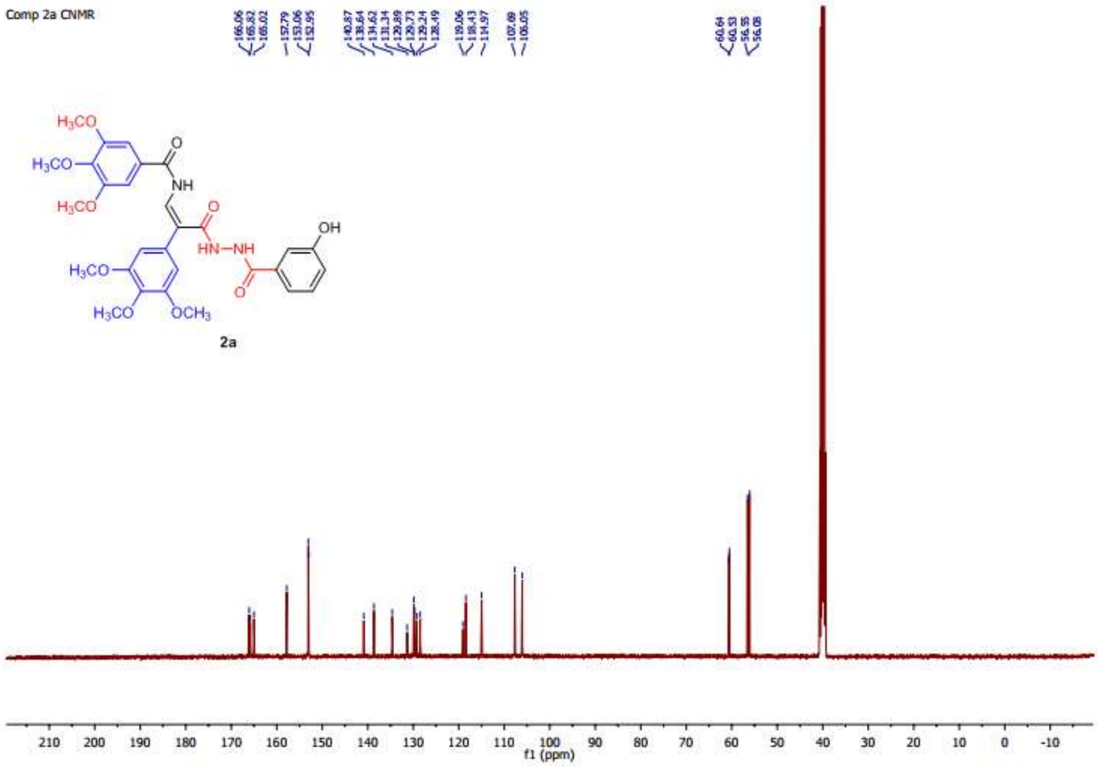

Figure S2: ^{13}C-NMR spectrum of compound **2a**

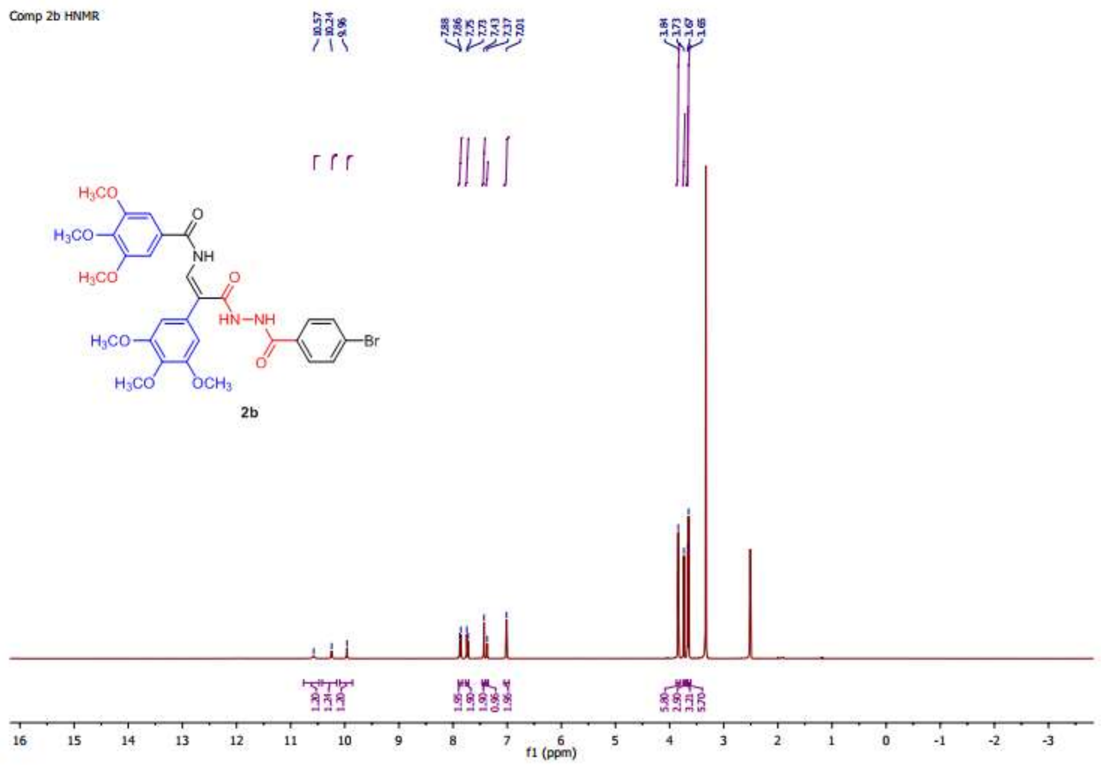

Figure S3: ^{1}H-NMR spectrum of compound **2b**

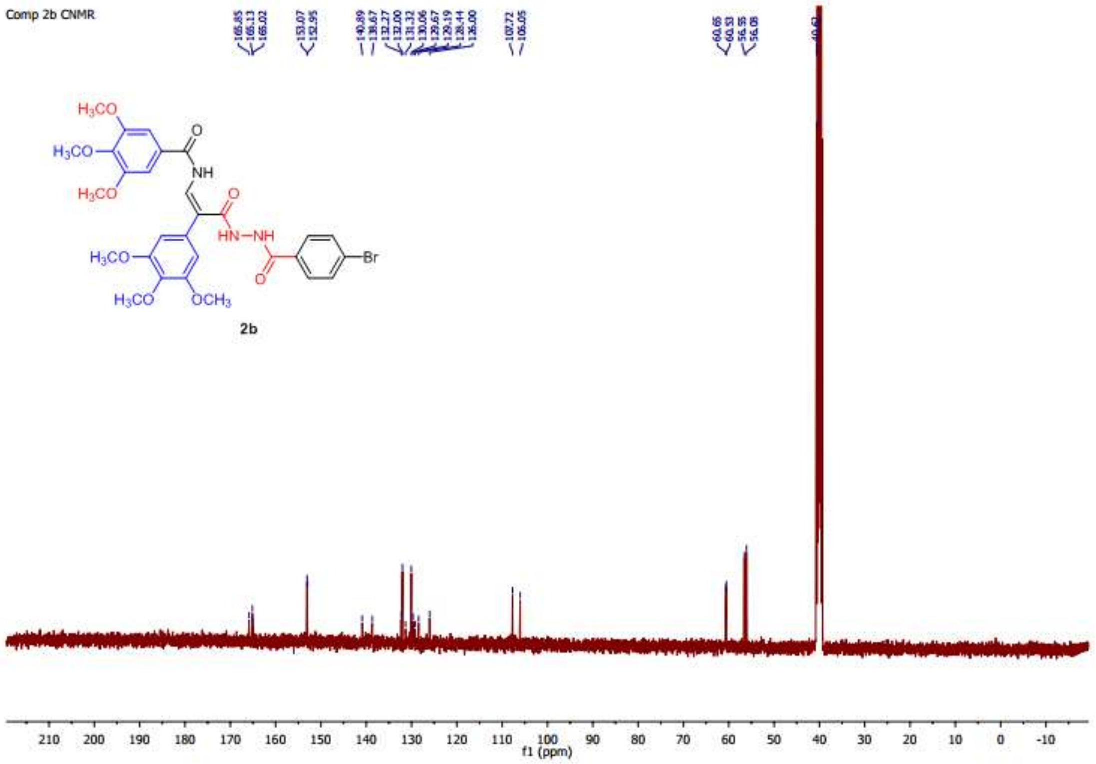

Figure S4: ^{13}C-NMR spectrum of compound **2b**

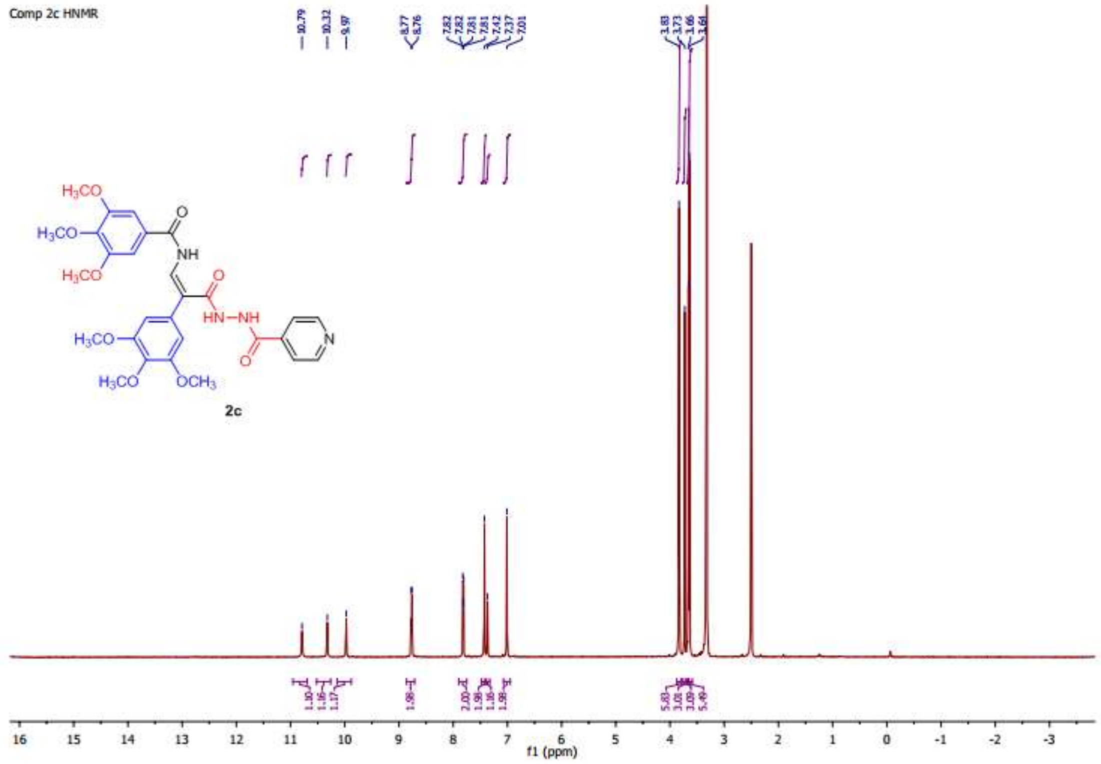

Figure S5: ^{1}H-NMR spectrum of compound **2c**

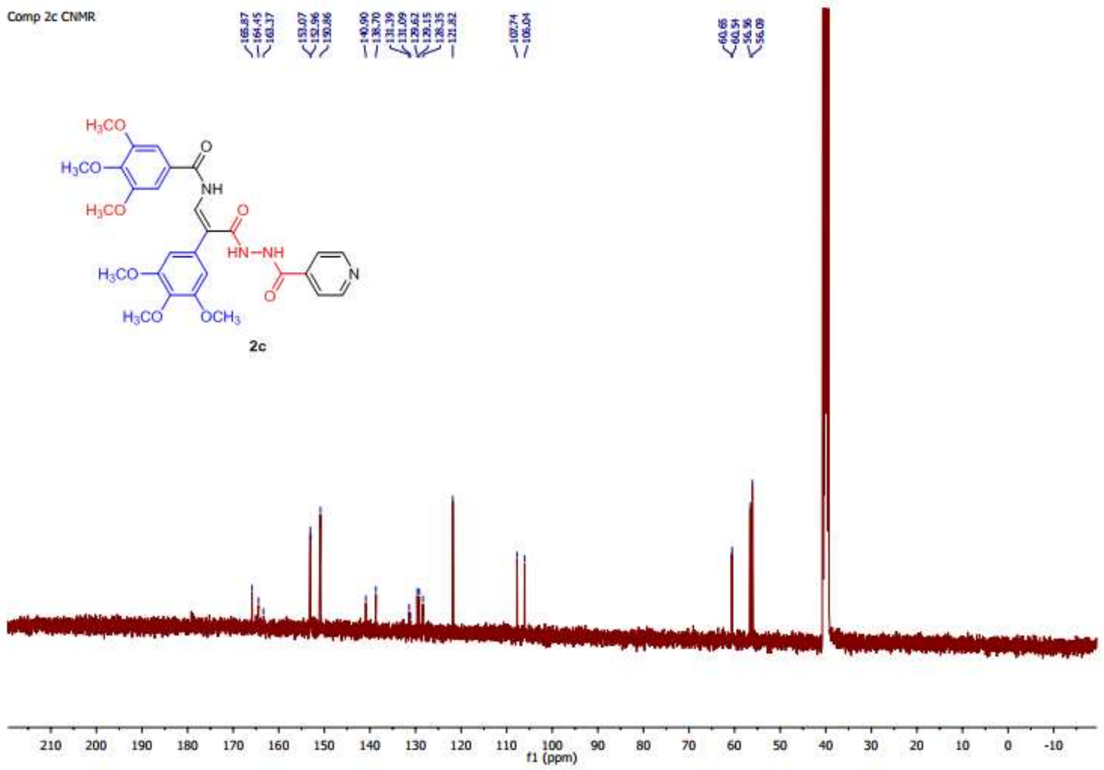

Figure S6: ^{13}C-NMR spectrum of compound **2c**

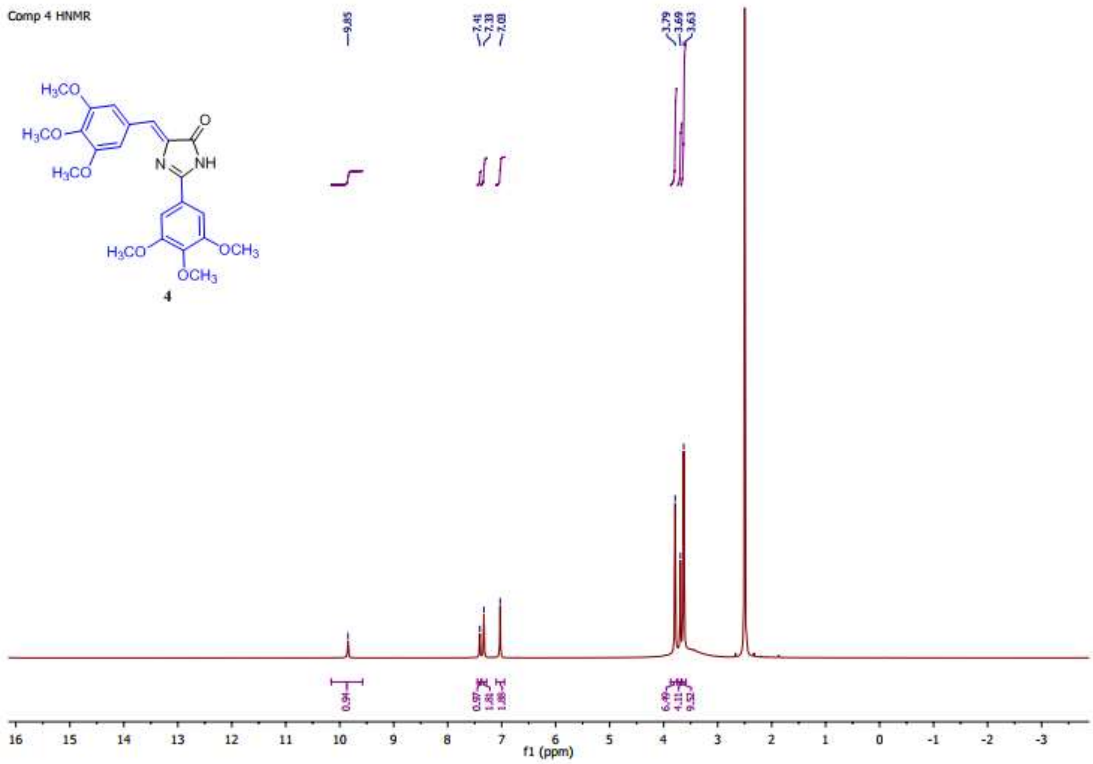

Figure S7: ^{1}H-NMR spectrum of compound **4**

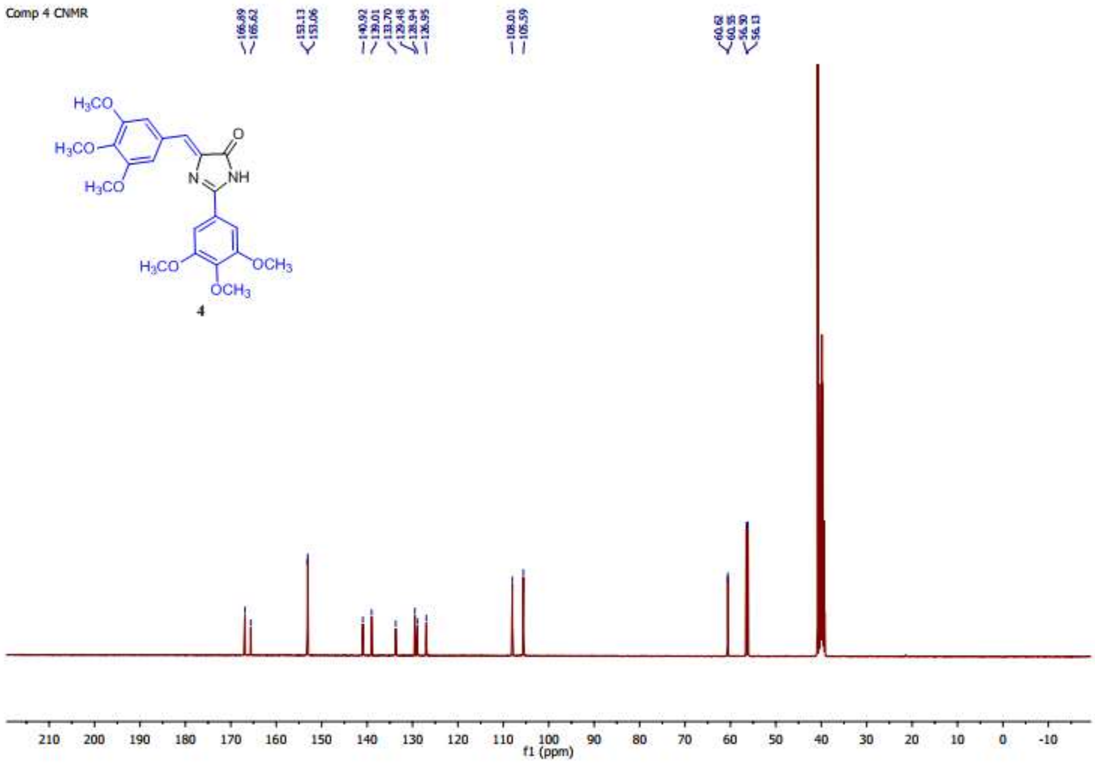

Figure S8: ^{13}C-NMR spectrum of compound **4**

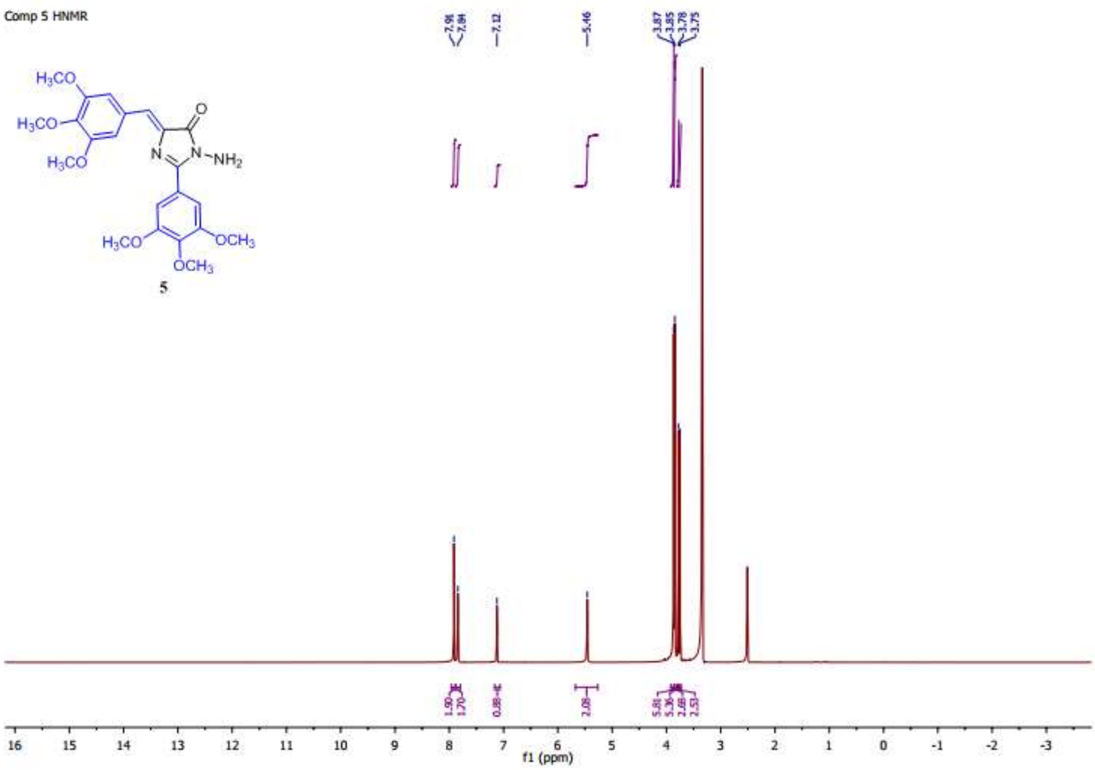

Figure S9: ^{1}H-NMR spectrum of compound **5**

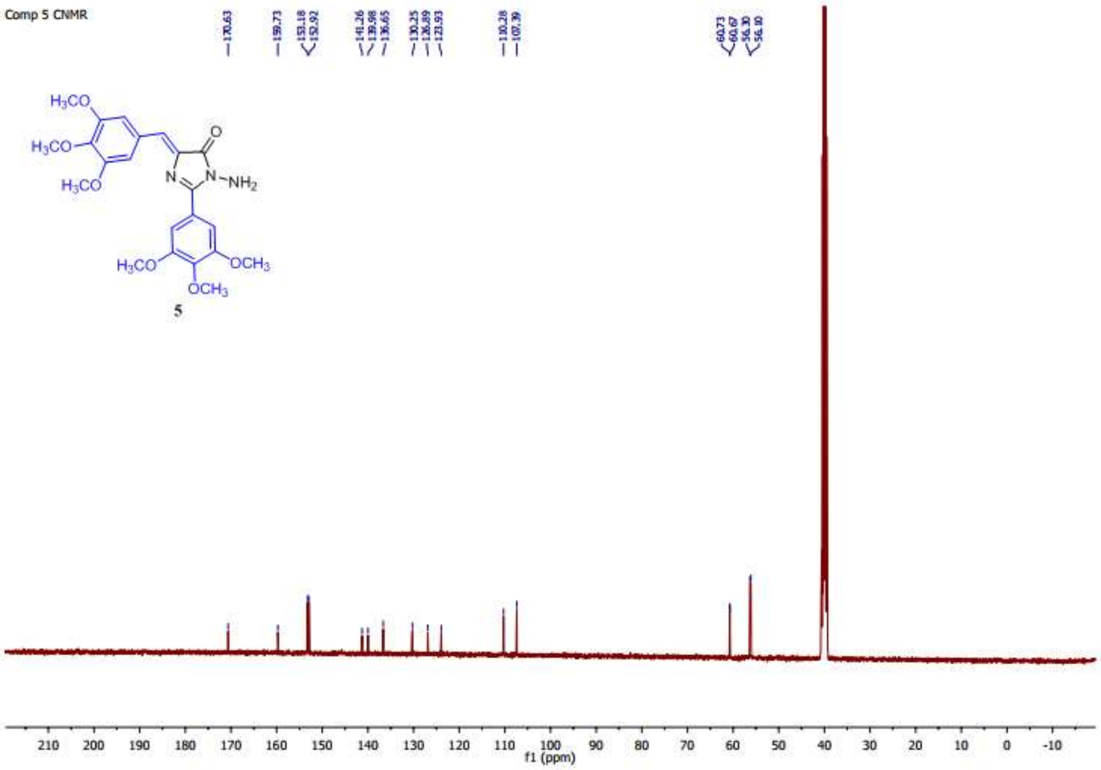

Figure S10: ^{13}C-NMR spectrum of compound **5**

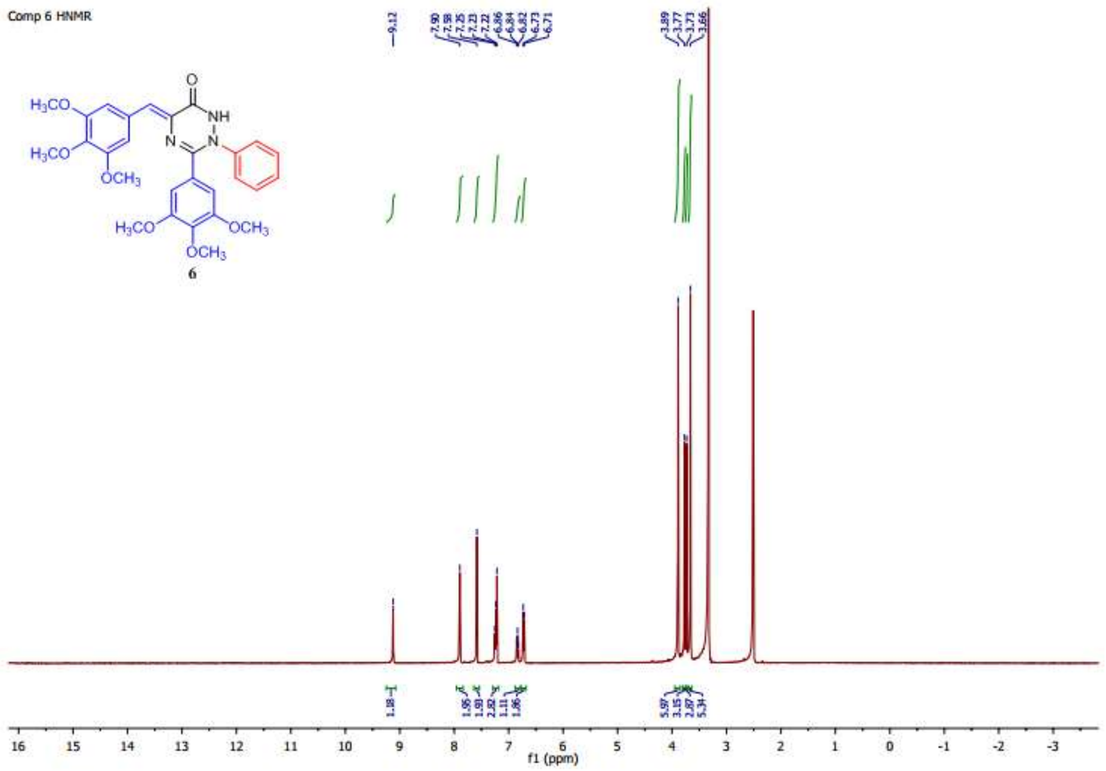

Figure S11: ^{1}H-NMR spectrum of compound **6**

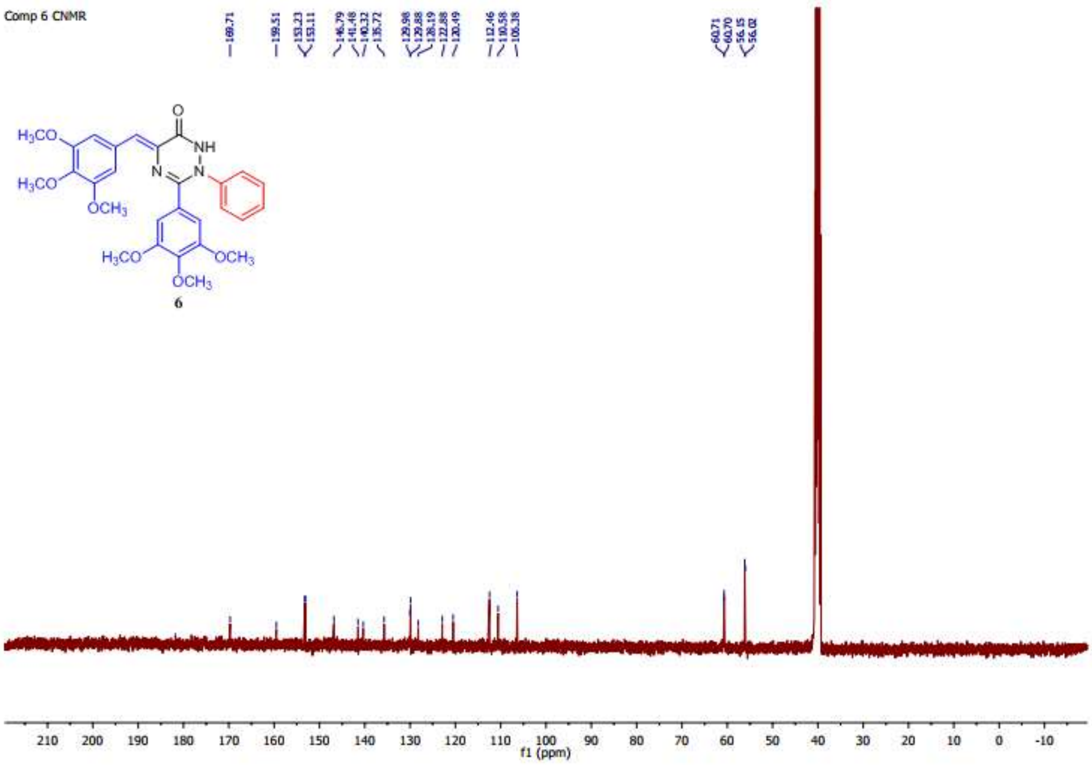

Figure S12: ^{13}C-NMR spectrum of compound **6**

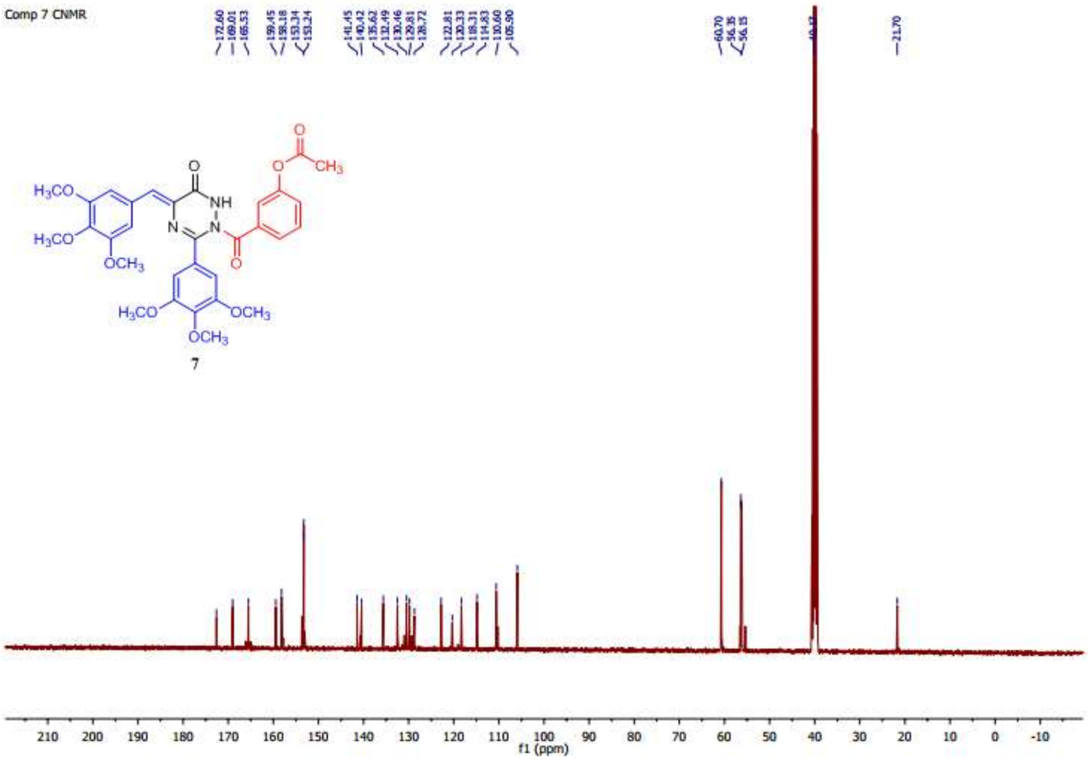

Figure S13: ^{13}C-NMR spectrum of compound **7**

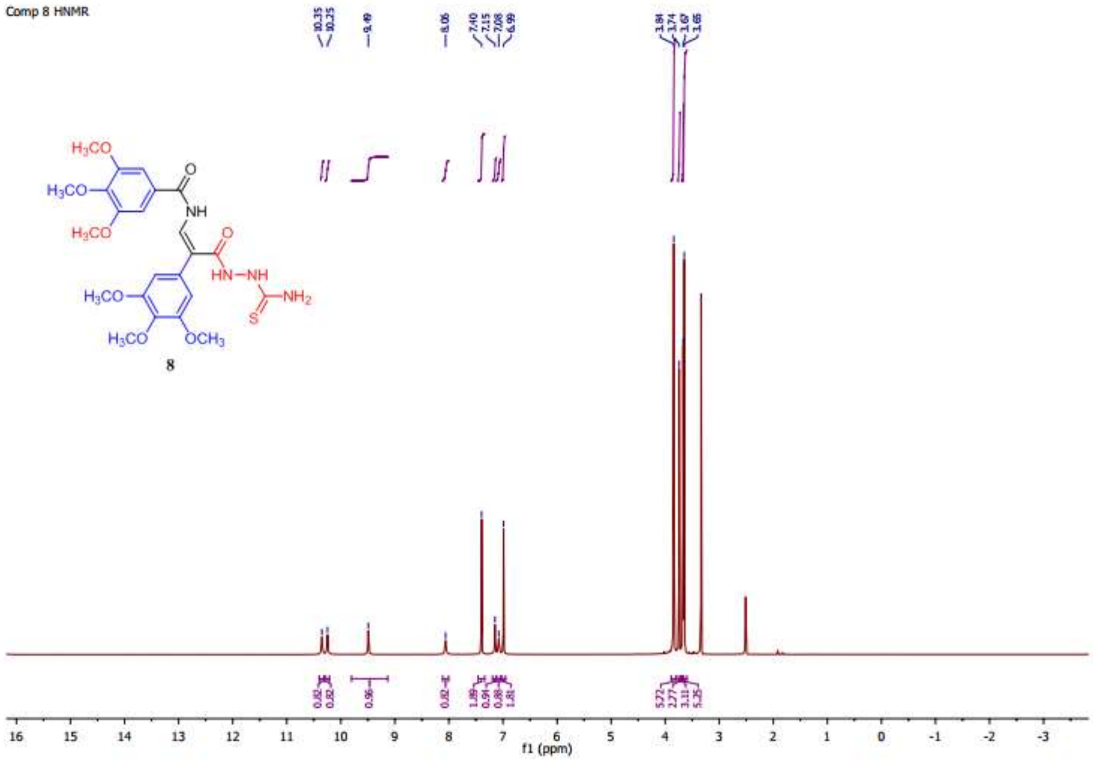

Figure S14: ^{1}H-NMR spectrum of compound **8**

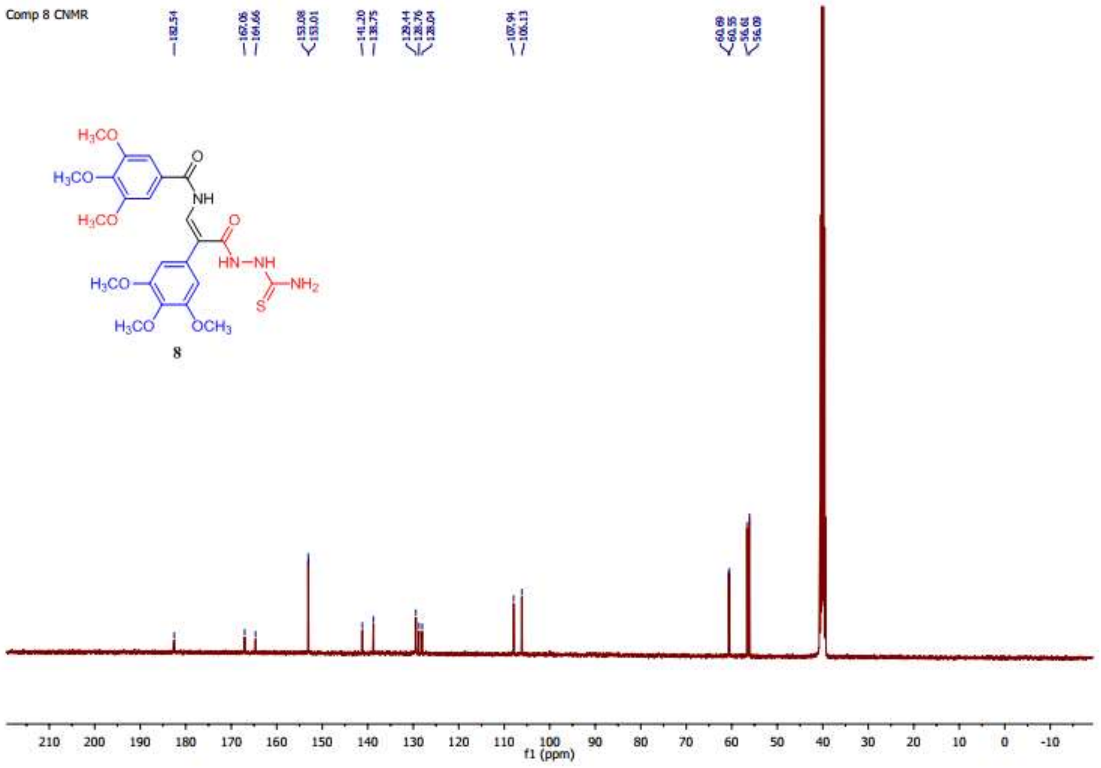

Figure S15: ^{13}C-NMR spectrum of compound **8**

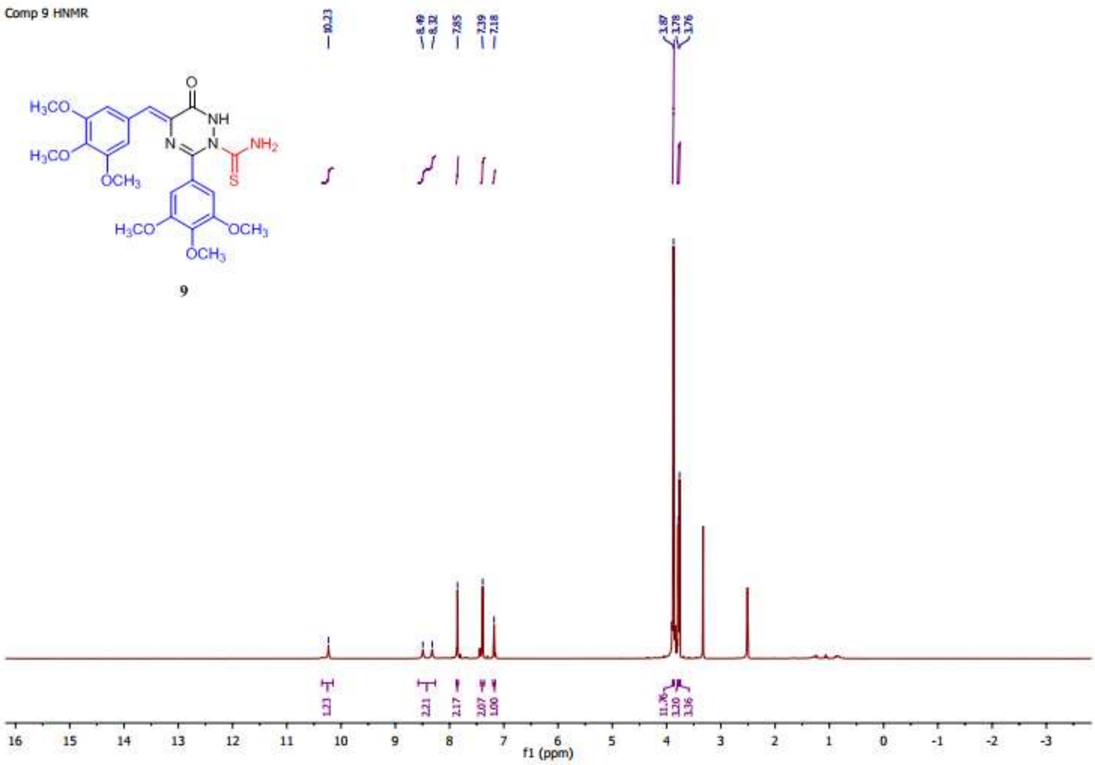

Figure S16: ^{1}H-NMR spectrum of compound **9**

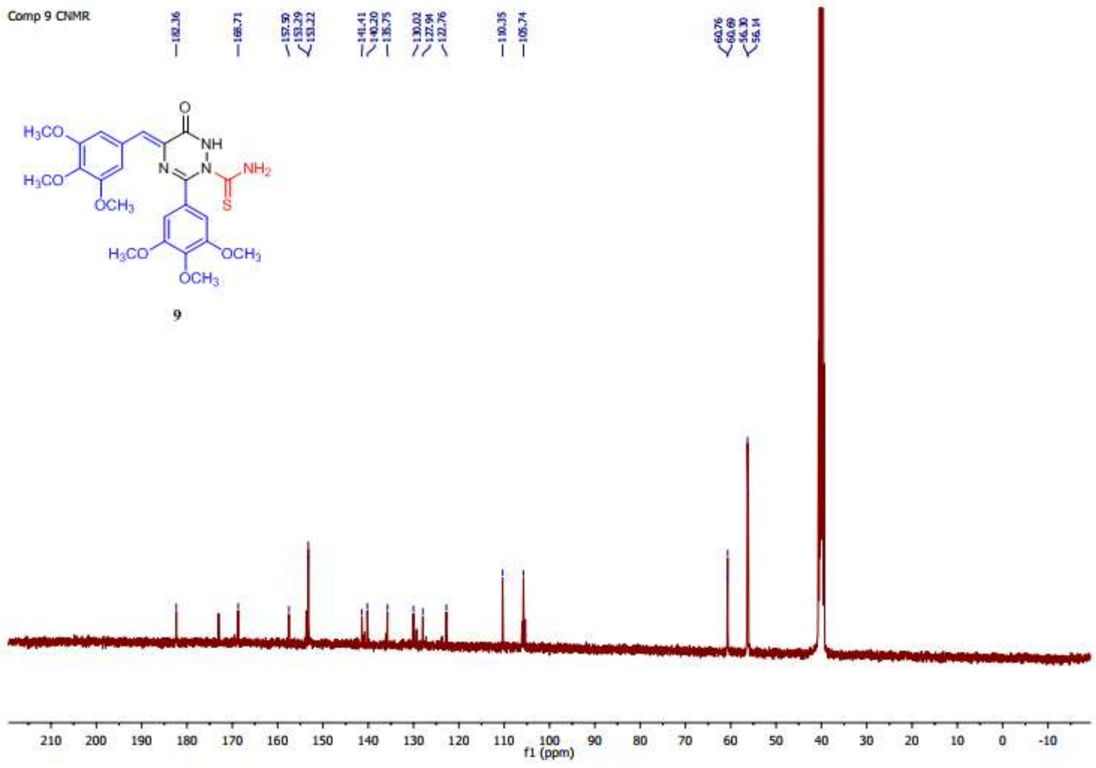

Figure S17: ^{13}C-NMR spectrum of compound **9**

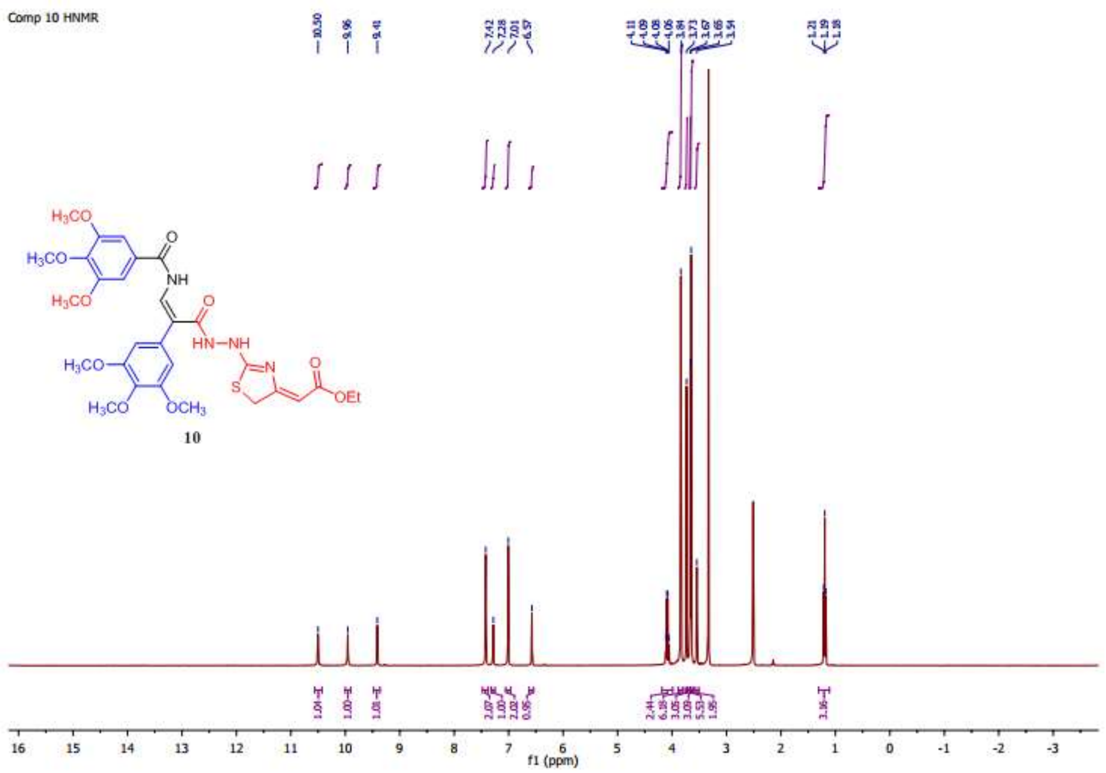

Figure S18: ^{1}H-NMR spectrum of compound **10**

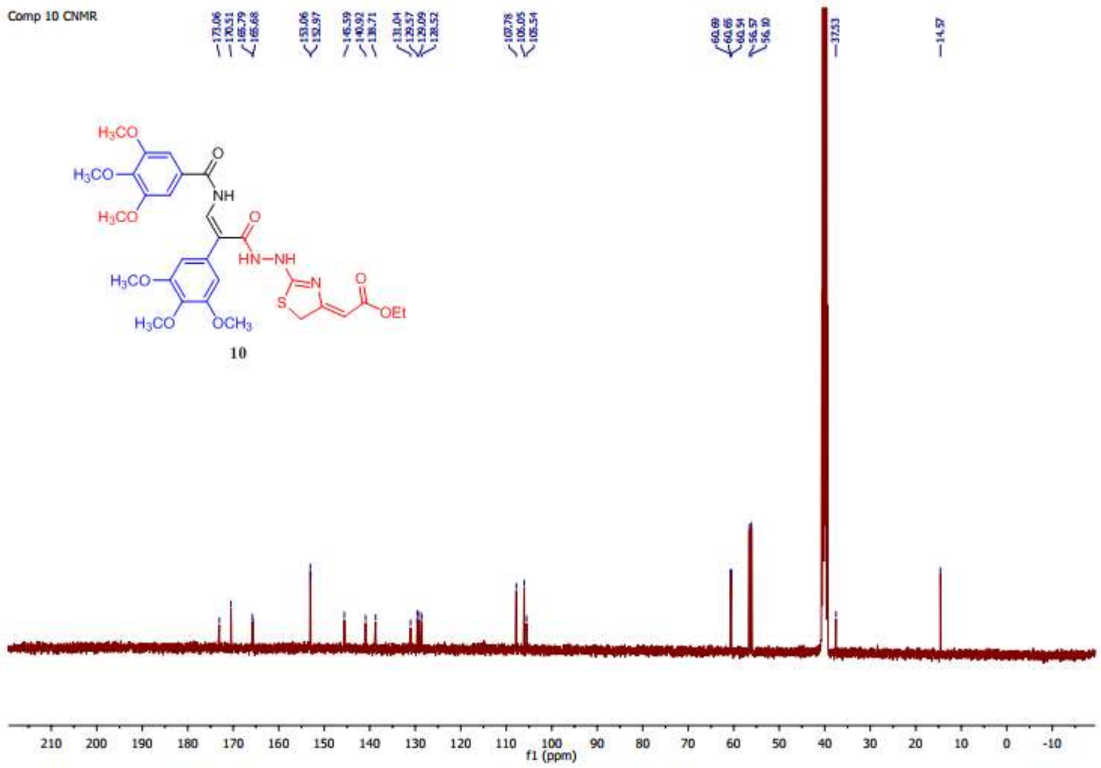

Figure S19: ^{13}C-NMR spectrum of compound **10**

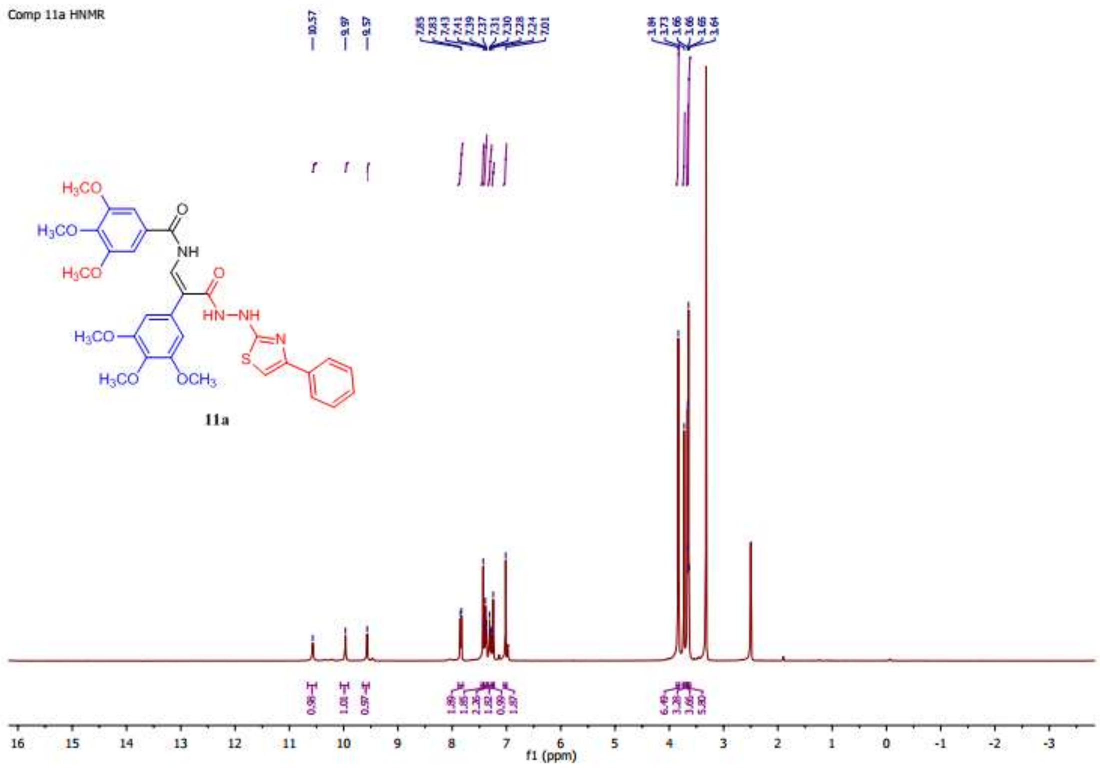

Figure S20: ^{1}H-NMR spectrum of compound **11a**

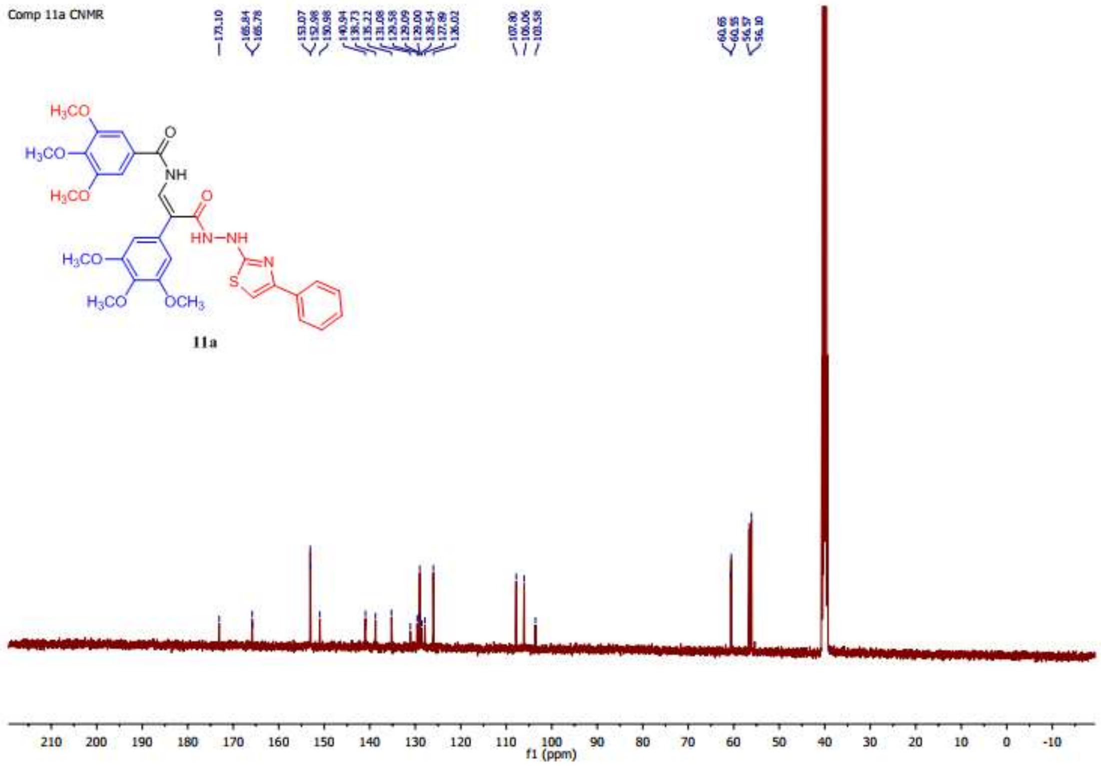

Figure S21: ^{13}C-NMR spectrum of compound **11a**

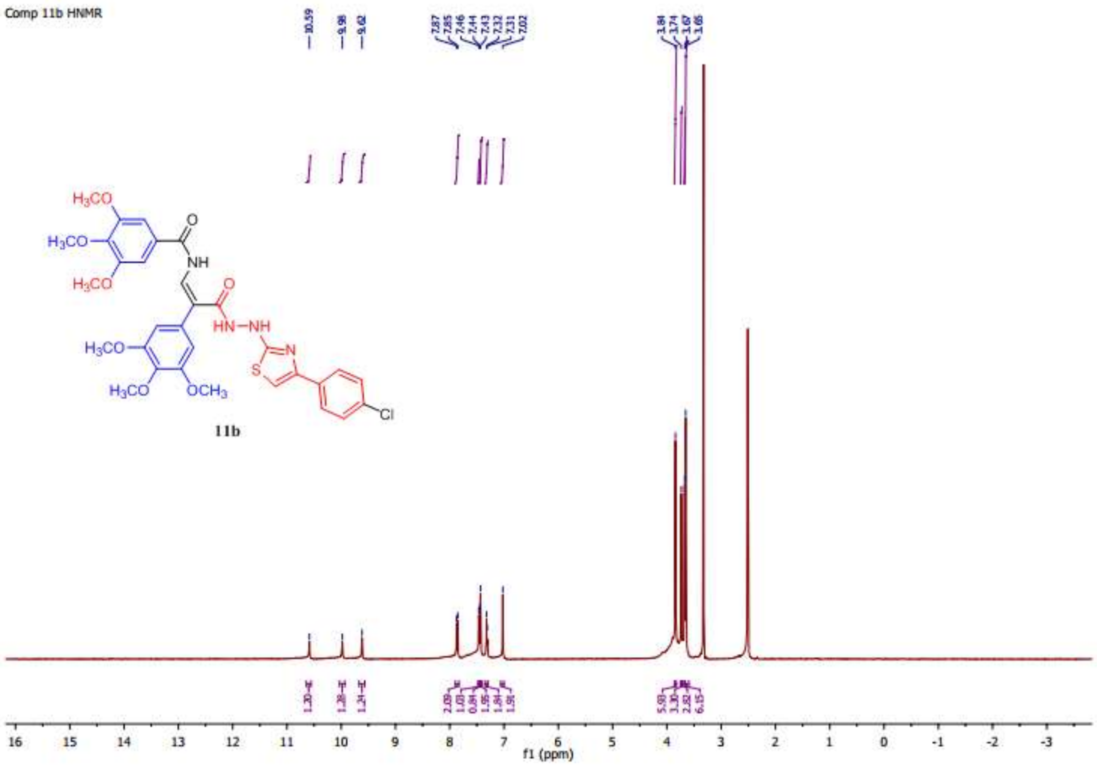

Figure S22: ^{1}H-NMR spectrum of compound **11b**

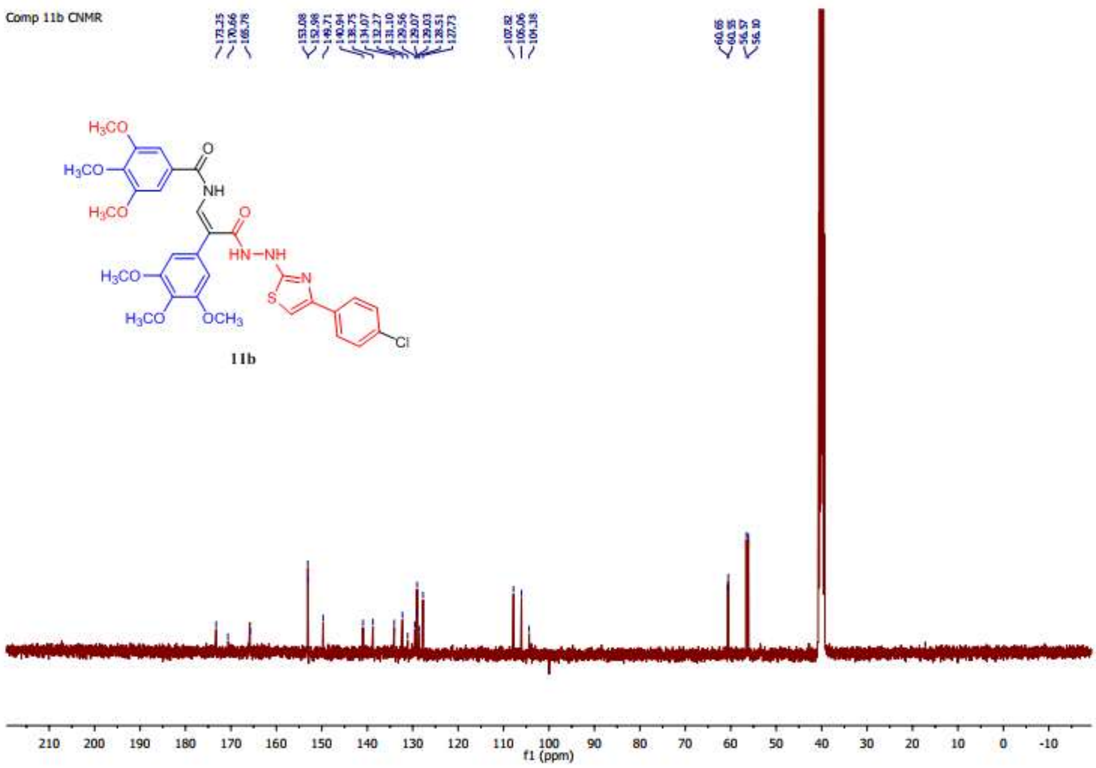

Figure S23: ^{13}C-NMR spectrum of compound **11b**

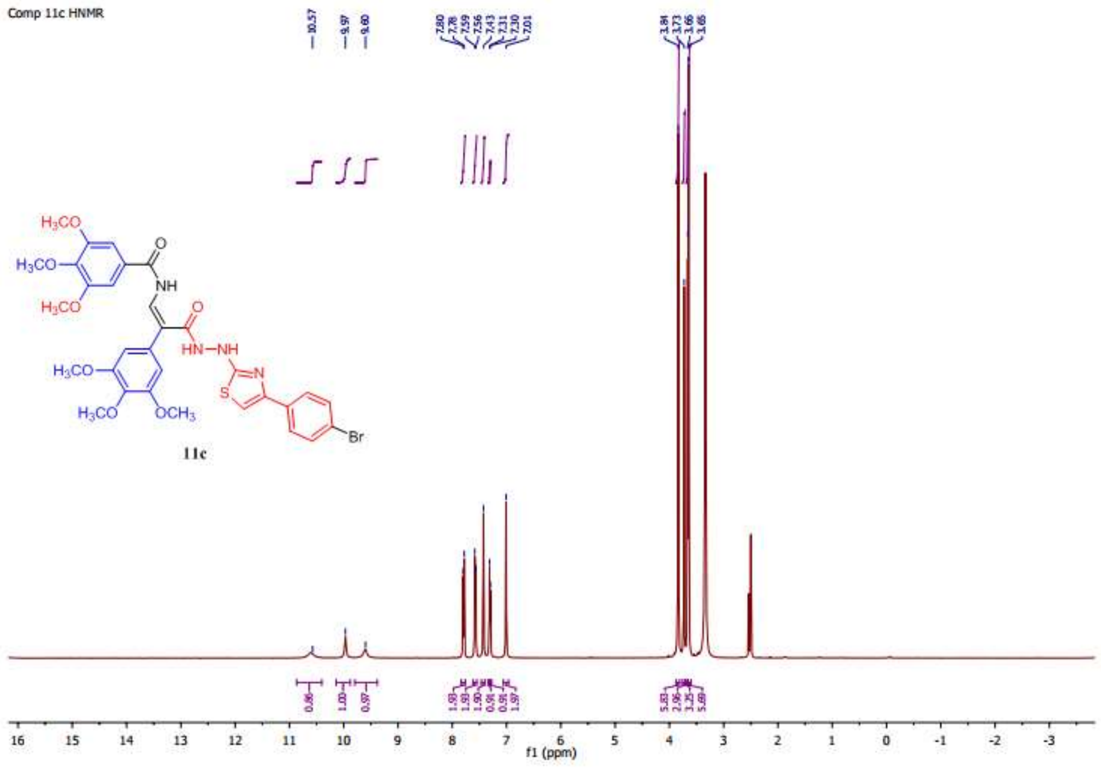

Figure S24: ^{1}H-NMR spectrum of compound **11c**

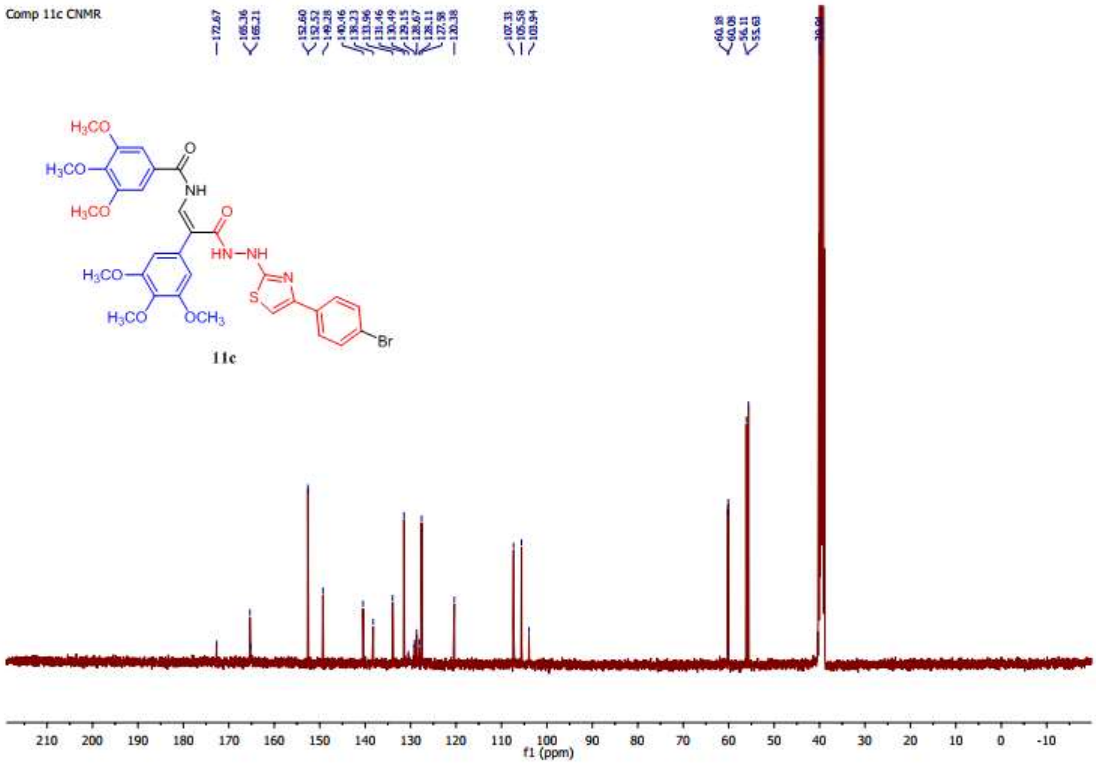

Figure S25: ^{13}C-NMR spectrum of compound **11c**

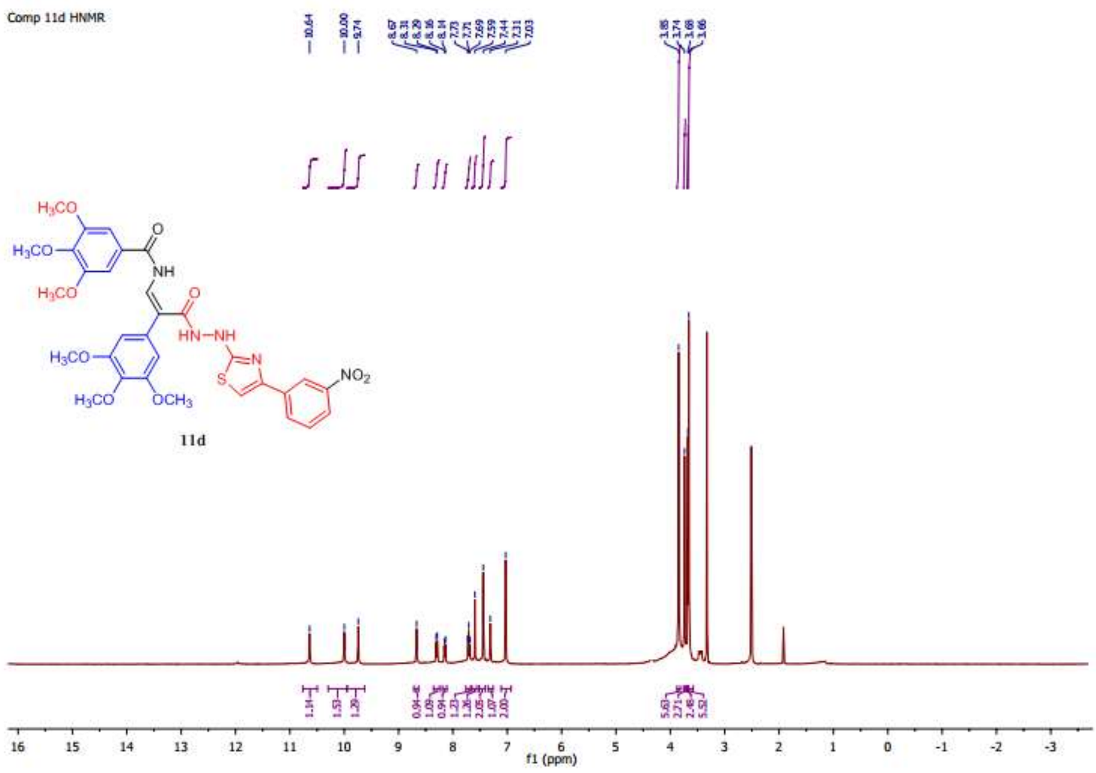

Figure S26: ^{1}H-NMR spectrum of compound **11d**

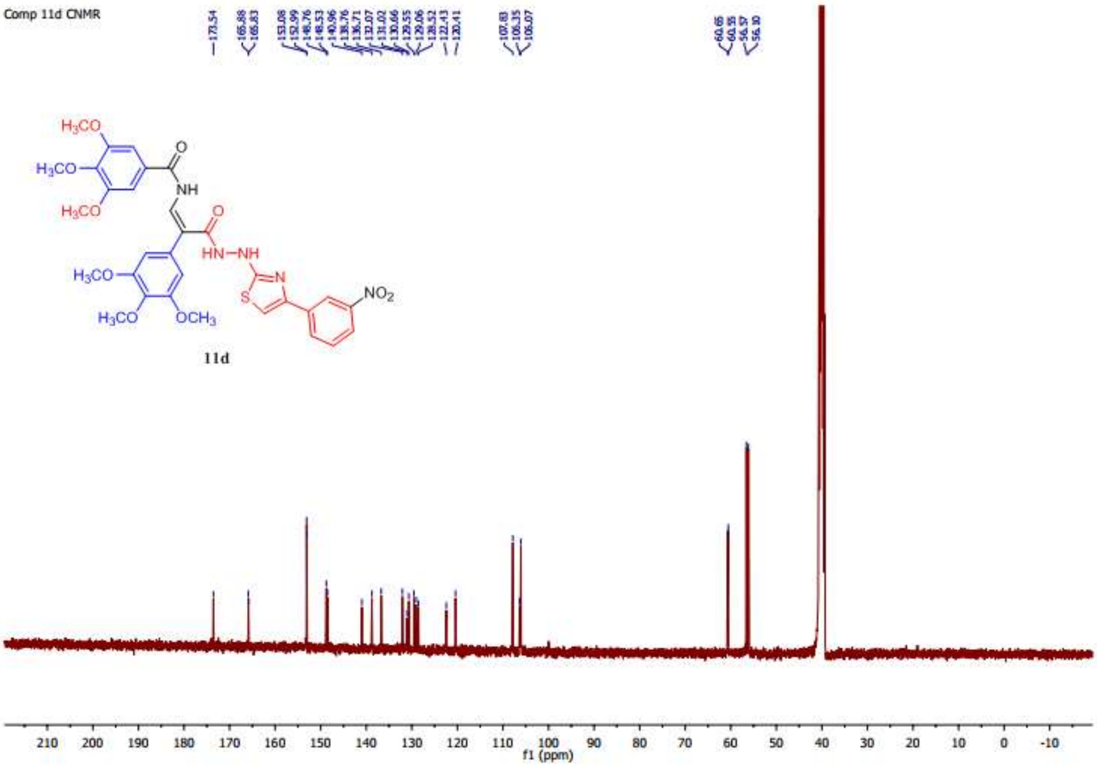

Figure S27: ^{13}C-NMR spectrum of compound **11d**

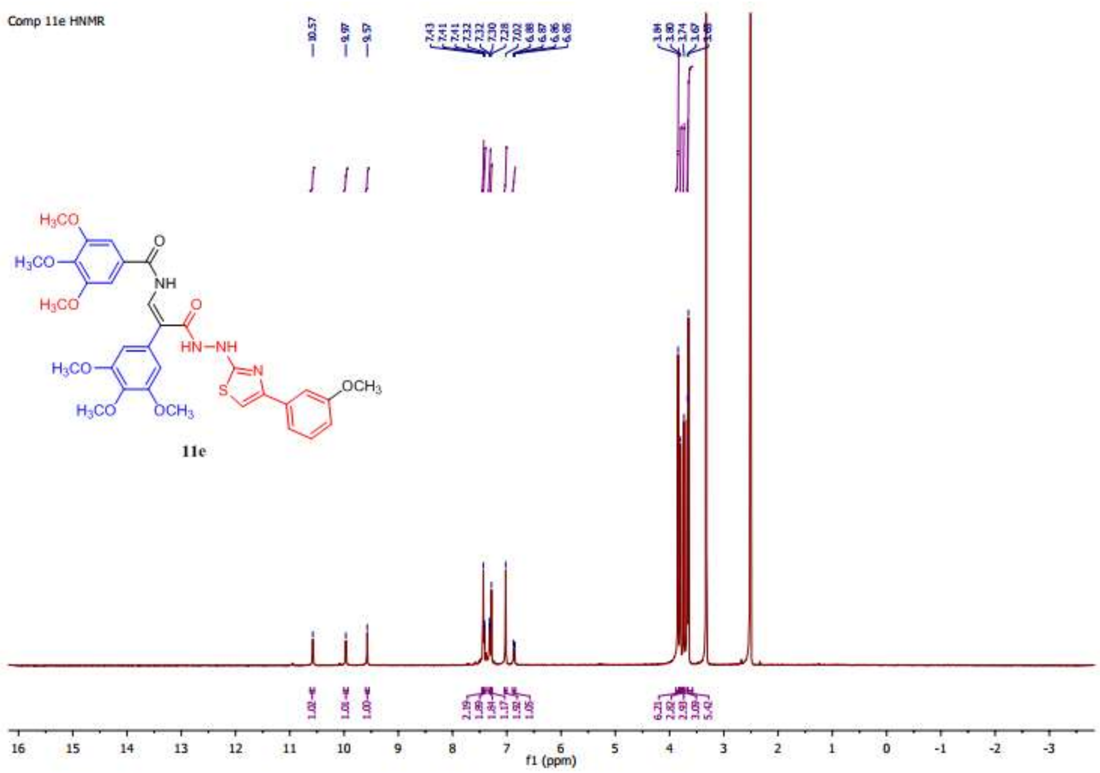

Figure S28: ^{1}H-NMR spectrum of compound **11e**

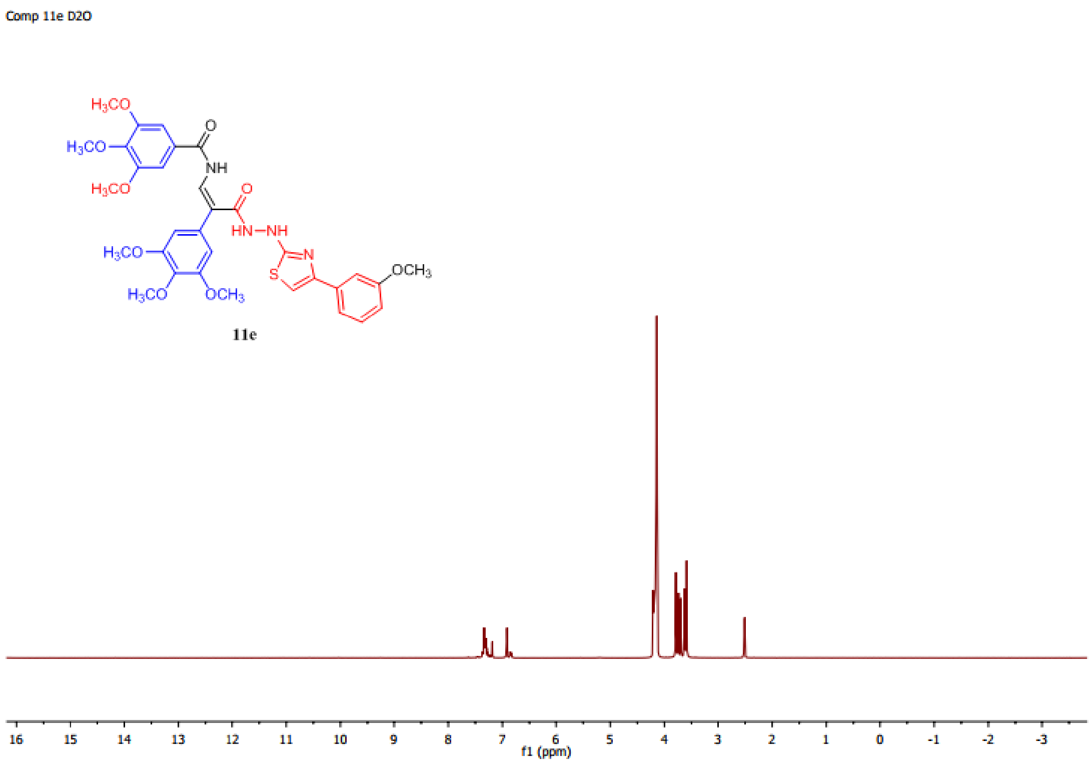

Figure S29: ^{1}H-NMR spectrum of compound **11e** in the presence of D_2O

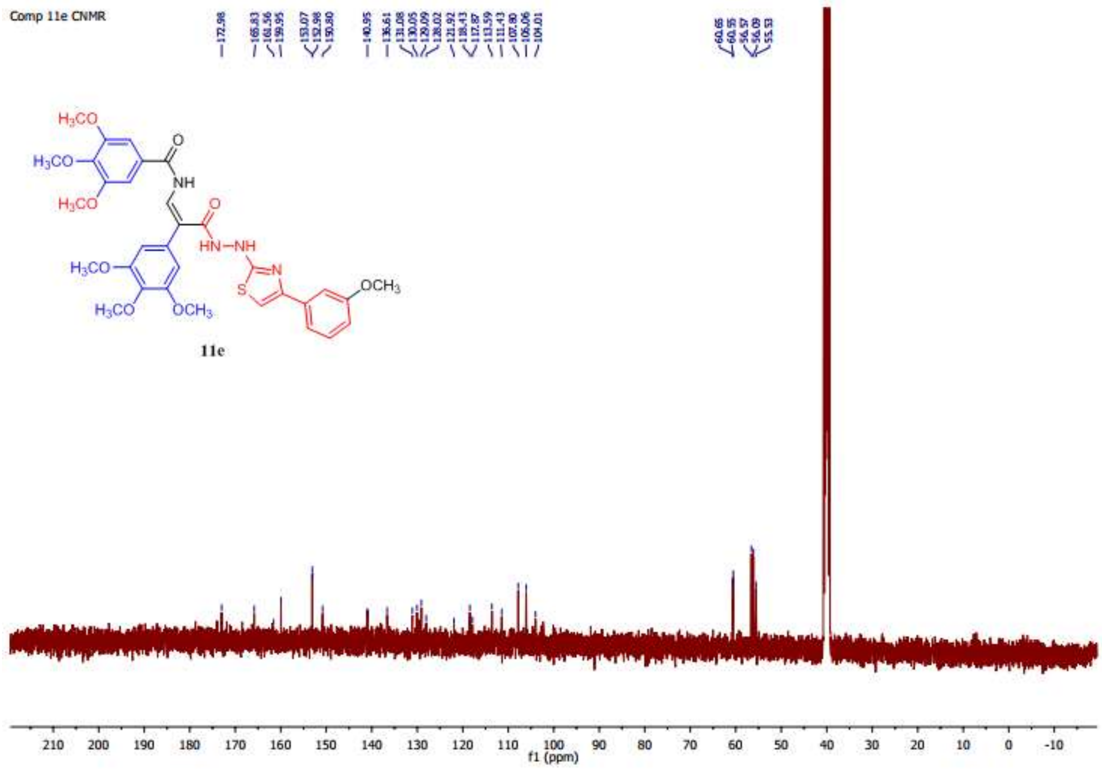

Figure S30: ^{13}C-NMR spectrum of compound **11e**

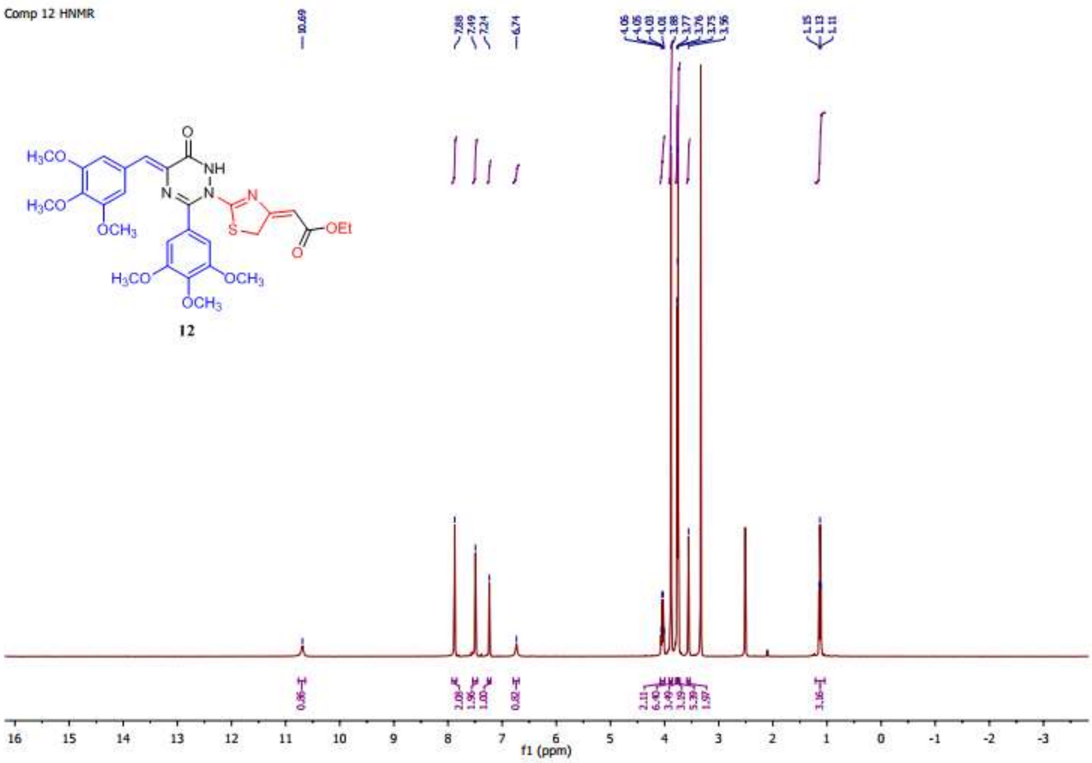

Figure S31: ^{1}H-NMR spectrum of compound **12**

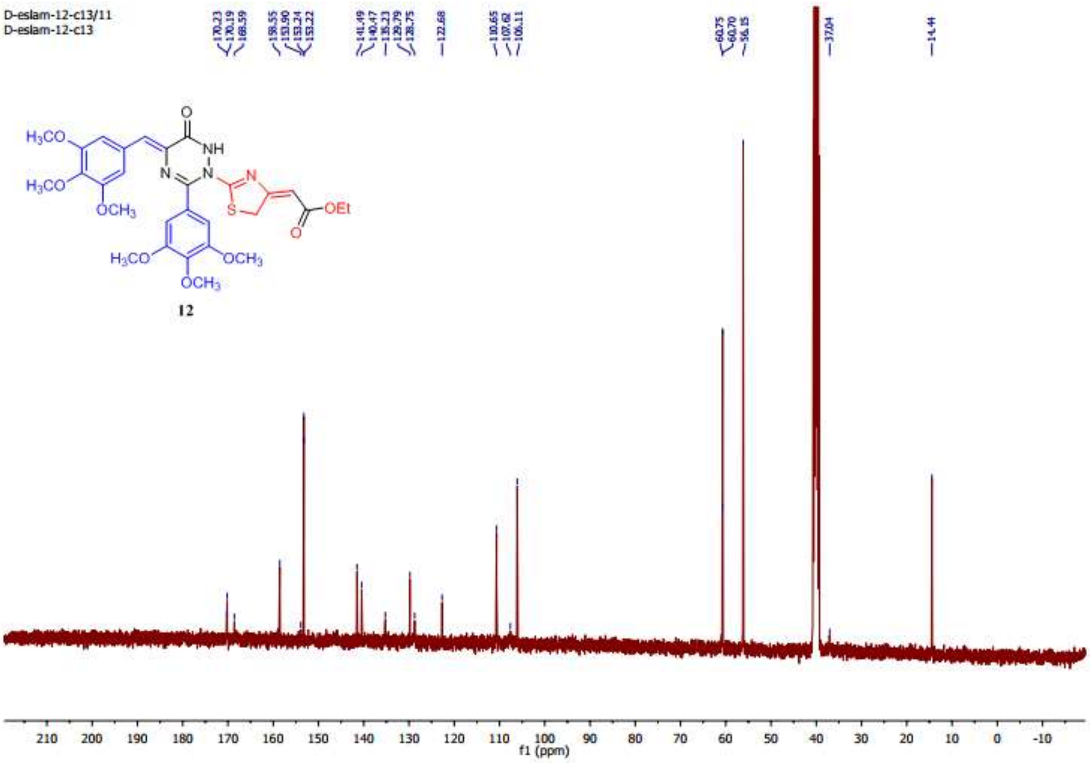

Figure S32: ^{13}C-NMR spectrum of compound **12**

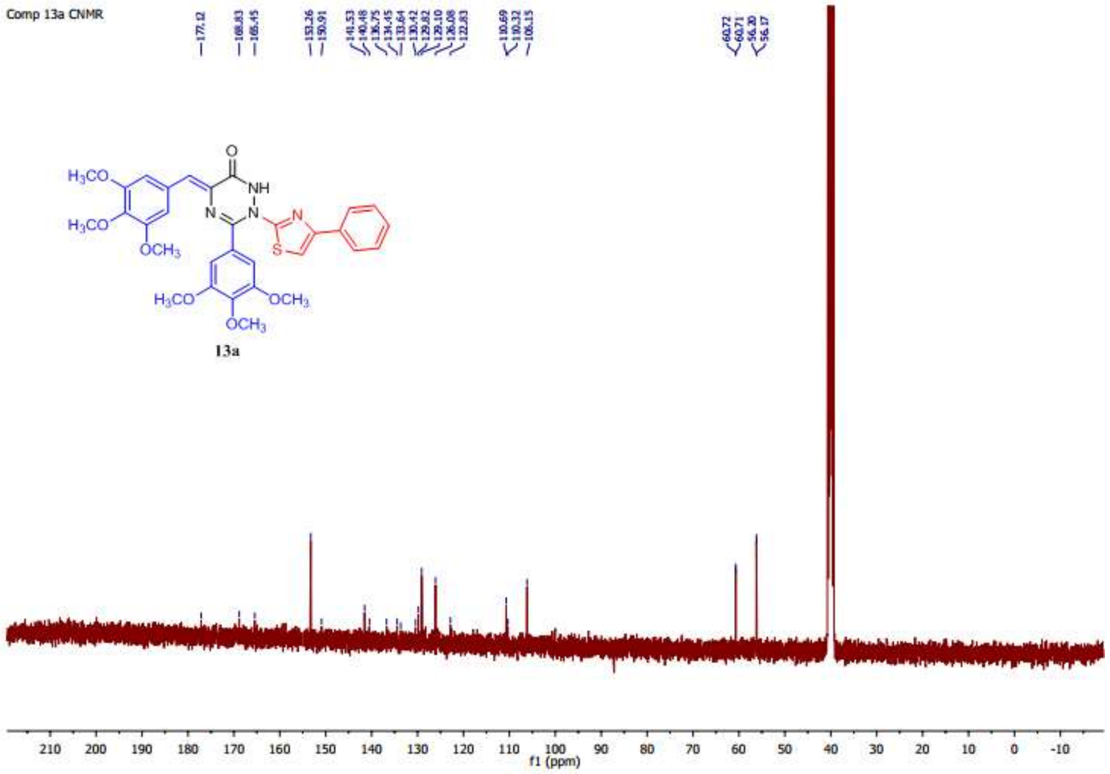

Figure S33: ^{13}C-NMR spectrum of compound **13a**

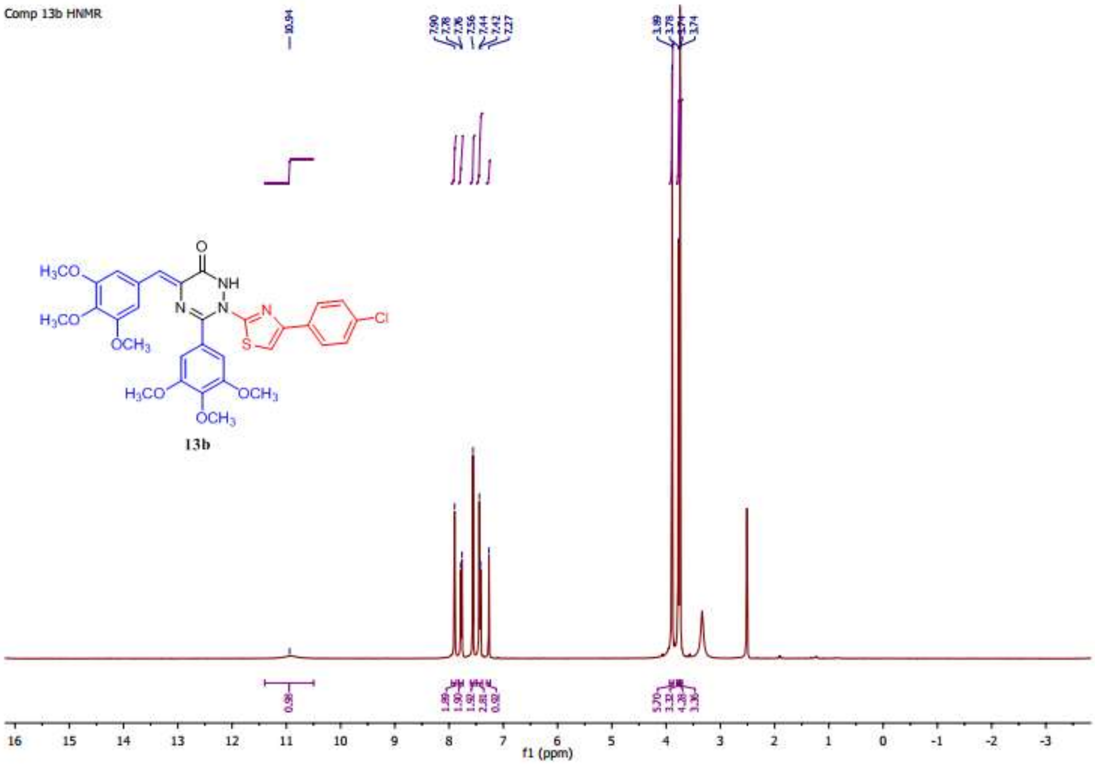

Figure S34: ^{1}H-NMR spectrum of compound **13b**

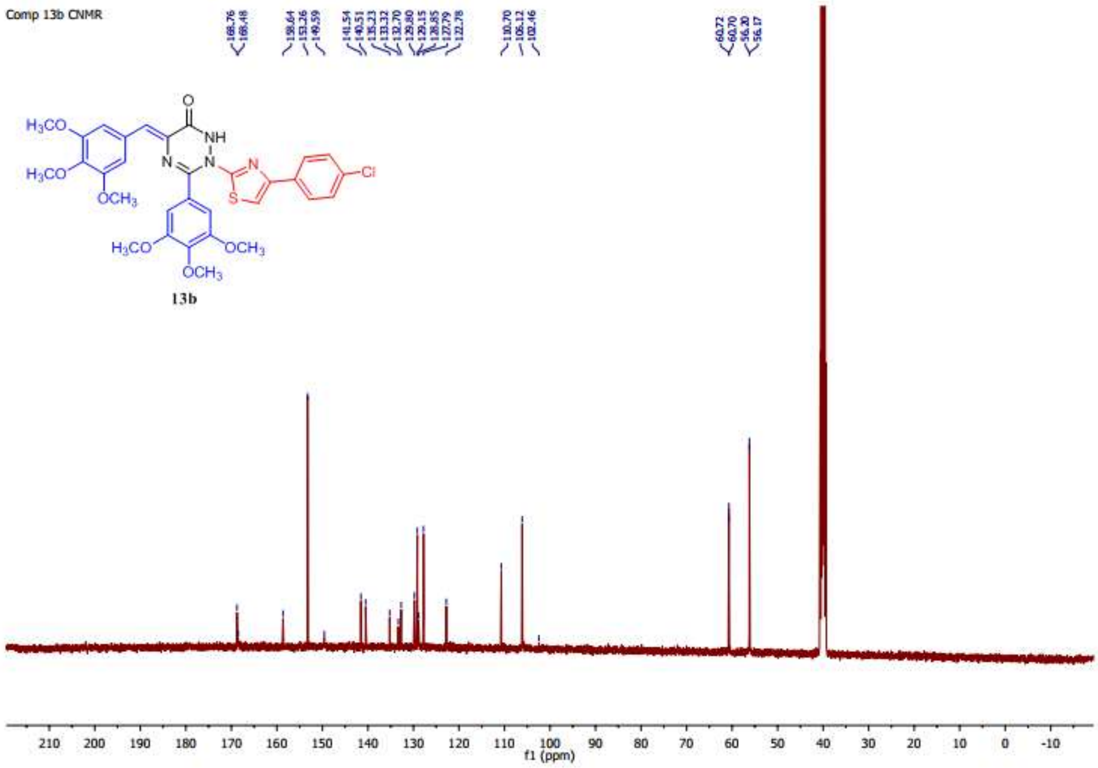

Figure S35: ^{13}C-NMR spectrum of compound **13b**

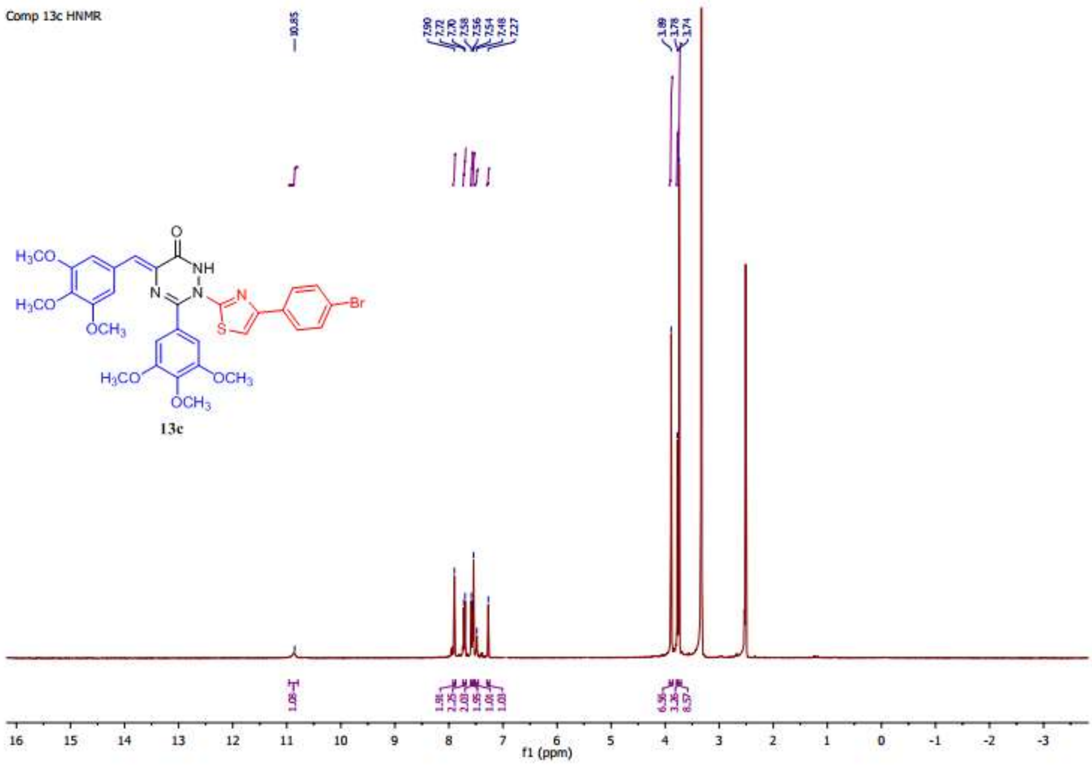

Figure S36: ^{1}H-NMR spectrum of compound **13c**

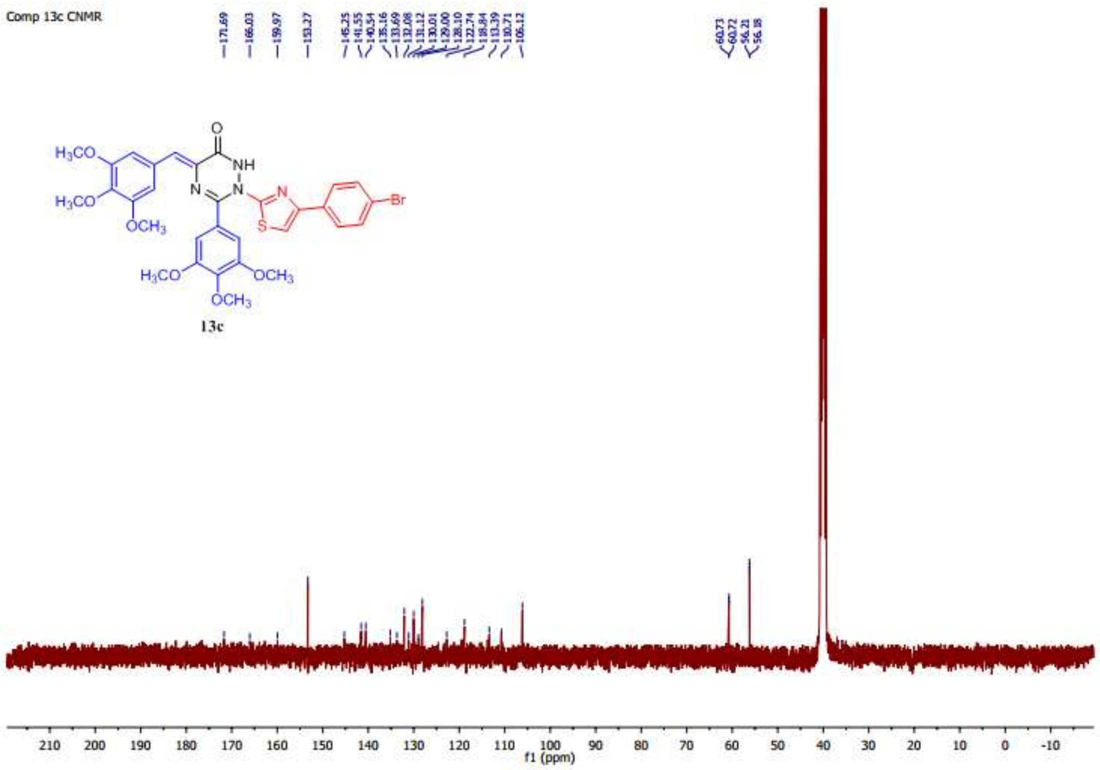

Figure S37: ^{13}C-NMR spectrum of compound **13c**

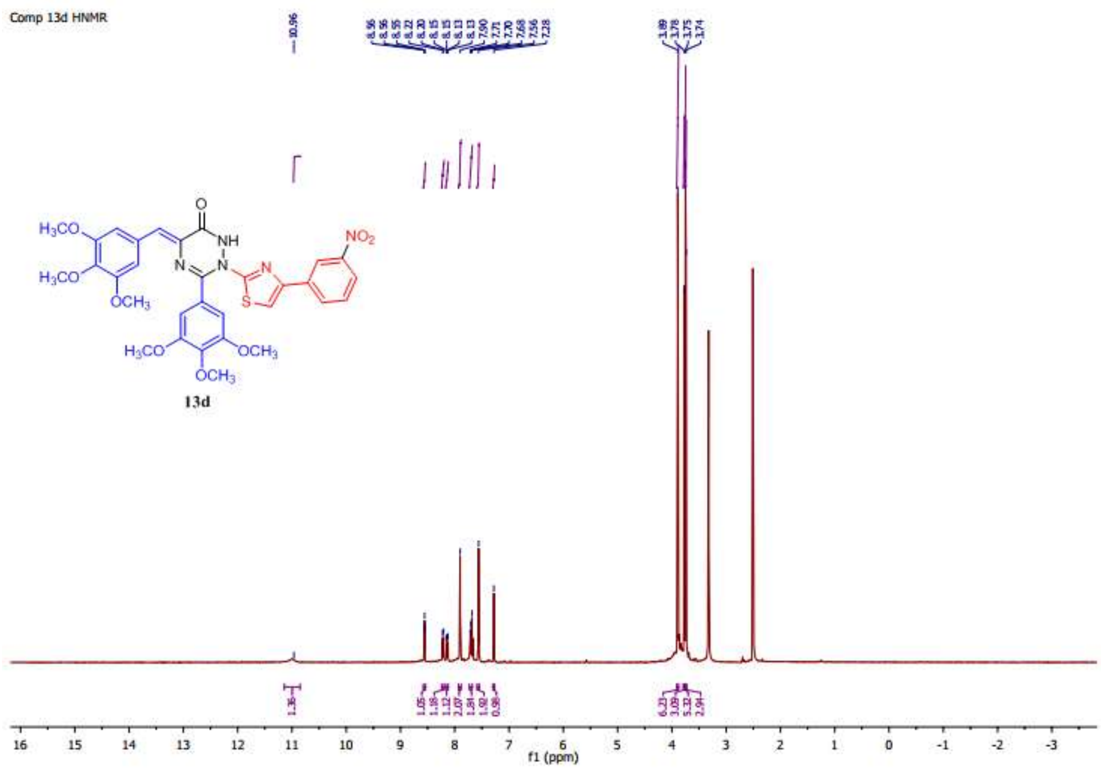

Figure S38: ^{1}H-NMR spectrum of compound **13d**

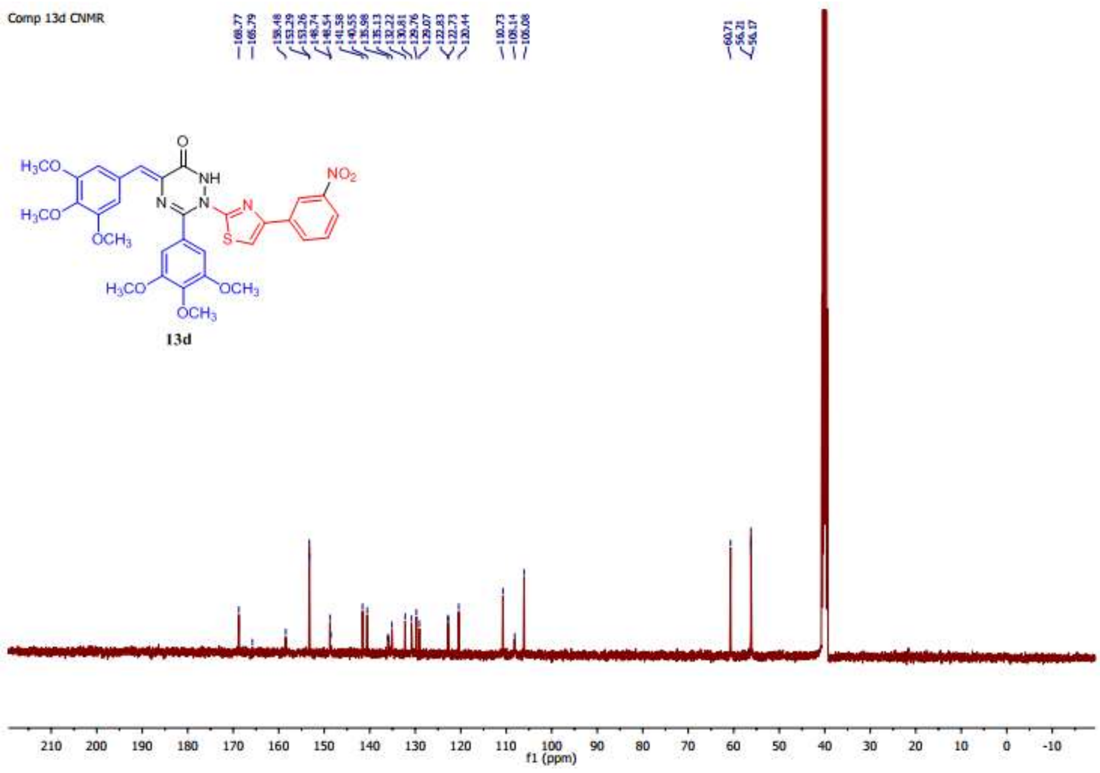

Figure S39: ^{13}C-NMR spectrum of compound **13d**

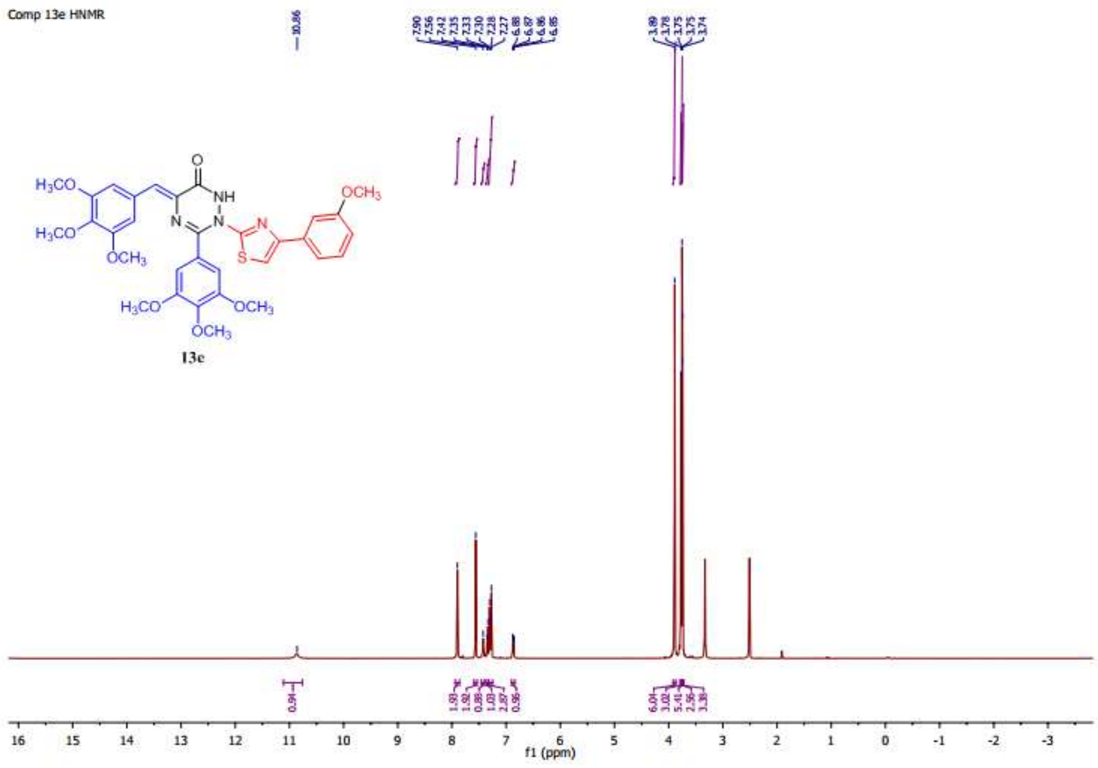

Figure S40: ^{1}H-NMR spectrum of compound **13e**

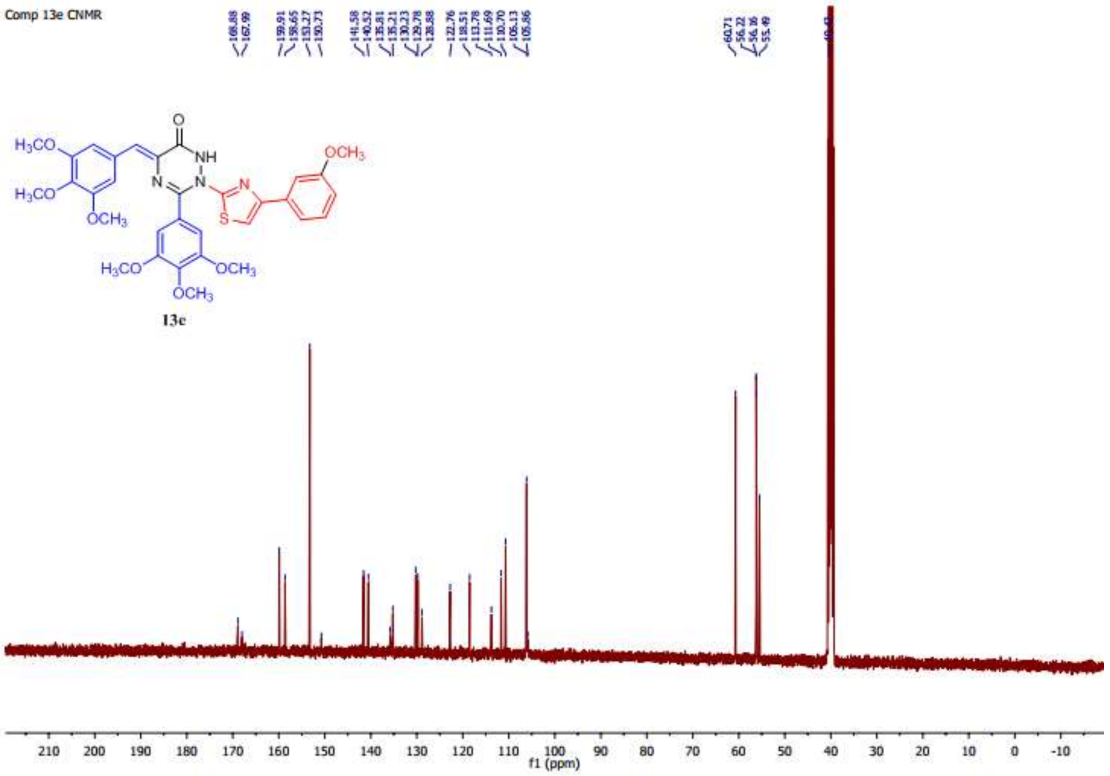

Figure S41: ^{13}C-NMR spectrum of compound **13e**

Part III: IC_{50} values of the prepared compounds 2a-13e

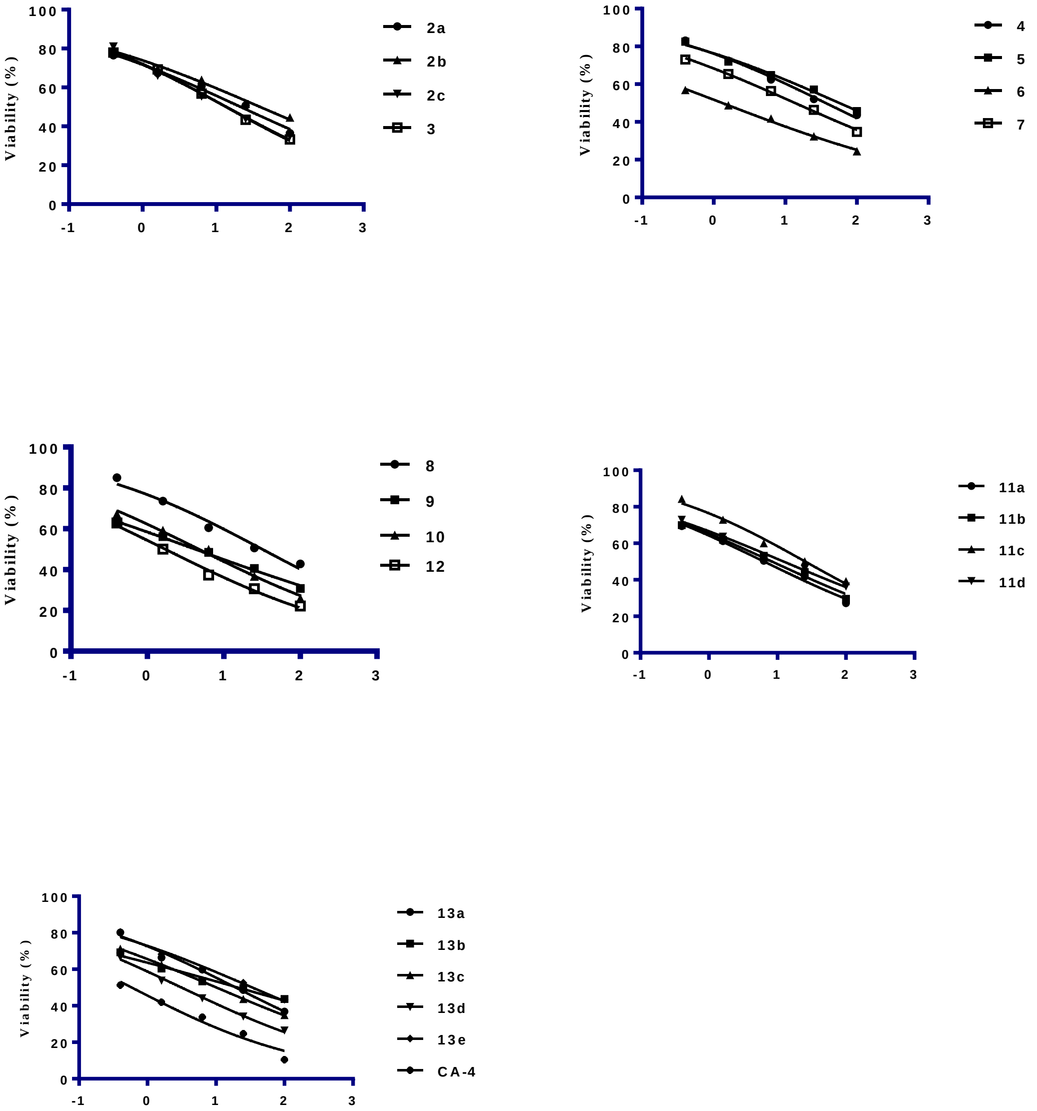

Figure S42: IC_{50} values of the prepared compounds.
